# *Methylacidiphilum fumariolicum* SolV, a thermoacidophilic ‘Knallgas' methanotroph with both an oxygen-sensitive and -insensitive hydrogenase

**DOI:** 10.1038/ismej.2016.171

**Published:** 2016-12-09

**Authors:** Sepehr Mohammadi, Arjan Pol, Theo A van Alen, Mike SM Jetten, Huub JM Op den Camp

**Affiliations:** 1 Department of Microbiology, IWWR, Radboud University, Heyendaalseweg 135, NL-6525 AJ, Nijmegen, The Netherlands

## Abstract

Methanotrophs play a key role in balancing the atmospheric methane concentration. Recently, the microbial methanotrophic diversity was extended by the discovery of thermoacidophilic methanotrophs belonging to the Verrucomicrobia phylum in geothermal areas. Here we show that a representative of this new group, *Methylacidiphilum fumariolicum* SolV, is able to grow as a real ‘Knallgas' bacterium on hydrogen/carbon dioxide, without addition of methane. The full genome of strain SolV revealed the presence of two hydrogen uptake hydrogenases genes, encoding an oxygen-sensitive (*hup*-type) and an oxygen-insensitive enzyme (*hhy*-type). The *hhy*-type hydrogenase was constitutively expressed and active and supported growth on hydrogen alone up to a growth rate of 0.03 h^−1^, at O_2_ concentrations below 1.5%. The oxygen-sensitive *hup*-type hydrogenase was expressed when oxygen was reduced to below 0.2%. This resulted in an increase of the growth rate to a maximum of 0.047 h^−1^, that is 60% of the rate on methane. The results indicate that under natural conditions where both hydrogen and methane might be limiting strain SolV may operate primarily as a methanotrophic ‘Knallgas' bacterium. These findings argue for a revision of the role of hydrogen in methanotrophic ecosystems, especially in soil and related to consumption of atmospheric methane.

## Introduction

Methanotrophs form an important sink for the greenhouse gas methane and they play a key role in keeping the atmospheric methane concentration in balance (Murrell and Jetten, 2009). They limit the amount of biologically or geochemically produced methane escaping into the atmosphere. The most studied group comprises the aerobic methanotrophs that phylogenetically belong to the Gammaproteobacteria and the Alphaproteobacteria (Hanson and Hanson, 1996). However, the methanotrophic diversity was extended when three research groups independently described novel thermoacidophilic methanotrophs isolated from geothermal areas in Italy, New Zealand and Russia (Dunfield *et al*, 2007; Pol *et al*, 2007; Islam *et al*, 2008). These novel isolates represented a distinct phylogenetic lineage within the phylum Verrucomicrobia. They belong to a single genus for which the name *Methylacidiphilum* was proposed (Op den Camp *et al*, 2009). Recently, a second genus of mesophilic, acidophilic verrucomicrobial methanotrophs was described, *Methylacidimicrobium* (van Teeseling *et al*, 2014). In addition, data from cultivation-independent environmental studies (Sharp *et al*, 2012; Sharp *et al*, 2014) indicated that methanotrophic Verrucomicrobia may be present in many more moderate-temperature volcanic ecosystems than assumed before. The discovery of a verrucomicrobial methanotroph was exciting, since for the first time the widely distributed Verrucomicrobia phylum, from which most members remain uncultivated, was coupled to a geochemical cycle (Pol *et al*, 2007). Initial analyses of our isolate *Methylacidiphilum fumariolicum* strain SolV showed major differences with the classical methanotrophs, for example extreme acid tolerance, absence of typical membrane structures, distinct enzymes and use of rare earth elements for methane oxidation (Pol *et al*, 2007; Pol *et al*, 2013; Keltjens *et al*, 2014).

It has long been assumed that methanotrophs are very strict in their diet, consuming only methane or methanol and occasionally other C1 compounds (Hanson and Hanson, 1996), despite the fact that most of the time a range of multi-carbon substrates is available in their environments (Tsien *et al*, 1989). Stimulation of growth by multi-carbon substrates like acetate, malate and succinate has been reported already by Whittenbury *et al*, 1970, but claims of growth on such substrates or even sugars for several isolated strains in subsequent years have been doubted (for a review see Semrau, 2011). Only in recent years convincing evidence has shown that acetate, pyruvate, ethanol, malate and succinate can be used for growth, but this seems restricted to alpha-proteobacterial methanotrophs (Kelly *et al*, 2000; Kolb, 2009; Kappler and Nouwens, 2013; Knief, 2015). The ecological significance especially of acetate consumption would be the increased survival of these methanotrophs under natural conditions, where methane concentrations are very low and variable (Belova *et al*, 2011). Moreover, methane oxidation might be possible at much lower concentrations when additional reducing equivalents from multi-carbon compounds are available, as speculated by Theisen and Murrell, 2005.

In many ecosystems another source of reducing equivalents is available as a potential energy source for methanotrophs, that is molecular hydrogen. The presence of putative hydrogenases has been detected in many different (sub)phyla; Proteobacteria, Firmicutes, Cyanobacteria, Aquificae, Euryarchaeota, Crenarchaeota and Verrucomicrobia (Greening *et al*, 2016). Hydrogenase-encoding genes were also identified in the genomes of multiple proteobacterial obligate methane oxidizers and uptake of hydrogen has been reported for *Methylosinus* sp.de Bont (1976), *Methylocystis* sp. and *Methylococcus capsulatus* Bath (Csáki *et al*, 2001; Kelly *et al*, 2005). In methanotrophs it is believed that hydrogenases also help to ‘save' reducing equivalents by recycling the hydrogen gas that is produced during nitrogen fixation (Bothe *et al*, 2010). Csáki and co-workers (Csáki *et al*, 2001) showed increased H_2_ production during nitrogen fixing conditions using a *hup*LS mutant of *M. capsulatus* Bath. Furthermore, soluble and membrane-bound hydrogenases have been reported for *M. capsulatus* Bath and hydrogen was shown to be able to supply reducing equivalents for the methane monooxygenase (Hanczár *et al*, 2002).

The fact that several proteobacterial methanotrophs contain genes encoding the large and small subunit of both uptake hydrogenases and the ribulose-1,5-bisphosphate carboxylase (Rubisco) suggests the possibility of autotrophic growth. But attempts to grow *M. capsulatus* Bath autotrophically (on hydrogen and carbon dioxide) in liquid media were not successful (Dalton and Whittenbury, 1976; Taylor *et al*, 1981; Stanley and Dalton, 1982; Baxter *et al*, 2002). Autotrophic growth was observed on solid agar media, but no physiological studies have been reported to support this (Baxter *et al*, 2002).

*M. fumariolicum* SolV was shown to use the Calvin-Benson-Bassham (CBB) cycle for carbon fixation pathways (Khadem *et al*, 2011), and is capable of fixing nitrogen gas (Khadem *et al*, 2010). Key metabolic genes of the ribulose monophosphate and serine pathways are absent (Pol *et al*, 2007). The full genome of strain SolV (Anvar *et al*, 2014) revealed the presence of two hydrogenase types (*hup*- and *hhy*-type). Many Group 1 hydrogenases within the *hup* type are known to support growth. While in general hydrogenases are very sensitive towards oxygen and function only in anaerobic respiration, the Group 1d hydrogenases are known for their relative oxygen tolerance and may support aerobic growth in ‘Knallgas' bacteria. The *hhy* type genes encode the recently discovered Group 5 hydrogenases, which are widespread in actinobacteria from soil and were supposed to be responsible for ‘high affinity' atmospheric hydrogen uptake (Schäfer *et al*, 2013; Greening *et al*, 2014). The ‘Knallgas' bacterium *Ralstonia eutropha* also contains this type of hydrogenase that upon isolation appeared to be oxygen insensitive (Schäfer *et al*, 2013). A role for this enzyme is as yet not established. When hydrogen was added to methane-consuming cultures of *M. fumariolicum* SolV, the hydrogen was also oxidized (Pol *et al*, 2007). RNA-seq analysis of SolV cells at maximal growth rate (μ_max_) compared to nitrogen fixing and oxygen limiting conditions showed up-regulation of the *hup*-type hydrogenase at low oxygen concentrations, whereas the *hhy*-type enzyme was constitutively expressed (Khadem *et al*, 2012a). The presence of the CBB cycle, together with the uptake hydrogenase prompted us to investigate the possibility to grow strain SolV as a real ‘Knallgas' bacterium without addition of methane. We used physiological experiments in combination with phylogenetic and transcriptome analysis to show that strain SolV can grow autotrophically on hydrogen and carbon dioxide.

## Materials and methods

### Microorganism and medium composition

*Methylacidiphilum fumariolicum* strain SolV used in this study was initially isolated from the volcanic region Campi Flegrei, near Naples, Italy (Pol *et al*, 2007). In this study the medium was composed of 0.2 mM MgCl_2_.6H_2_O; 0.2 mM CaCl_2_.2H_2_O; 1 mM Na_2_SO_4_; 2 mM K_2_SO_4_; 2 mM (NH_4_)_2_SO_4_ (or 5 mM KNO_3_) and 1 mM NaH_2_PO_4_.H_2_O. A trace element solution containing 1 μM Ni, Co, Mo, Zn and Ce; 5 μM Mn and Fe; 10 μM Cu and 40–50 μM nitrilotriacetic acid. The pH of medium was adjusted to 2.7 using 1 M H_2_SO_4_. To avoid precipitation, CaCl_2_.2H_2_O and the rest of the medium were autoclaved separately and mixed after cooling. This medium composition contains all nutrients to obtain an OD_600_ of 1.0 and was used in batch and continuous cultures, unless stated otherwise.

### Chemostat cultivation

To start a continuous culture on hydrogen, we initiated growth on methane, while hydrogen was absent. The reactor (Applikon Biotechnology, Delft, NL) was operated at 55 °C with stirring at 1000 rpm. The chemostat (liquid volume of 1.2 L) was supplied with medium at a flow rate of 30 ml h^−1^ (D=0.025 h^−1^). A gas supply of 50% CH_4_ (v/v) and 5% CO_2_ (v/v) was provided by mass flow controllers through a sterile filter and sparged into the medium at a gas flow rate of 16–17 ml min^−1^. The dO_2_ was regulated initially at 1% oxygen saturation for 18 h. Afterwards, the dO_2_ set-point was adjusted to 0.2% oxygen for 9 h and an OD_600_ value of 0.85 was obtained. At this point, methane was changed to hydrogen maintaining the total gas flow rate identical to the methane condition and keeping the dO_2_ value at 0.20–0.22% oxygen. The cell-containing medium was removed automatically from the chemostat by a peristaltic pump when the liquid level reached the sensor in the reactor. The pH was regulated in the steady state at 3.0 using 0.2 M NaOH.

The continuous culture with CH_4_ as an electron donor and nitrate (NO_3_^−^) as N-source had a liquid volume of 550 ml, and was operated at 55 °C with stirring at 900 rpm with a stirrer bar. The chemostat was supplied with medium at a flow rate of 14.5 ml h^−1^ (D=0.026 h^−1^). The cell-containing medium was removed automatically from the reactor by a peristaltic pump when the liquid level reached the sensor in the reactor. Supply of 10% CH_4_ (v/v), 8% O_2_ (v/v) and 68% CO_2_ (v/v) took place by mass flow controllers (total flow≈20 ml min^−1^) through a sterile filter and sparged into the medium just above the stirrer bar. The pH was regulated at 2.7 using a 1 M solution of bicarbonate. An O_2_ sensor in the liquid was coupled to a Biocontroller (Applikon) regulating the O_2_ mass controller.

### Batch cultivation

To obtain maximum growth rate (μ_max_), cells were grown without any limitation in 250-ml serum bottles containing 40 ml medium (4 mM NH_4_^+^; pH 2.7), and sealed with red butyl rubber stoppers. The headspace contained air with (v/v) 10% CH_4_, 5% CO_2_ and cultures were incubated at 55 °C with shaking at 250 rpm. Incubations were performed in duplicate and exponentially growing cells (OD_600_=1) were collected for mRNA isolation and Ion Torrent sequencing.

### Dry weight determination and elemental analysis

To determine biomass dry-weight concentration, 10 ml of the culture suspension (triplicate) were filtered through pre-weighed 0.45 μm filters and dried to stable weight in a vacuum oven at 60 °C. In order to determine the total content of carbon and nitrogen, 10 ml of the culture suspension (duplicate) was centrifuged at 4500 *g* for 30 min and the clear supernatant was used for the analysis. The nitrogen and carbon content in the supernatant was compared with the corresponding values in the whole cell suspension. The total carbon and nitrogen contents were measured using TOC-L and TNM-1 analysers (Shimadzu, Kyoto, Japan).

### Respiration experiments

Respiration rates were measured polarographically in a respiration cell with an oxygen microsensor (RC350, Strathkelvin, Motherwell, UK) using 3 ml of whole cell suspensions of strain SolV. Methane, hydrogen or oxygen-saturated medium were injected to the respiration chamber to obtain the desired dissolved gas concentrations. The O_2_ signal was monitored and recorded using SensorTrace Basic software (Unisense, Aarhus, Denmark). The temperature and stirring rate in the respiration chamber was adjusted to 55 °C and 1000 rpm, respectively. Rates were expressed as nmol O_2_.min^−I^.mg DW^−1^ and when necessary corrected for endogenous respiration. To avoid too high oxygen concentrations at the start of an experiment, samples taken from cultures were immediately transferred into rubber septum sealed bottles under an anoxic atmosphere of nitrogen and carbon dioxide. These bottles contained medium in case dilution was necessary. A subsample was taken from the bottle by a syringe with a long needle and introduced into the respiration chamber at the bottom with the oxygen probe in place while pushing out the air via the inlet channel.

### Hydrogenase activity assays

Three ml of cell suspension of strain SolV (OD_600_ 0.3–0.4/mg DW) was incubated under an atmosphere of N_2_—CO_2_—O_2_ (80%: 20%: 0.4%, v/v/v) in 60-ml capped serum bottles at 55 °C and shaking at 400 rpm. Samples from cultures growing on hydrogen were preincubated under the same conditions for 30 min in order to consume any hydrogen present in the samples. The consumption of added hydrogen was measured using an HP 5890 gas chromatograph (Agilent, Santa Clara, USA) equipped with a Porapak Q column (1.8 m, ID 2 mm) and a thermal conductivity detector. For all gas analyses 250 μl gas samples were injected with a glass syringe.

### Phylogenetic analysis

The gene sequences of the large and small subunit of uptake hydrogenases from different strains including *Methylacidiphilum* and *Methylacidimicrobium* species were downloaded from NCBI-GenBank. Conceptual translations into amino acids were performed and used for creating an alignment and phylogenetic analysis using MEGA 6 software (Tamura *et al*, 2013). The evolutionary history was interpreted using the neighbour-joining method (Saitou and Nei, 1987).

### RNA isolation and sequencing

The complete genome sequence of strain SolV (Anvar *et al*, 2014), which is also available at the MicroScope annotation platform (https://www.genoscope.cns.fr/agc/microscope/home/), was used as the template for the transcriptome analysis (RNA-seq). A 4-ml volume of cells (OD_600_=1) was sampled from the continuous cultures (H_2_- and CH_4_-grown cells under O_2_ limitation) and from a batch culture (cells at μ_max_ grown on methane without limitation) and harvested by centrifugation. The pellet was further used for mRNA isolation using the RiboPure™-Bacteria Kit according to the manufacturer's protocol (ThermoFisher, Waltham, USA). Briefly, cells were disrupted by cold Zirconia beads and after centrifugation 0.2 volumes of chloroform was added to the supernatant for initial RNA purification. Next, 0.5 volumes of 100% ethanol was added to the aqueous phase obtained after chloroform addition and the whole sample was transferred to a filter cartridge. After washing, the RNA was eluted from the filter cartridge. Afterwards, using MICROB *express*™ kit (ThermoFisher) the ribosomal RNAs were removed from the total RNA. The rRNA removal efficiency was checked using the Agilent 2100 Bioanalyzer (Agilent). Next, Ion Total RNA-Seq Kit v2 (ThermoFisher) was used to construct the cDNA libraries from rRNA-depleted total RNA. Briefly, the rRNA-depleted total RNA was fragmented using RNase III and then, reverse transcription was performed on the fragmented RNAs. The obtained cDNAs were amplified and further purified to prepare barcoded libraries. To prepare the template for the Ion Personal Genome Machine (PGM) System, a volume of 15 μl from two sample libraries with a concentration of 14 pM were mixed. This mixture of two libraries was used to prepare the template-positive Ion Sphere particles (ISPs) using the Ion OneTouch 2 instrument. Afterwards, the template-positive Ion Sphere particles were enriched using the Ion OneTouch ES instrument. Both template preparation and enrichment were performed using the Ion PGM Template OT2 200 Kit (Ion Torrent, Life technologies). Enriched templates were sequenced on an Ion 318 Chip v2 using the Ion PGM sequencing 200 Kit v2. Expression analysis was performed with the RNA-seq Analysis tool from the CLC Genomic Work bench software (version 7.0.4, CLC-Bio, Aarhus, Denmark) and values are expressed as RPKM (reads per kilo base of exon model per million mapped reads) (Mortazavi *et al*, 2008).

## Results

### Hydrogen oxidation by *M. fumariolicum* SolV cells grown on methane

Cells from batch cultures of strain SolV growing on methane at maximum growth rate (μ=0.07 h^−1^) and oxygen concentrations above 10% showed relatively high oxygen consumption rates at the expense of hydrogen, 15–20 nmol.min^−1^.mg DW^−1^, which was about 6% of the oxygen consumption with methane (280 nmol.min^−1^.mg DW^−1^; Table 1). However, growth on hydrogen and carbon dioxide without methane under such conditions was not possible. Initial batch tests in bottles showed growth only at (and below) 1% O_2_ concentrations. Since the oxygen consumption results in a rapid decrease of the oxygen concentration during batch cultivation, we studied the inhibiting effects of oxygen in a continuous culture for which the dO_2_ was regulated carefully with mass flow controllers. The hydrogen respiration rate of a continuous culture under methane limitation (D=0.03 h^−1^) was measured at different oxygen concentrations. The initial dO_2_ was regulated at 0.3% oxygen (1.5% air) and the hydrogen respiration rate was slightly higher than those in batch cultures (20–24 nmol O_2_.min^−1^.mg DW^−1^; Table 1), being about 7.5% of the methane respiration rates (268–317 nmol O_2_.min^−1^.mg DW^−1^; Table 1). When the dO_2_ was stepwise increased to a final value of 3.2% oxygen (16% air; each step was stabilized for at least one day), respiration rates were not significantly affected. Similarly, measurements in the respiration chamber showed that rates were more or less the same at all dO_2_ values and stable during measuring periods of about 2 h. This is a strong indication that the *hhy*-type hydrogenase responsible for this activity was constitutively expressed under these conditions and most probably oxygen insensitive.

When a methane-fed continuous culture was grown under oxygen limitation (D=0.015 h^−1^), hydrogen respiration measurements became less straightforward with inactivation and reactivation events occurring (see Supplementary Figure S1). Hydrogen respiration rates measured were at least twice the rate obtained for the continuous culture under methane limitation at dO_2_ values ranging from 0.3% to 3.2% oxygen (1.5–16% air; described above). Therefore, it seems that under oxygen limitation the oxygen-sensitive *hup*-type hydrogenase is expressed in addition to the constitutively expressed oxygen-insensitive *hhy*-type.

### Autotrophic growth of *M. fumariolicum* SolV on hydrogen

A batch culture growing at μ_max_ (0.075 h^−1^) in a bioreactor on methane at low oxygen concentration (regulated at 0.2% oxygen by mass flow controllers) also showed increased hydrogen oxidizing activity. When the gas supply was changed from methane to hydrogen only, the oxygen consumption rate dropped and stabilized after 45 min at 13% of the oxygen consumption rate with methane. This hydrogen respiration rate is about double compared to batch cultures at high oxygen concentration (which was 6% of the methane respiration, see above) and also clearly higher than the rate observed in the continuous culture at dO_2_ above 0.3% oxygen (which was 7% of the methane respiration rate, see above). The culture immediately continued growing, since we observed a 6% biomass increase 3 h after the switch from methane to hydrogen (OD_600_ increased from 0.85 to 0.9). The only carbon source during growth on hydrogen was carbon dioxide.

**Figure 1:**
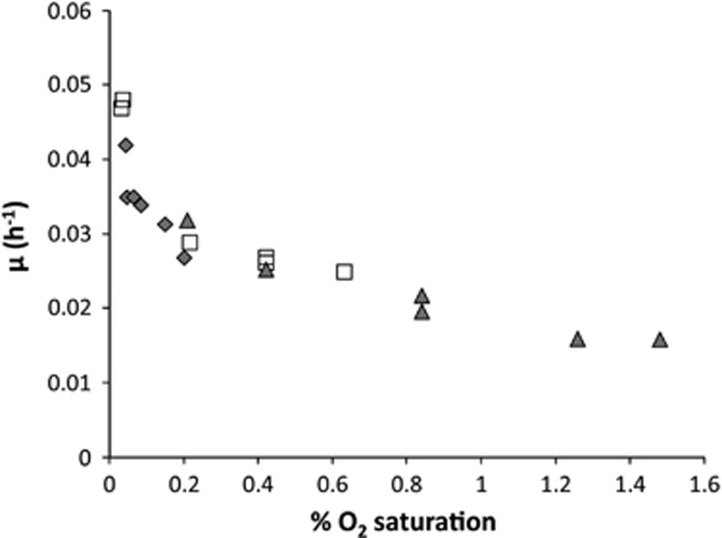
The effect of oxygen on the growth rate of H_2_-growing cells. 1% of O_2_ saturation is equal to 7.5 μM O_2_ in the liquid. Each set of symbols represents a newly started experiment in which cells were grown batch-wise up to maximum OD_600_=4 after which the culture was diluted to OD_600_ 0.1–0.2 and restarted as a new batch.

Results thus far indicated a critical oxygen concentration of about 0.2%. Below this value the oxygen-sensitive *hup*-type hydrogenase seems to be expressed. Therefore, the oxygen effect on growth rate was investigated in more details in a bioreactor under excess of hydrogen in which dO_2_ could be accurately controlled at values as low as 0.04% oxygen (equivalent to 0.3 μM O_2_). First growth was achieved batch-wise up to maximum OD_600_ values of 4 after which the culture was diluted to OD_600_ 0.1–0.2 and restarted as a new batch. The dO_2_ was stepwise changed starting at 0.2% oxygen. Figure 1 shows the growth rates that were calculated when at least one generation of stable exponential growth was observed (based on OD_600_ measurements). The values clearly show the oxygen sensitivity of the growth on hydrogen. Highest growth rates were observed at the lowest oxygen concentrations. Decreasing the dO_2_ from 0.2% to 0.04% oxygen resulted in a sharp increase of the growth rate while above 0.2% oxygen, a steady decrease of the growth rate was observed. Up to a dO_2_ value of 1.5% oxygen we obtained stable growth rates. At a dO_2_ value of 2% oxygen, the growth continued for a few generations but the growth rate dropped continuously. In respiration experiments, O_2_ consumption was almost constant as observed in samples from the methane-limited continuous culture (only a slight reactivation was observed below 10 μM O_2_). Similarly, batch experiments with bottles showed growth only at (and below) 1% O_2_ concentrations (no growth was observed at 2.5 and 5% O_2_ concentrations).

Cultures at high growth rate exhibited both oxygen-sensitive and -insensitive hydrogenase activity during respiration experiments, similar to our observations for the O_2_-limited culture growing on methane (see above). Cells sampled from the batch cultures growing on hydrogen at the highest growth rate obtained (μ=0.047 h^−1^) were transferred to the respiration chamber avoiding O_2_ exposure. The respiration rates with hydrogen measured were as high as 66–71 nmol O_2_.min^−1^.mg DW^−1^ (when actual O_2_ concentration in the respiration chamber was below 5 μM; Table 1). When samples were incubated in bottles under an atmosphere of 0.5% hydrogen and 0.1% or 0.2% oxygen, the hydrogen consumption rate, analysed by gas chromatography, was 220 nmol H_2_.min^−1^.mg DW^−1^. This correlated very well with the oxygen consumption rate when assuming a H_2_:O_2_ consumption ratio of 0.32 as was determined during respiration experiments. When cells of a culture growing under similar conditions (continuous culture on hydrogen at D=0.04 h^−1^) were exposed to air the hydrogen respiration rate (measured at 100 μM O_2_) was only about 17 nmol O_2_.min^−1^.mg DW^−1^ and this rate was increasing again when O_2_ fell below 50 μM (Table 1). Methane respiration rates of cultures when changed from methane to exclusively hydrogen remained high (>171 nmol O_2_.min^−1^.mg DW^−1^) for many generations (>20). However, when a continuous culture was grown for over a year on hydrogen only, the methane respiration rate had dropped to 29–39 nmol O_2_.min^−1^.mg DW^−1^.

### Yield

An oxygen-limited continuous culture on hydrogen (D=0.026 h^−1^) was used to assess the growth yield by accurately measuring the hydrogen consumption. Based on dry weight (DW) measurements a yield value of 3.4±0.1 g DW.mole H_2_^−1^ was obtained. Organic carbon analysis of centrifuged culture samples revealed the presence of 8–10% of the total organic matter in the supernatant, comparable to the amount excreted when growing on methane. The stoichiometry of hydrogen respiration was assessed both by the oxygen respiration measurements (H_2_ vs O_2_) and gas chromatographic analysis (for H_2_ vs CO_2_) measurements and resulted in the following stoichiometry:


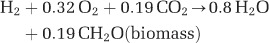


### Kinetics of hydrogen consumption

**Figure 2:**
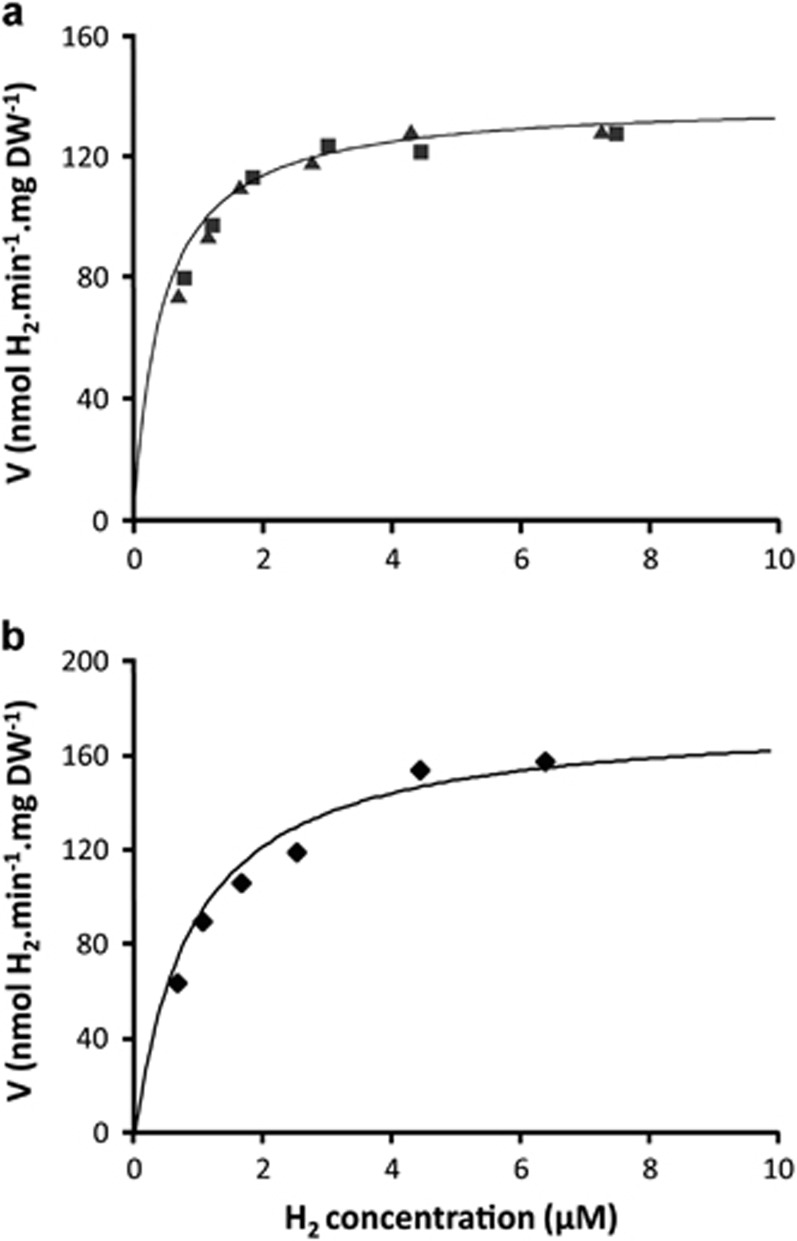
(**a**) The kinetics of the oxygen-insensitive hydrogenase of the cells grown on hydrogen in a batch condition at dO_2_ value at 0.8% (V_max_=127 nmol H_2_.min^−1^.mg DW^−1^; K_s_=0.6 μM). Closed squares and triangles represent two independent experiments. (**b**) The kinetics of both the oxygen-sensitive and -insensitive hydrogenases of cells grown on hydrogen in a batch condition at dO_2_ value below 0.2% (V_max_=159 nmol H_2_.min^−1^.mg DW^−1^; Ks=1.1 μM). Closed diamonds represent the results of three independent experiments. The solid line represents the best Michaelis–Menten kinetics fitting curve to our experimental data in both (a) and (b).

Addition of hydrogen to a methane-limited culture (D=0.013 h^−1^, at dO_2_ 0.5–1%) resulted in an immediate increase of biomass (DW) and both substrates were consumed simultaneously. These conditions favour expression of the oxygen-insensitive hydrogenase. From respiration experiments it was observed that the maximum respiration rate for methane was much higher than for hydrogen. For methane the rate dropped when reaching concentrations below 15 μM, in accordance with the affinity constant for methane (K_s_ 6 μM) (Pol *et al*, 2007). In contrast hydrogen respiration rates remained constant until hydrogen was consumed completely (Supplementary Figure S2). It was estimated that this equals a hydrogen concentration well below 1 μM. The kinetics for the oxygen-insensitive hydrogenase were more accurately assessed in cells grown on hydrogen in batch at dO_2_ value at 0.8% (D=0.02 h^−1^) by measuring hydrogen consumption rates in bottles at various hydrogen concentrations. When Michaelis–Menten kinetics were fitted (Figure 2), an apparent hydrogen K_s_ of 0.6 μM was obtained (measured V_max_=127 nmol H_2_.min^−1^.mg DW^−1^). Hydrogenase kinetics of cells growing at lower dO_2_ of 0.2%, conditions (D=0.03 h^−1^) in which additional expression of the oxygen-sensitive hydrogenase is expected, showed a higher hydrogen consumption rate (V_max_=159 nmol H_2_.min^−1^.mg DW^−1^) but also a higher affinity constant value. Results for this mixed activity of hydrogenases were less accurate, but a K_s_ value of 1.1 μM H_2_ was estimated (Figure 2). The contribution of the oxygen-sensitive hydrogenase to the maximum measured respiration rates was estimated to be about two times higher than that of the oxygen-insensitive hydrogenase and by simulating Michaelis–Menten kinetics we estimated that the apparent affinity constant of the oxygen-sensitive hydrogenase was between 1 and 2 μM. The hydrogen consumption by SolV cells grown on methane under oxygen limitation (D=0.018 h^−1^) was followed in bottles with hydrogen concentrations of 0.5 μM (dO_2_ in bottles 0.2%). The consumption proceeded according to first order kinetics down to the GC detection level, that is 2 ppm hydrogen in the headspace (equivalent to 1.5 nM).

### Hydrogenases in the genomes of verrucomicrobial methanotrophs

The full genome of strain SolV was analysed for hydrogenase-encoding genes (including accessory genes) and for comparison identical genes in the full and draft genomes of other verrucomicrobial methanotrophs were identified (Hou *et al*, 2008; Sharp *et al*, 2012; Sharp *et al*, 2014; van Teeseling *et al*, 2014; Erikstad and Birkeland, 2015). Both thermophilic and mesophilic strains contain a hydrogenase encoded by the genes *hup*S (small subunit) and *hup*L (large subunit; Supplementary Table S1). In addition, we identified a second hydrogenase encoded by the genes *hhy*S (small subunit) and *hhy*L (large subunit), only present in the thermophilic strain SolV and its closest relative *M. kamchatkensis* Kam1 (Supplementary Table S1). Survey of the genomes also revealed an electron transport unit (*cyt*B) and several accessory genes (Supplementary Tables S2 and S3).

**Figure 3:**
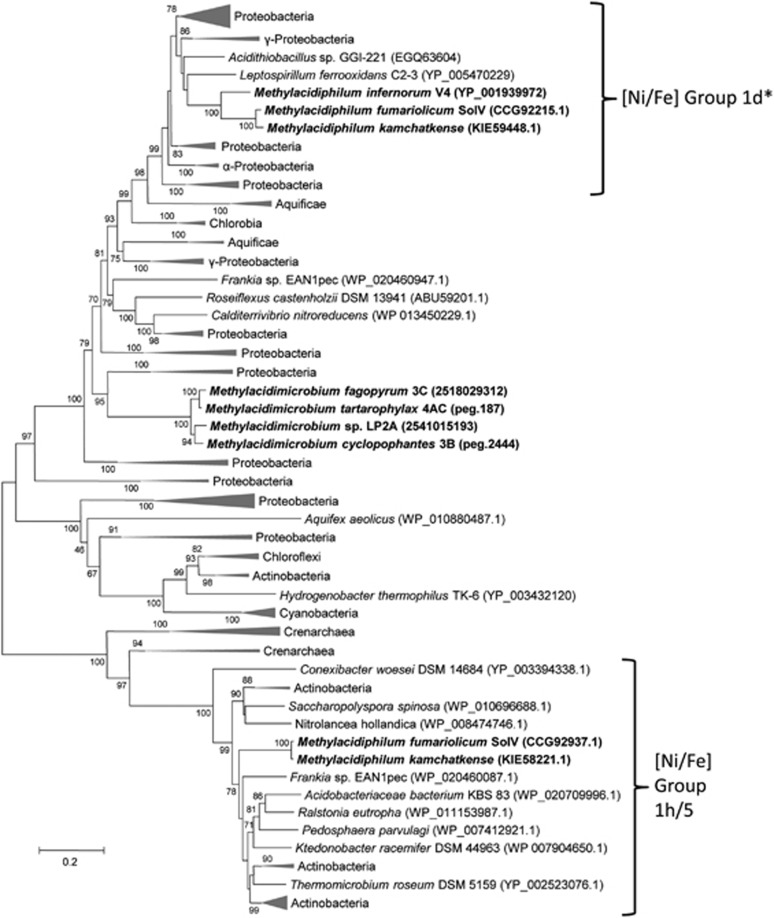
The uptake hydrogenase gene-based phylogenetic tree of more than 130 bacterial species showing both thermophilic and mesophilic strains contain a hydrogenase encoded by the genes *hup*S (small subunit) and *hup*L (large subunit). In addition, a second hydrogenase encoded by the genes *hhy*S (small subunit) and *hhy*L (large subunit) were identified only in the thermophilic strain SolV and the close relative strain Kam1. The evolutionary history was generated using the neighbour-joining method. The percentage of replicate trees in which the associated taxa clustered in the bootstrap test is shown next to the branches. The analysis involved 136 nucleotide sequences. Evolutionary analyses were conducted in MEGA6. (*) indicates four exceptions including *Thiorhodococcus drewsii AZ1, Thiocapsa marina, Salmonella enterica and Methylocystis parvus,* which fit to Group 1e and 1c, in addition to Group 1d hydrogenases.

Phylogenetic analysis using the recent classification (Vignais and Billoud, 2007; Greening *et al*, 2016) showed that the *hup*-type hydrogenases of the verrucomicrobial methanotrophs belong to the membrane-bound H_2_-uptake [NiFe]-hydrogenases (Figure 3; Group 1). The large subunits all contain L1 and L2 motifs typical for Group 1 hydrogenases.

The second *hhy*-type hydrogenase belongs to Group 1 h/5 hydrogenases (Figure 3), based on the phylogenetic analysis of both the small and large subunits. The large subunit contains active site motifs L1 (TSRICGICGDNH) and L2 (SFDPCLPCGVH) that are highly conserved in other Group 1 h/5 hydrogenases.

**Figure 4:**
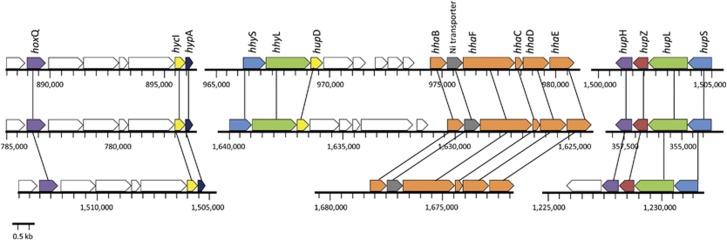
Gene arrangement of the hydrogenases in the thermophilic *Methylacidiphilum* strains SolV (top), Kam1 (middle) and V4 (bottom). Genes are colour coded as follows: green=large subunit; blue=small subunit; yellow=putative hydrogenase maturation protease; orange=accessory proteins; red=b-type cytochrome subunit; purple=putative expression/formation protein; grey=nickel transporter; dark blue=nickel insertion protein.

Figure 4 shows the gene arrangement of the two hydrogenase types of strain SolV. A first cluster includes the genes encoding the Group 1 h/5 hydrogenase (*hhy*S and *hhy*L), together with the gene encoding the putative maturation like protein (*hup*D). In close proximity (4.6 kb) the genes encoding accessory proteins (*hha*BFCDE) are clustered together with a gene encoding a high affinity nickel transporter. At another location in the genome we identified genes encoding a putative expression/formation protein (*hox*Q), a putative hydrogenase maturation protease (*hyc*I) and the nickel insertion protein (*hyp*A).

The second hydrogenase cluster encoding the Group 1d type (Figure 4) includes the genes for the large and small subunit (*hup*SL), the expression/formation protein (*hup*H) and the b-type cytochrome subunit (*hup*Z). Comparison of the gene arrangement with the other two thermophilic strains (Figure 4) revealed an almost identical presence and clustering in strain Kam1. The genome of strain V4 does not seem to have the genes encoding the Group 1 h/5 hydrogenase but the clustering of the other genes encoding the Group 1d hydrogenase and accessory proteins is identical to strain SolV and Kam1 (Figure 4). In the mesophilic *Methylacidimicrobium* strains for which the most complete draft genomes are available (strains LP2A and 3C) the genes encoding the Group 1d-type hydrogenase show an identical gene clustering compared to the thermophilic *Methylacidiphilum* strains (Supplementary Figure S3). Unlike the thermophilic strains, the genes encoding the accessory proteins are in close proximity to the functional genes, but on the opposite strand. For the other mesophilic strains 4AC and 3B no comparison can be made since these draft genomes are too fragmented.

### Transcriptome analysis

Expression levels of housekeeping genes and genes involved in hydrogen metabolism were determined for hydrogen- and methane-grown cells (both under O_2_-limited conditions). These values were compared to the expression values in cells growing at μ_max_ with methane (without limitation). To determine the expression levels of the different genes under different conditions, the SolV transcriptome was characterized using IonTorrent PGM sequencing of ribosomal RNA depleted total mRNA (RNA-Seq). The sequencing reads were first mapped to the ribosomal RNA operon and all tRNA genes, and mapped reads were discarded. The remaining reads were mapped to the gene coding sequences extracted from the genome sequence of strain SolV (Anvar *et al*, 2014). The total number of reads obtained and mapped on the coding sequences of the genome for each sample together with the calculated expression levels (RPKM) is provided in the supplementary material (Supplementary Table S4). To compare baseline expression levels, we selected a group of 393 housekeeping genes (in total 442.8 kbp) (Khadem *et al*, 2012a, b) involved in energy generation, ribosome assembly, carbon fixation (CBB cycle), C1 metabolism (except for *pmo*), amino acid synthesis, cell wall synthesis, translation, transcription, DNA replication and tRNA synthesis. All ratios of expression levels of the housekeeping genes among these conditions were between 0.5 and 2 (Supplementary Table S5). The robustness of the transcriptome data were tested using the method of Chaudhuri *et al* (2011). In this method, the logarithmic value of (RPKM+1) of each condition (in duplicates) was calculated and the values were plotted against each other. This resulted in correlation coefficients of 0.80, 0.82 and 0.87 (Supplementary Figure S4), showing the high robustness of the transcriptome data.

We showed that two uptake hydrogenase encoding genes (*hup*L and *hup*S) were upregulated in both hydrogen- and methane-grown cells (both under O_2_ limitation) compared to cells growing at μ_max_. Furthermore, the expression of these genes in hydrogen-grown cells were about twofold higher compared to methane-grown cells (Table 2). The transcription of the gene encoding the [NiFe] hydrogenase b-type cytochrome subunit was significantly upregulated in the hydrogen-grown cells compared to the cells grown at μ_max_ with methane (about 23-fold). This b-type cytochrome subunit is known to be coupled with membrane-bound hydrogenases and involved in the energy conservation. Moreover, the putative *hup*H hydrogenase expression gene was upregulated in the cells grown on hydrogen compared to cells grown at maximum growth rate with methane (about 22-fold). On the other hand, the genes encoding the Group 1 h/5 type hydrogenase (*hhyL* and *hhyS*) did not show different expression levels in cells grown under the three conditions tested. The same holds for the genes encoding the accessory proteins. This points to the Group 1d type [NiFe] hydrogenase being the inducible oxygen-sensitive hydrogenase.

Furthermore, the transcriptome data showed that the hydrogen treatment had no significant influence on expression levels of the Rubisco subunits. The genes encoding the small (*cbbS*) and large subunit (*cbbL*) were highly expressed under all conditions tested (Table 2; Supplementary Table S4), supporting autotrophic growth on hydrogen and carbon dioxide.

## Discussion

In their natural ecosystems the methanotroph *Methylacidiphilum fumariolicum* SolV and its close relatives encounter both methane and hydrogen under acidic (pH 1–2) and hot (50–70 °C) conditions with low availability of oxygen (Chiodini *et al*, 2001; Pol *et al*, 2007). Physiological experiments with both batch and continuous culture of strain SolV described in this study showed that this bacterium can grow autotrophically on hydrogen and carbon dioxide, using the ‘Knallgas' reaction to gain energy and the Rubisco pathway for carbon fixation. No supply of methane is needed. When growing on methane, hydrogen oxidation was possible under ambient oxygen concentration, but growth on hydrogen and carbon dioxide was possible only below 1.5% oxygen. Highest growth rates were obtained when oxygen was very low (dO_2_<0.1%). The maximum specific growth rate on hydrogen (0.047 h^−1^) is about 60% compared to the growth rate on methane (Pol *et al*, 2007). Assuming the same CO_2_:H_2_ consumption ratio as measured at D=0.0124 h^−1^, such a growth rate needs a specific hydrogenase activity of about 175 nmol H_2_.min^−1^.mg DW^−1^ (the carbon content of DW was 51%). This matches well with the *in vitro* measured maximum hydrogen uptake rates (220 nmol H_2_.min^−1^.mg DW^−1^). The measured yield on hydrogen is about half that on methane (3.4 vs 6.4 g DW/mole CH_4_) (Pol *et al*, 2007) and slightly lower than those reported for ‘Knallgas' bacteria like *Ralstonia eutropha* (4.6 g/mole H_2_) (Morinaga *et al*, 1978) or *Hydrogenomonas eutropha* (5 g/mole H_2_) (Bongers, 1970).

Continuous cultures fed with hydrogen under low oxygen conditions remained stable for more than a year. Although previous studies have shown that co-oxidation of hydrogen together with methane was possible (de Bont, 1976; Hanczár *et al*, 2002), to our knowledge this is the first time that a methanotroph is shown to grow on hydrogen/carbon dioxide only in liquid mineral medium. Baxter *et al* (2002) reported growth with hydrogen and carbon dioxide on agar plates but not in liquid cultures for several methanotrophs but autotrophy was not substantiated by physiological and biochemical evidence. The presence of a fully operational Calvin cycle with Rubisco as the key enzyme in verrucomicrobial methanotrophs seems to be a pre-requisite for autotrophic growth on hydrogen.

Recently, it has been shown that microorganisms from a wide diversity of ecosystems, ranging from the hypoxic hydrogen-rich habitats of animal guts and bog soils to aerated soils and waters containing small quantities of H_2_, possess genes encoding different types of hydrogenases (Greening *et al*, 2016). We showed that *Methylacidiphilum* sp. and *Methylacidimicrobium* sp. representing the acidophilic verrucomicrobial methanotrophs (Op den Camp *et al*, 2009; Sharp *et al*, 2014; van Teeseling *et al*, 2014) contain genes encoding hydrogen uptake hydrogenases. According to the phylogeny of both the large and small subunits, the *hup*-type hydrogenases in the verrucomicrobial methanotrophs belong to the membrane-bound H_2_-uptake [NiFe]-hydrogenases (Group 1d), and the *hhy*-type hydrogenases (present only in strains SolV and Kam1) belong to Group 1 h/5 hydrogenases as they are not membrane anchored (Greening *et al*, 2016). Based on the phylogenetic analysis, we showed that several alpha- and gammaproteobacterial methanotrophic species contain hydrogenase genes, and growth with hydrogen and carbon dioxide was reported on agar plates but not in liquid cultures for several methanotrophs (Baxter *et al*, 2002). In methanotrophic bacteria that cannot fix CO_2_, hydrogenases may help to save reducing equivalents by recycling the hydrogen gas that is produced during nitrogen fixation, or supply reducing equivalents for the methane monooxygenase (Csáki *et al*, 2001; Hanczár *et al*, 2002; Bothe *et al*, 2010).

The small subunits of the *hup*-type hydrogenases contain the typical twin-arginine signal peptide distinctive for periplasmic membrane-bound Group 1 hydrogenases (Palmer and Berks, 2012). The *hup*-type hydrogenases of the thermophilic strains have features typical for Group 1d. Their large subunit contains the typical L1 (xxRICGVCTxxH) and L2 (SFDPCLACxxH) motifs and their small subunit contains the conserved motif with two extra cysteines (Greening *et al*, 2016). In *Ralstonia eutropha* these extra cysteines have been shown to ligate to an ‘exclusive' proximal 4Fe3S cluster that is supposed to render hydrogenases extra oxygen tolerance (Fritsch *et al*, 2011). In contrast, HupS subunits of the mesophilic strains do not contain these extra cysteines. Instead cysteines seem to be arranged to coordinate a 4Fe4S proximal cluster like most Group 1 members (not being the Group 1d type), although one of the four cysteines is replaced by an asparagine. A similar change is also observed in Group 1f hydrogenases of *Roseoflexis*, *Geobacter* and *Frankia* spp. where the cysteine is replaced by asparagine or aspartic acid. Asparagine coordination to 4Fe4S is also present in Group 2a hydrogenases. Conserved cysteines for the medial FeS cluster (3Fe4S coordinating with three cysteines, as in Group 1b–g) and distal FeS cluster (4Fe4S, coordinating with three cysteines and one histidine, as in Group 1a–h) are present in the HupS of both mesophilic and thermophilic strains. The Group 1d hydrogenase in strain SolV was shown to be responsible for increased growth rates when dO_2_ level was below 0.2% (see above).

Furthermore, we showed that genes encoding an additional hydrogenase are present in the thermophilic strains SolV and Kam1. Based on the phylogenetic analysis of both its small and large subunits, this hydrogenase belongs to Group 1h/5 hydrogenases. They contain active site L1 (TSRICGICGDNH) and L2 (SFDPCLPCGVH) motifs that are highly conserved in Group 1h/5 hydrogenases and distinct from other Group 1–4 hydrogenases (Greening *et al*, 2016). These hydrogenases are located at the cytoplasmic side of the membrane, since they lack the twin-arginine signal peptide in the small subunit. The small subunit also lacks the C-terminal membrane span alpha helix extension to anchor in a membrane. The proximal FeS cluster is reminiscent to the mesophilic counterpart (discussed above) and others in Group 1b, f, g in that it has only three cysteines available for coordination. The fourth position for a 4Fe4S cluster would then be the conserved Asp (de Bont, 1976) as shown for the Group 1h/5 hydrogenase of *Ralstonia eutropha* (Schäfer, 2014) and could be a possible clue to the extreme oxygen tolerance of group 1h/5 hydrogenases (Supplementary Figure S5).

The transcriptome analysis clearly showed that the Group 1d hydrogenase gene cluster was upregulated under oxygen-limited conditions consistent with higher hydrogenase activity and growth rates found at dO_2_ values below 0.2%. Previously, we showed that these two genes were also upregulated under the N_2_-fixing condition (Khadem *et al*, 2012a). In contrast, the other hydrogenase encoding gene cluster (Group 1h/5) and all accessory proteins were similarly expressed under oxygen-limited conditions (continuous culture) and under oxygen excess conditions (batch culture). This clearly correlates with the constant hydrogen uptake (activity) found in respiration experiments for cells grown under higher oxygen concentrations (above 0.2% O_2_). Therefore we may conclude that the observed growth with hydrogen found at these higher dO_2_ values is supported exclusively by the Group 1h/5 uptake hydrogenase.

Growth on hydrogen only sustained by a Group 1h/5 hydrogenase has not been reported before. Other microorganisms expressing Group 1h/5 hydrogenases activity co-metabolize hydrogen but have not been shown to grow on or benefit from this hydrogen consumption. Group 1h/5 hydrogenase activity has been reported for Actinobacteria like *Mycobacterium smegmatis* (Greening *et al*, 2014; Berney *et al*, 2014) (2.5 nmol.min^−1^.g DW^−1^; 10 μmol.min^−1^.g protein^−1^), and *Streptomyces* strains (Constant *et al*, 2010) (0.2–2 nmol.min^−1^.g DW^−1^), whereas Meredith *et al* (2014) reported extremely higher V_max_ values of 14–180 μmol.min^−1^.g protein^−1^ for *Streptomyces* species. ‘Knallgas' bacteria like *Ralstonia* sp. need the Group 1d hydrogenase for growth and express relatively low Group 1h/5 hydrogenase activity (Schäfer *et al*, 2013).

Although the Group 1h/5 hydrogenase of strain SolV was active up to at least ambient O_2_, growth on hydrogen was only observed below 1.5% O_2_. Clearly other factors also determine the oxygen tolerance during growth on hydrogen. The affinity for hydrogen of this enzyme (K_s_ 0.6 μM) was lower than those reported for Group 5 hydrogenases of *M. smegmatis* (50 nM) (Greening *et al*, 2014) and *Streptomyces* species (10 nM) (Constant *et al*, 2008); (40–400 nM) (Constant *et al*, 2010)). These Actinobacteria are held responsible for the high-affinity hydrogen uptake from the atmosphere (Constant *et al*, 2010; Meredith *et al*, 2014). The proteobacterial *R. eutropha* expresses only low Group 5 hydrogenase activity and showed a low affinity for hydrogen (K_m_ 3.6 μM, after purification) (Schäfer *et al*, 2013).

The hydrogen kinetics of the Group 1d hydrogenase of *M. fumariolicum* SolV, which is active only at low oxygen concentrations, could not be determined due to the constitutive expression of Group 1 h/5 hydrogenase. However, cells with both hydrogenases active exhibited an affinity constant of about 1 μM and we estimated that the apparent affinity constant of the Group 1d hydrogenase was higher than 1 μM but less than 2 μM, similar to that of group 1 containing proteobacterial ‘Knallgas' bacteria (Ludwig *et al*, 2009).

It should be realized that when using whole cells (or soils) to measure hydrogenase kinetics, only a so-called apparent affinity constant is obtained, which depends on the measured V_max_. This V_max_ is not necessarily the V_max_ of the hydrogenase enzyme but that of the overall reaction that may be limited by the follow-up steps being respiration and this may explain the large variations found in values (nM to low μM range) of closely related strains that contain the same hydrogenase (Greening *et al*, 2014). As a consequence the existence of a high affinity hydrogenase remains doubtful.

The mesophilic *Methylacidimicrobium* strains lack a Group 1h/5 hydrogenase and an O_2_-tolerant Group 1d hydrogenase. They only possess a hydrogenase closely related to group 1b hydrogenases. These are found mainly in microorganisms with an anoxic H_2_ respiration or photosynthesis, or supposed to be involved in protection against oxygen like in *Geobacter sulfurreducens* (Coppi *et al*, 2004). Thus we assumed that these mesophilic strains only could grow on hydrogen when oxygen is limited. Preliminary tests to grow *Methylacidimicrobium tartarophylax* 4AC on hydrogen showed that the hydrogenase is inactivated when the sensor reading dO_2_ value is above detection limit (0.01% oxygen) confirming the high sensitivity of this hydrogenase to oxygen.

The presence of an oxygen-insensitive (group 1h/5) hydrogenase is of great importance for hydrogen consumption in environments like oxic upper soil layers that have been reported to take up hydrogen from the atmosphere. Although growth of methanotrophic Verrucomicrobia on ppm level ambient hydrogen may not be likely, the simultaneous uptake of hydrogen at such low concentrations may occur while these bacteria grow on methane and possibly hydrogen produced in anoxic layers beneath. Perhaps more interestingly vice versa: reducing power of hydrogen may lower the threshold for oxidizing methane as described for methanol (Benstead *et al*, 1998; Jensen *et al*, 1998). This threshold has been suggested to be determined by the availability of reductant needed for the first step in methane oxidation. This step is performed by a methane monooxygenase and hydrogen has been shown to be a suitable donor of reducing equivalents (Hanczár *et al*, 2002). In the volcanic environment from where *M. fumariolicum* SolV was isolated, hydrogen concentrations in emitted gasses are much higher than those of methane (Chiodini *et al*, 2001) and this hydrogen might explain the uptake of methane even at atmospheric levels that was reported by Castaldi and Tedesco (2005). Greening and co-workers (Greening *et al*, 2016) showed that the occurrence of [NiFe] aerobic H_2_-uptake hydrogenases in the phylum Verrucomicrobia is more widespread, and suggests that Verrucomicrobia play a role in the hydrogen cycle not only in areas that emit geothermal gasses, but also in ecosystems such as rice paddy soil (van Passel *et al*, 2011), pasture soil (Kant *et al*, 2011) and the human gut (Derrien *et al*, 2004).

In conclusion, *M. fumariolicum* SolV is a real ‘Knallgas' bacterium but also able to consume methane and hydrogen simultaneously using an oxygen-sensitive and oxygen-tolerant hydrogenase, respectively. Detailed physiological studies and transcriptome analysis of the *Methylacidiphilum* strains that can grow on hydrogen is required to understand this interesting group of hydrogenases. Oxygen sensitivity of hydrogenases is a major drawback in application (Jugder *et al*, 2016). In view of the high oxygen tolerance of the Group 1 h/5hydrogenase from strain SolV, biochemical and biophysical studies of the purified hydrogenase are essential since this enzyme has a high potential in biotechnological applications.

## Figures and Tables

**Table 1:** The oxygen respiration profile of *Methylacidiphilum fumariolicum* SolV cells from different growth conditions using CH_4_ or H_2_

*Growth condition*	*e*^−^ *donor*	*Limitation*	*Growth rate (h*^−*1*^)^a^	*dO*_*2*_*%*	*O*_*2*_ *(μM)*^b^	*H*_*2*_ *respiration*^c^	*CH*_*4*_ *respiration*^c^
Batch	CH_4_	—	0.07	>10	—	15–20	280
Continuous	CH_4_	CH_4_	0.03	0.3–16	—	20–24	244–317
Continuous	CH_4_	O_2_	0.015	—	>30 5	12–17^d^ 42	232–293 n.d.
Batch	H_2_	—	0.047	0.02–0.04	< 5	66–71	29–39^e^
Batch	H_2_	—	0.01	1.5	1–100	20	17^e^
Continuous	H_2_	O_2_	0.04	0.01	100 5	17 29^f^	>171^g^ n.d.

a In batch and continuous cultures, growth rate refers to μ and D, respectively.

b The μM units refer to O_2_ concentrations in the liquid phase in the respiration chamber.

c The respiration rates are in nmol O_2_.min^−1^.mg DW^−1^.

d Pre-incubated at 10% O_2_ for 30 min.

e Batch grown after continuous growing for over a year on hydrogen gas only.

f Measured after exposure to 100 μM O_2_ in the above respiration experiment.

g After >20 generations. n.d.=not determined.

**Table 2:** The transcriptome profile of the genes involved in H_2_ metabolism and CO_2_ fixation of *Methylacidiphilum fumariolicum* SolV

*Protein annotation*	*Gene*	*Gene ID*^a^	*Expression level (RPKM)*^b^		
			*H*_*2*_ *culture*^c^	*CH*_*4*_ *culture*^d^	*Cells at μ*_*max*_^e^
Hydrogenase expression/formation protein hoxQ	*hox*Q	MfumV2_0886	269	112	157
Putative hydrogenase maturation protease	*hyc*L	MfumV2_0891	191	184	160
[NiFe] hydrogenase nickel insertion protein	*hyp*A	MfumV2_892	145	137	156
[NiFe] hydrogenase Group 1h/5 small subunit	*hhy*S	MfumV2_978	3449	1853	2076
[NiFe] hydrogenase Group 1h/5 large subunit	*hhy*L	MfumV2_979	2233	803	1746
Putative maturation protein	*hup*D	MfumV2_980	328	388	580
[NiFe] hydrogenase Ni-incorporation protein	*hha*B	MfumV2_988	613	543	480
High affinity Ni transporter		MfumV2_989	325	218	230
[NiFe] hydrogenase metallocenter assembly protein HhaF	*hha*F	MfumV2_990	77	43	92
[NiFe] hydrogenase metallocenter assembly protein HhaC	*hha*C	MfumV2_991	240	353	524
[NiFe] hydrogenase metallocenter assembly protein HhaD	*hha*D	MfumV2_992	727	483	948
[NiFe] hydrogenase metallocenter assembly protein HhaE	*hha*E	MfumV2_993	843	734	560
Hydrogenase expression protein HupH	*hup*H	MfumV2_1562	608	247	27
[NiFe] hydrogenase cytochrome b subunit	*hup*Z	MfumV2_1563	818	266	36
[NiFe] hydrogenase Group 1d large subunit	*hup*L	MfumV2_1564	1596	751	239
[NiFe] hydrogenase Group 1d small subunit	*hup*S	MfumV2_1565	3219	1370	47
RuBisCO^f^ small subunit	*cbb*S	Mfumv2_1495	6826	3456	8520
RuBisCO large subunit	*cbb*L	Mfumv2_1496	7576	4028	6419

a Available at the MicroScope annotation platform (https://www.genoscope.cns.fr/agc/microscope/home/).

b The mRNA expression is shown as RPKM according to Mortazavi *et al* (Saitou and Nei, 1987). Changes in expression in the continuous cultures (substrates H_2_ or CH_4_) compared to batch culture cells growing at μ_max_ are demonstrated by shading (up-regulation>twofold, dark grey, downregulation<0.5, light grey).

c Continuous culture using H_2_ as an electron donor.

d Continuous culture using CH_4_ as an electron donor.

e Batch culture using CH_4_ as an electron donor to obtain μ_max_=0.07 h^−1^.

f Ribulose-1,5-bisphosphate carboxylase/oxygenase.
