# Supplementary material for: Methylacidiphilum fumariolicum SolV, a thermoacidophilic ‘Knallgas' methanotroph with both an oxygen-sensitive and -insensitive hydrogenase
Source: ISME J. 2016 Dec 9;11(4):945–58. doi: 10.1038/ismej.2016.171 (PMC5364354; doi:10.1038/ismej.2016.171)
Supplement: Supplementary Material [file ismej2016171x1.pdf]

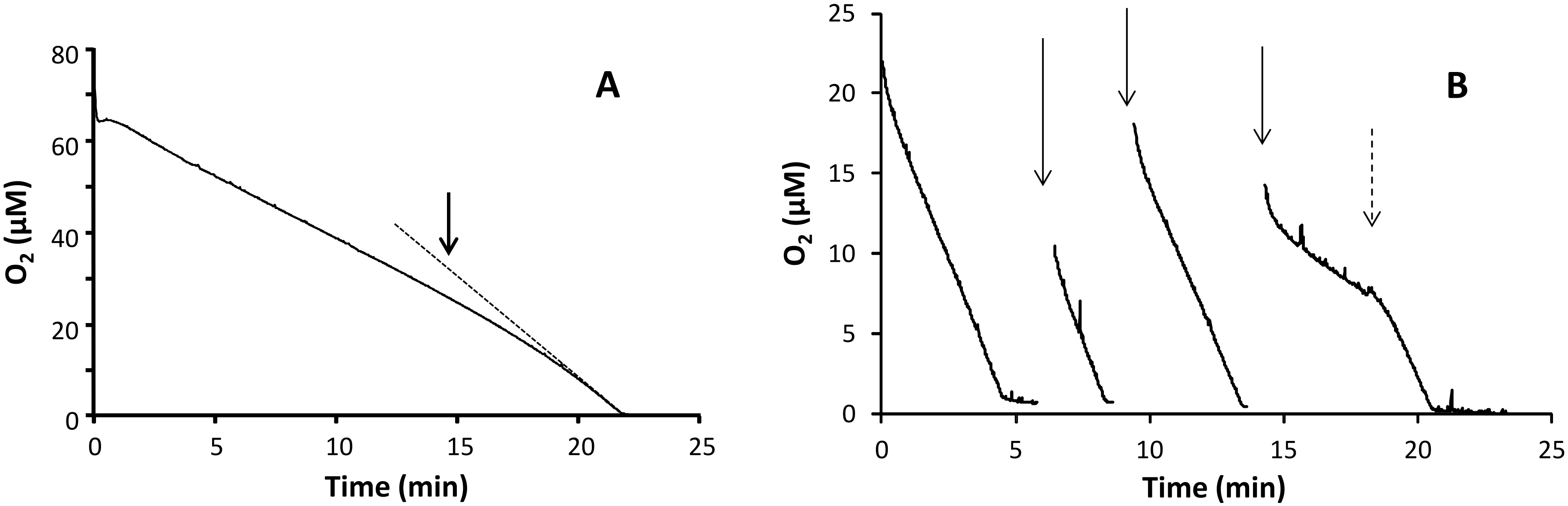

Supplementary Figure S1. (**A**) Oxygen consumption by strain SolV using a pre-incubated biomass sample at 10 % oxygen (and H_2) for 30 min. The arrow indicates where the respiration rate increased and the dashed line illustrates the increased rate. The respiration rate increased when the oxygen concentration was reaching low values (about 20 µM). This pointed to inhibition or reactivation after inactivation of an oxygen sensitive hydrogenase. When a sample of this culture was pre-incubated at an oxygen concentration of 10 % (in the presence of H_2) for 10 min, the subsequently measured respiration of hydrogen was rather constant (12 - 17 $nmol.min^{-1}.mg\ DW^{-1}$; Table 1) till O_2 concentrations were reaching values of about 30 µM after which the rates increased. The extent of reactivation was depending on the duration of oxygen exposure. Initial respiration rates were lower and the extent of reactivation was less upon longer oxygen exposure times.
(**B**) The respiration test with minimized oxygen exposure. Strain SolV cells were transferred to the respiration chamber with minimal O_2 exposure. The solid and dashed arrows show the replenishing of O_2 and H_2, respectively. When oxygen exposure was minimized and the respiration experiment was started at low oxygen concentrations, culture samples showed a much faster hydrogen respiration, but some reactivation still occurred. The highest rates (42 $nmol.min^{-1}.mg\ DW^{-1}$) were obtained when experiments started with 10 µM O_2 and rates were constant at this O_2 concentration also after twice replenishing the oxygen till 10 µM. More repetition resulted in inactivation.

Hydrogen respiration rates measured were at least twice the rate obtained for the continuous culture under methane limitation at dO_2 values ranging from 0.3 - 3.2 % oxygen (1.5 – 16 % air; discussed above). Therefore, it seems that under oxygen limitation another oxygen sensitive hydrogenase is expressed in addition to the constitutively expressed oxygen insensitive one.

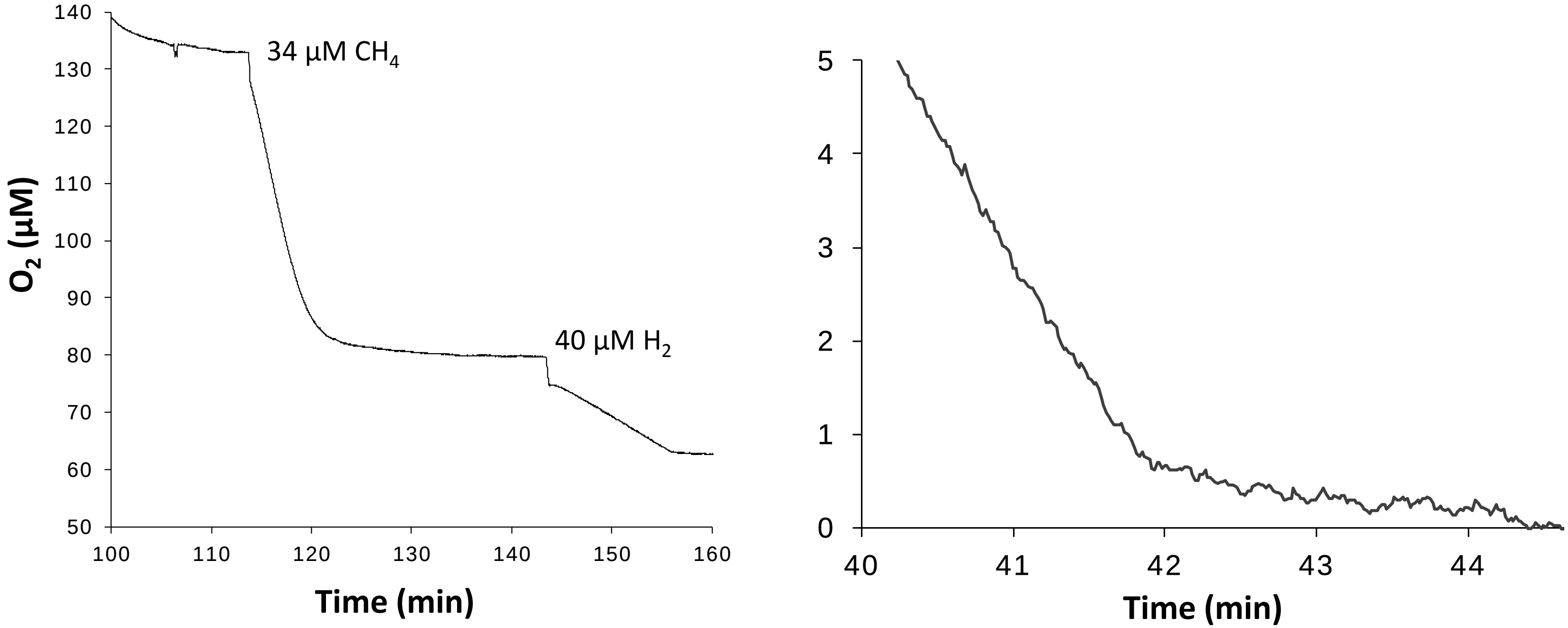

Supplementary Figure S2. Respiration tests to observe the affinity for hydrogen compared for methane. Hydrogen oxidation continues with a constant rate until it is almost completely finished (left). Assuming the full scale of oxygen consumed equivalent to the added 40 µM of hydrogen the rate is maximal down to well below 1 µM of hydrogen (right).

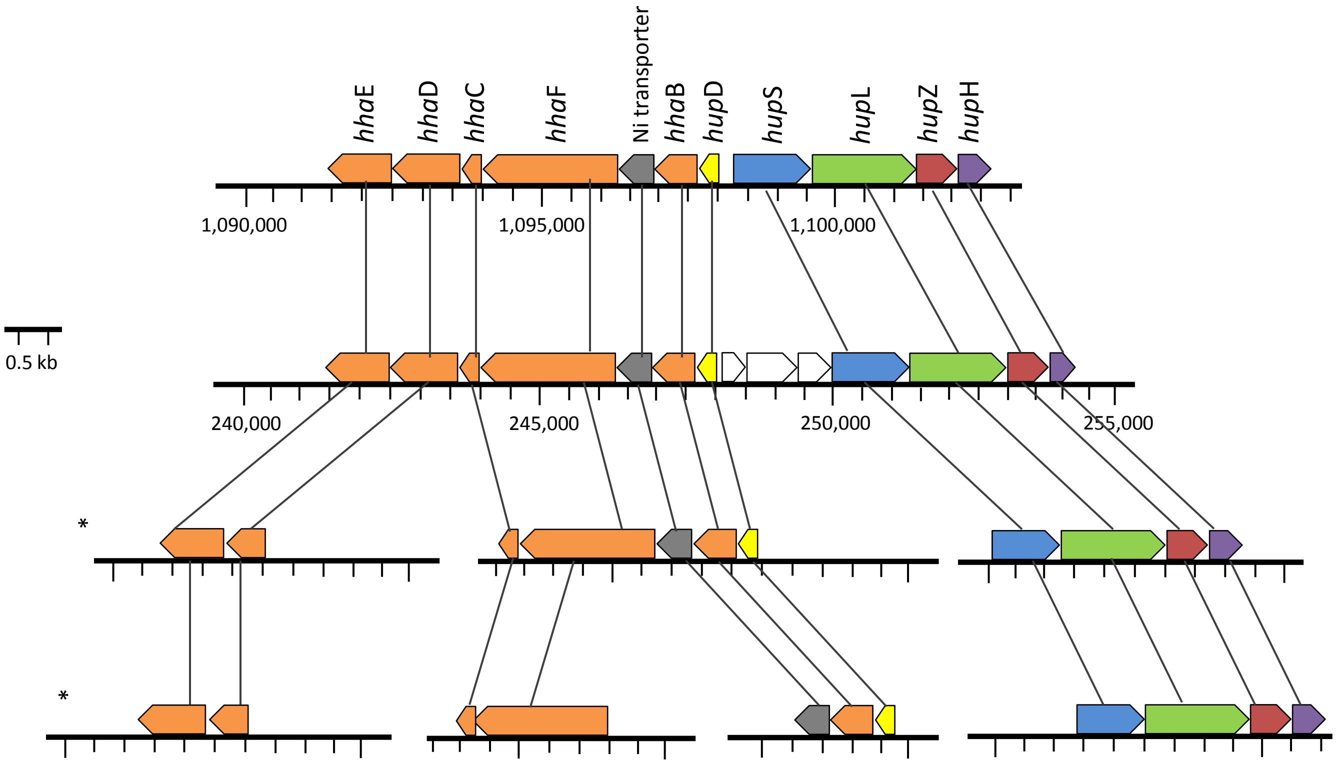

Supplementary Figure S3. Gene arrangement of the hydrogenases in the mesophilic strains LP2A, 3C, 4AC and 3B (top to bottom). Genes are color coded as follows: green = large subunit; blue = small subunit; yellow = putative hydrogenase maturation protease; orange = accessory proteins; red = b-type cytochrome subunit; purple = putative expression/formation protein; gray = Nickel transporter; dark blue = nickel insertion protein. The draft genomes of strains 4AC and 3B are fragmented (* = 4AC and 3B contigs).

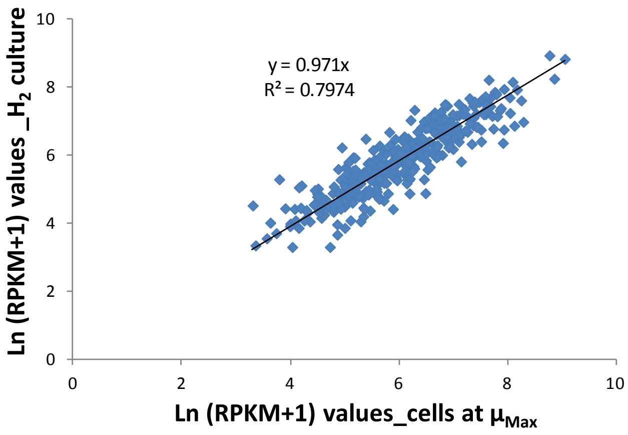

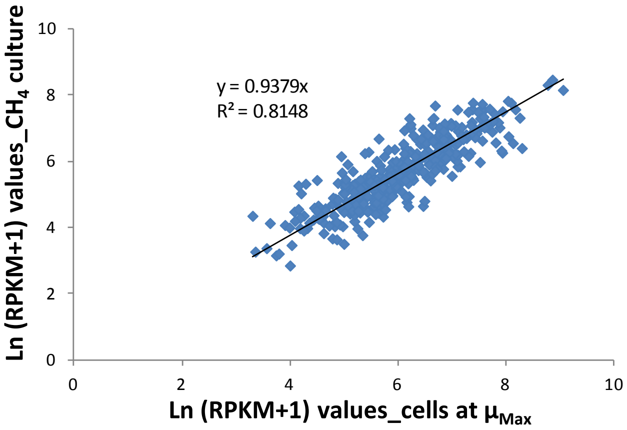

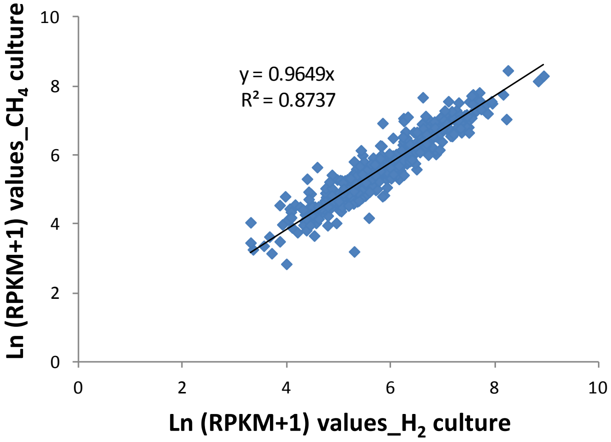

Supplementary Figure S4. Plots of Ln (RPKM + 1) values of 393 housekeeping genes (in total 442.8 kbp) involved in energy generation, ribosome assembly, carbon fixation (CBB cycle), C1 metabolism (except for *pmo*), amino acid synthesis, cell wall synthesis, translation, transcription, DNA replication, and tRNA synthesis.

1. *Methylacidimicrobium fagopyrum* 3C
2. *Methylacidimicrobium* sp. LP2A
3. *Methylacidiphilum fumariolicum* SolV *hup*-type (CCG92215)
4. *Methylacidiphilum kamchatkense* Kam1 (KIE59448)
5. *Methylacidiphilum infernorum* V4 (YP_001939972)
6. *Ralstonia eutropha* (P31891)
7. *Methylacidimicrobium tartarophylax* 4AC (peg.187)
8. *Methylacidimicrobium cyclopophantes* 3B (peg.2244)
9. *Ralstonia eutropha* (WP_011153987)
10. *Methylacidiphilum fumariolicum* SolV *hhy*-type (CCG92937)
11. *Methylacidiphilum kamchatkense* Kam1 (KIE58221)

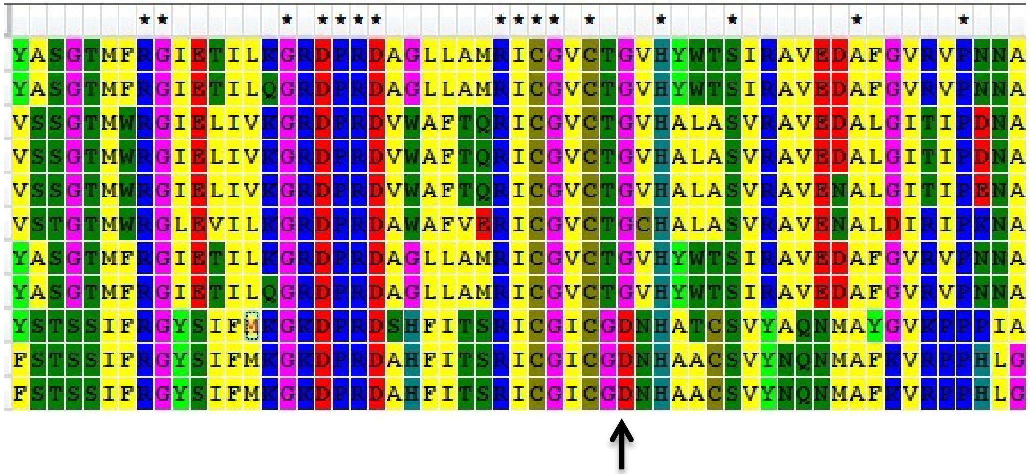

Supplementary Figure S5. The conserved Asp[22] present in the Group 1h/5 hydrogenase (arrow) is the fourth position for a 4Fe4S cluster. The sequences number 9, 10 and 11 are the Group 1h/5 hydrogenases (*hhy*-type) of *Ralstonia eutropha*, *M. acidiphilum* SolV and *M. kamchatkense* Kam1, respectively. The other sequences represent the putative oxygen-sensitive Group 1d hydrogenases (*hup*-type).

Supplementary Table S1. An overview of the [NiFe] uptake hydrogenases present in Verrucomicrobial thermophilic and mesophilic strains isolated from geothermal environments.

Organism	Gene name	Gene ID [a]	Hydrogenase Group
Methylacidiphilum fumariolicum SolV	*hup*L [b]	MfumV2_1564	Group 1d
	*hup*S [c]	MfumV2_1565	Group 1d
	*hhy*L	MfumV2_0979	Group 1h/5
	*hhy*S	MfumV2_0978	Group 1h/5
Methylacidiphilum kamchatkensis Kam1	*hup*L	JQNX01_v1_10368	Group 1d
	*hup*S	JQNX01_v1_10367	Group 1d
	*hhy*L	JQNX01_v1_60118	Group 1h/5
	*hhy*S	JQNX01_v1_60119	Group 1h/5
Methylacidiphilum infernorum V4	*hup*L	Minf_1320	Group 1d
	*hup*S	Minf_1321	Group 1d
Methylacidimicrobium tartarophylax 4AC [d]	*hup*L	60380.peg.187 [e]	Group 1b
	*hup*S	60380.peg.186	Group 1b
Methylacidimicrobium fagopyrum 3C [d]	*hup*L	VER3v2_90073 [f]	Group 1b
		VER3v2_90074	Group 1b
	*hup*S	VER3v2_90072	Group 1b
Methylacidimicrobium cyclopophantes 3B [d]	*hup*L	60379.peg.2444 [e]	Group 1b
	*hup*S	60379.peg.2445	Group 1b
Methylacidimicrobium strain LP2A [d]	*hup*L	MAMLP_v1_11153	Group 1b
	*hup*S	MAMLP_v1_11152	Group 1b

[a] Available at the MicroScope annotation platform.
[b] Large subunit.
[c] Small subunit.
[d] Mesophilic strains are closely related to Group 1b hydrogenases.
[e] Strains 4AC and 3B genomes are fragmented.
[f] The large subunit of hydrogenase of strain 3C is present in two pieces.

Supplementary Table S2. An overview of the [NiFe] uptake hydrogenases and all proteins involved in H_2 metabolism present in Verrucomicrobial thermophilic strains isolated in the geothermal environments.

**Gene name**	**Gene ID** [a]			**Protein annotation**
	**Strain SolV**	**Strain Kam1**	**Strain V4**	
*hox*Q	MfumV2_0886	JQNX01_v1_30026	Minf_1617	Hydrogenase expression/formation protein hoxQ
*hyc*I	MfumV2_0891	JQNX01_v1_30021	Minf_1611	Putative hydrogenase maturation protease
*hyp*A	MfumV2_0892	JQNX01_v1_30020	Minf_1610	[NiFe] hydrogenase nickel insertion protein
*hhy*S	MfumV2_0978	JQNX01_v1_60119	n. p. [b]	[NiFe] hydrogenase Group 1h/5 small subunit
*hhy*L	MfumV2_0979	JQNX01_v1_60118	n. p.	[NiFe] hydrogenase Group 1h/5 large subunit
*hup*D	MfumV2_0980	JQNX01_v1_60117	n. p.	Putative maturation protein
*hha*B	MfumV2_0988	JQNX01_v1_60109	Minf_1799	[NiFe] hydrogenase nickel incorporation-associated protein
	MfumV2_0989	JQNX01_v1_60107	Minf_1798	High-affinity nickel transporter
*hha*F	MfumV2_0990	JQNX01_v1_60106	Minf_1797	[NiFe] hydrogenase metallocenter assembly protein hhaF
*hha*C	MfumV2_0991	JQNX01_v1_60105	Minf_1796	[NiFe] hydrogenase metallocenter assembly protein hhaC
*hha*D	MfumV2_0992	JQNX01_v1_60104	Minf_1795	[NiFe] hydrogenase metallocenter assembly protein hhaD
*hha*E	MfumV2_0993	JQNX01_v1_60103	Minf_1794	[NiFe] hydrogenase metallocenter assembly protein hhaE
*hup*H	MfumV2_1562	JQNX01_v1_10370	Minf_1318	HupH hydrogenase expression protein
*hup*Z	MfumV2_1563	JQNX01_v1_10369	Minf_1319	[NiFe] hydrogenase cytochrome b subunit
*hup*L	MfumV2_1564	JQNX01_v1_10368	Minf_1320	[NiFe] hydrogenase Group 1d large subunit
*hup*S	MfumV2_1565	JQNX01_v1_10367	Minf_1321	[NiFe] hydrogenase Group 1d small subunit

[a] available at the MicroScope annotation platform (https://www.genoscope.cns.fr/agc/microscope/home/).
[b] not present.

Supplementary Table S3. An overview of the [NiFe] uptake hydrogenases and all proteins involved in H_2 metabolism present in Verrucomicrobial mesophilic strains isolated in the geothermal environments.

Gene name	Gene ID [a]				Protein annotation
	Strain LP2A	Strain 3C	Strain 3B [b]	Strain 4AC [b]	
*hup*D	MAMLP_v1_11150	VER3v2_90067	60379.peg.727	60380.peg.2344	Putative maturation protein
*hha*B	MAMLP_v1_11149	VER3v2_90065	60379.peg.728	60380.peg.2345	[NiFe] hydrogenase nickel incorporation-associated protein
	MAMLP_v1_11148	VER3v2_90064	60379.peg.729	60380.peg.2346	High-affinity nickel transporter
*hha*F	MAMLP_v1_11147	VER3v2_90063	60379.peg.2260	60380.peg.2347	[NiFe] hydrogenase metallocenter assembly protein hhaF
*hha*C	MAMLP_v1_11146	VER3v2_90062	60379.peg.2261	60380.peg.2348	[NiFe] hydrogenase metallocenter assembly protein hhaC
*hha*D	MAMLP_v1_11145	VER3v2_90061	60379.peg.981	60380.peg.2393	[NiFe] hydrogenase metallocenter assembly protein hhaD
*hha*E	MAMLP_v1_11144	VER3v2_90060	60379.peg.982	60380.peg.2392	[NiFe] hydrogenase metallocenter assembly protein hhaE
*hup*H	MAMLP_v1_11155	VER3v2_100005	60379.peg.2442	60380.peg.189	HupH hydrogenase expression protein
*hup*Z	MAMLP_v1_11154	VER3v2_100004	60379.peg.2443	60380.peg.188	[NiFe] hydrogenase cytochrome b subunit
*hup*L	MAMLP_v1_11153	VER3v2_90073 [c]<br>VER3v2_90074	60379.peg.2444	60380.peg.187	[NiFe] hydrogenase large subunit
*hup*S	MAMLP_v1_11152	VER3v2_90072	60379.peg.2445	60380.peg.186	[NiFe] hydrogenase small subunit

[a] available at the MicroScope annotation platform (https://www.genoscope.cns.fr/agc/microscope/home/).
[b] Strains 4AC and 3B genomes are fragmented.
[c] The large subunit of hydrogenase of strain 3C is present in two pieces.

Supplementary Table S4 | RNA-Seq analysis of *Methylacidiphilum fumariolicum* SolV grown under different conditions

		CH_4/NO_3^- bioreactor			H_2/NH_4^+ bioreactor			Cells at µMax			
Name	Gene length	RPKM	Unique gene reads	Total gene reads	RPKM	Unique gene reads	Total gene reads	RPKM	Unique gene reads	Total gene reads	Annotation
Mfumv2_0001	1356	243	238	238	223	201	201	216	290	291	Chromosomal replication initiator protein DnaA
Mfumv2_0002	1116	262	209	211	287	213	213	333	366	368	DNA polymerase III beta subunit (EC 2.7.7.7)
Mfumv2_0003	387	129	36	36	252	65	65	261	100	100	hypothetical protein
Mfumv2_0004	153	45	5	5	69	7	7	112	17	17	hypothetical protein
Mfumv2_0005	213	52	8	8	78	11	11	128	27	27	Lipoprotein signal peptidase (EC 3.4.23.36)
Mfumv2_0006	927	143	95	96	360	222	222	388	357	357	Quinolinate synthetase (EC 2.5.1.72)
Mfumv2_0007	609	298	130	131	207	84	84	250	150	151	hypothetical protein
Mfumv2_0008	1101	78	62	62	82	60	60	121	132	132	ATP-dependent DNA ligase
Mfumv2_0009	1290	96	89	89	126	108	108	285	365	365	UDP-glucose dehydrogenase (EC 1.1.1.22)
Mfumv2_0010	1269	256	235	235	291	246	246	289	363	364	Adenylosuccinate synthetase (EC 6.3.4.4)
Mfumv2_0011	795	284	163	163	410	217	217	251	197	198	Undecaprenyl diphosphate synthase (EC 2.5.1.31)
Mfumv2_0012	879	148	94	94	155	90	91	150	131	131	Phosphatidate cytidylyltransferase (EC 2.7.7.41)
Mfumv2_0013	648	154	71	72	199	86	86	182	117	117	Phosphatidylserine decarboxylase (EC 4.1.1.65)
Mfumv2_0014	918	281	186	186	379	232	232	249	227	227	CDP-diacylglycerol--serine O-phosphatidyltransferase (EC 2.7.8.8)
Mfumv2_0015	1182	396	338	338	405	319	319	891	1042	1044	Lysine 2,3-aminomutase (EC 5.4.3.2)
Mfumv2_0016	1194	299	258	258	375	298	298	475	561	562	LSU m5C1962 methyltransferase RlmI
Mfumv2_0017	2439	187	328	329	413	671	671	296	711	717	Phosphoenolpyruvate synthase (EC 2.7.9.2)
Mfumv2_0018	2742	132	259	261	231	421	422	189	515	515	Acetyl-CoA synthetase (ADP-forming) alpha and beta chains, putative
Mfumv2_0019	780	1111	626	626	1174	609	610	658	508	509	Chromosome (plasmid) partitioning protein ParB / Stage 0 sporulation protein J
Mfumv2_0021	1803	198	256	258	142	171	171	178	319	319	DNA mismatch repair protein MutL
Mfumv2_0022	1101	252	200	200	314	230	230	225	246	246	GTP-binding and nucleic acid-binding protein YchF
Mfumv2_0023	552	53	21	21	60	22	22	91	50	50	Transcriptional activator RfaH
Mfumv2_0024	906	248	162	162	431	260	260	421	378	378	dTDP-4-dehydrorhamnose reductase (EC 1.1.1.133)
Mfumv2_0025	1242	246	221	221	340	281	281	391	482	482	Pleiotropic regulatory protein
Mfumv2_0026	2508	219	394	396	265	440	443	218	543	543	organic solvent tolerance protein
Mfumv2_0027	594	100	42	43	61	24	24	604	355	356	hypothetical protein
Mfumv2_0028	585	109	45	46	72	28	28	100	58	58	Potassium-transporting ATPase C chain (EC 3.6.3.12) (TC 3.A.3.7.1)
Mfumv2_0029	1941	136	190	190	94	122	122	74	142	142	Potassium-transporting ATPase B chain (EC 3.6.3.12) (TC 3.A.3.7.1)
Mfumv2_0030	180	0	0	0	0	0	0	22	4	4	hypothetical protein
Mfumv2_0031	1719	126	156	156	63	72	72	80	136	136	Potassium-transporting ATPase A chain (EC 3.6.3.12) (TC 3.A.3.7.1)
Mfumv2_0032	2295	45	75	75	52	80	80	85	194	194	Helicase PriA essential for oriC/DnaA-independent DNA replication
Mfumv2_0033	210	1029	156	156	987	138	138	408	85	85	Dodecin (COG3360) Flavin-binding
Mfumv2_0034	483	312	109	109	274	88	88	288	138	138	Guanine deaminase (EC 3.5.4.3)
Mfumv2_0035	132	0	0	0	34	3	3	23	3	3	hypothetical protein
Mfumv2_0036	675	328	160	160	305	137	137	178	119	119	Nitric-oxide reductase subunit C (EC 1.7.99.7)
Mfumv2_0037	1422	77	79	79	150	142	142	83	117	117	Nitric-oxide reductase subunit B (EC 1.7.99.7)
Mfumv2_0038	321	82	19	19	89	18	19	41	13	13	hypothetical protein
Mfumv2_0039	519	430	161	161	417	144	144	303	156	156	hypothetical protein
Mfumv2_0040	171	219	27	27	123	14	14	271	45	46	hypothetical protein
Mfumv2_0043	639	412	190	190	442	187	188	287	182	182	Superoxide dismutase [Mn] ; Superoxide dismutase [Fe] (EC 1.15.1.1); Superoxide dismutase ChrC
Mfumv2_0044	1704	138	170	170	229	260	260	273	461	462	DNA repair protein RecN
Mfumv2_0045	1014	288	211	211	497	336	336	749	752	753	Peptide chain release factor 2; programmed frameshift-containing
Mfumv2_0046	651	49	23	23	62	27	27	73	47	47	hypothetical protein
Mfumv2_0047	474	225	77	77	304	96	96	777	364	365	Molybdenum cofactor biosynthesis protein MoaC

Mfumv2_0048	504	93	34	34	173	57	58	368	184	184	Molybdopterin biosynthesis Mog protein, molybdochelatase
Mfumv2_0049	429	310	96	96	504	144	144	519	221	221	Phosphoribosyl-AMP cyclohydrolase (EC 3.5.4.19)
Mfumv2_0050	1080	115	90	90	168	121	121	191	205	205	Acyl-CoA reductase (EC 1.2.1.50)
Mfumv2_0051	1095	91	72	72	97	71	71	114	124	124	Possible acyl protein synthase/acyl-CoA reductase-like protein
Mfumv2_0052	180	392	51	51	525	63	63	207	37	37	Membrane protein CcmA involved in cell shape determination
Mfumv2_0053	765	154	85	85	288	147	147	154	117	117	Zn-finger protein
Mfumv2_0055	147	565	60	60	929	91	91	563	82	82	hypothetical protein
Mfumv2_0056	135	21	2	2	44	4	4	239	32	32	hypothetical protein
Mfumv2_0057	135	82	8	8	22	2	2	90	12	12	hypothetical protein
Mfumv2_0058	213	7	1	1	0	0	0	9	2	2	hypothetical protein
Mfumv2_0059	123	11	1	1	12	1	1	8	1	1	hypothetical protein
Mfumv2_0060	2496	372	669	670	355	589	590	284	704	704	CRISPR-associated HD domain protein
Mfumv2_0061	780	185	104	104	423	220	220	278	215	215	hypothetical protein
Mfumv2_0062	768	146	81	81	260	133	133	160	122	122	hypothetical protein
Mfumv2_0063	210	40	6	6	286	40	40	120	25	25	hypothetical protein
Mfumv2_0064	1359	171	168	168	180	163	163	128	173	173	hypothetical protein
Mfumv2_0065	117	402	34	34	449	35	35	448	52	52	hypothetical protein
Mfumv2_0066	360	169	44	44	92	22	22	168	60	60	hypothetical protein
Mfumv2_0069	153	18	2	2	0	0	0	26	4	4	hypothetical protein
Mfumv2_0070	180	54	7	7	42	5	5	275	49	49	hypothetical protein
Mfumv2_0071	894	48	31	31	34	20	20	194	172	172	hypothetical protein
Mfumv2_0072	690	110	55	55	98	45	45	238	163	163	Beta-phosphoglucomutase (EC 5.4.2.6)
Mfumv2_0073	1242	21	19	19	28	23	23	47	58	58	hypothetical protein
Mfumv2_0074	822	645	383	383	742	406	406	475	387	387	GTP cyclohydrolase I (EC 3.5.4.16) type 2
Mfumv2_0075	1923	202	280	281	256	328	328	690	1313	1315	Thiamin biosynthesis protein ThiC
Mfumv2_0076	2769	337	670	673	353	650	651	917	2518	2519	2-oxoglutarate dehydrogenase E1 component (EC 1.2.4.2)
Mfumv2_0077	1167	409	343	345	441	343	343	913	1056	1057	Dihydrolipoamide succinyltransferase (E2) of 2-oxoglutarate dehydrogenase complex (EC 2.3.1.61)
Mfumv2_0078	1401	196	198	198	243	227	227	520	721	722	Dihydrolipoamide dehydrogenase of 2-oxoglutarate dehydrogenase (EC 1.8.1.4)
Mfumv2_0079	1170	135	114	114	139	107	108	254	293	295	Alanine racemase (EC 5.1.1.1)
Mfumv2_0080	576	41	17	17	112	43	43	98	56	56	Inactive homolog of metal-dependent proteases, putative molecular chaperone
Mfumv2_0081	426	46	14	14	74	21	21	130	55	55	ATPase YjeE, predicted to have essential role in cell wall biosynthesis
Mfumv2_0082	948	91	62	62	97	61	61	191	180	180	Thiamine-monophosphate kinase (EC 2.7.4.16)
Mfumv2_0083	780	73	41	41	112	58	58	114	88	88	Abortive infection protein
Mfumv2_0084	993	282	202	202	340	225	225	376	370	370	signal peptide peptidase SppA, 36K type
Mfumv2_0085	1530	162	179	179	215	219	219	260	394	394	Methionyl-tRNA synthetase (EC 6.1.1.10)
Mfumv2_0086	339	784	190	192	833	187	188	881	296	296	Integration host factor alpha/beta
Mfumv2_0087	1218	249	219	219	264	214	214	681	821	822	Histidyl-tRNA synthetase (EC 6.1.1.21)
Mfumv2_0088	1824	527	692	694	336	408	408	486	878	879	Aspartyl-tRNA synthetase (EC 6.1.1.12) @ Aspartyl-tRNA(Asn) synthetase (EC 6.1.1.23)
Mfumv2_0089	1431	345	357	357	336	320	320	433	613	614	Pyruvate kinase (EC 2.7.1.40)
Mfumv2_0090	852	78	48	48	74	42	42	212	179	179	Short-chain dehydrogenase/reductase SDR
Mfumv2_0091	744	739	396	397	751	372	372	1277	942	942	hypothetical protein
Mfumv2_0092	570	486	200	200	540	205	205	925	522	523	Deoxycytidine triphosphate deaminase (EC 3.5.4.13)
Mfumv2_0093	1089	342	269	269	392	284	284	632	682	683	Ribosomal RNA large subunit methyltransferase N (EC 2.1.1.-)
Mfumv2_0094	345	437	109	109	648	149	149	1137	389	389	hypothetical protein
Mfumv2_0096	1368	58	57	57	48	44	44	50	67	68	hypothetical protein
Mfumv2_0100	198	2224	317	318	1752	229	231	632	122	124	hypothetical protein
Mfumv2_0101	144	29	1	3	0	0	0	49	5	7	hypothetical protein
Mfumv2_0102	363	27	5	7	41	8	10	17	6	6	hypothetical protein
Mfumv2_0103	471	88	30	30	77	24	24	32	14	15	hypothetical protein
Mfumv2_0104	1047	140	106	106	222	155	155	90	93	93	DUF1432 domain-containing protein

Mfumv2_0105	483	92	32	32	230	74	74	192	92	92	hypothetical protein
Mfumv2_0106	1416	62	63	63	68	64	64	115	162	162	Probable Co/Zn/Cd efflux system membrane fusion protein
Mfumv2_0107	3210	87	201	201	99	212	212	79	253	253	Cobalt-zinc-cadmium resistance protein CzcA; Cation efflux system protein CusA
Mfumv2_0108	132	84	8	8	23	2	2	46	6	6	hypothetical protein
Mfumv2_0109	309	1165	260	260	1467	302	302	555	170	170	transmembrane protein
Mfumv2_0111	159	122	14	14	76	8	8	108	17	17	hypothetical protein
Mfumv2_0112	669	1159	559	560	1064	473	474	1253	830	831	Ferric siderophore transport system, biopolymer transport protein ExbB
Mfumv2_0113	417	442	133	133	727	202	202	774	320	320	Biopolymer transport protein ExbD/TolR
Mfumv2_0114	162	60	7	7	167	18	18	93	15	15	hypothetical protein
Mfumv2_0115	366	87	23	23	82	20	20	58	21	21	ATP-dependent DNA ligase (EC 6.5.1.1) clustered with Ku protein, LigD
Mfumv2_0116	579	407	170	170	449	172	173	186	107	107	ADP-ribose pyrophosphatase (EC 3.6.1.13)
Mfumv2_0117	357	97	25	25	88	21	21	192	68	68	Large-conductance mechanosensitive channel
Mfumv2_0118	591	108	46	46	191	75	75	143	84	84	hypothetical protein
Mfumv2_0119	759	117	64	64	188	95	95	187	141	141	Inositol-1-monophosphatase (EC 3.1.3.25)
Mfumv2_0120	1551	85	93	95	113	115	117	198	301	304	hypothetical protein
Mfumv2_0121	657	228	107	108	187	82	82	290	189	189	thiol methyltransferase 1-like
Mfumv2_0122	717	214	111	111	314	149	150	406	289	289	Phosphoribosylformylglycinamidine synthase, glutamine amidotransferase subunit (EC 6.3.5.3)
Mfumv2_0124	1332	390	375	375	423	375	375	360	476	476	Adenylosuccinate lyase (EC 4.3.2.2)
Mfumv2_0125	600	1068	462	463	738	295	295	1225	729	729	2'-5' RNA ligase
Mfumv2_0126	123	0	0	0	24	2	2	66	8	8	hypothetical protein
Mfumv2_0127	384	306	85	85	254	64	65	263	100	100	NADH ubiquinone oxidoreductase chain A (EC 1.6.5.3)
Mfumv2_0128	1770	549	701	702	715	843	843	590	1036	1036	Acetolactate synthase large subunit (EC 2.2.1.6)
Mfumv2_0129	348	366	91	92	380	88	88	519	179	179	Possible ATLS1-like light-inducible protein
Mfumv2_0130	432	695	217	217	956	275	275	3364	1441	1441	LSU ribosomal protein L13p (L13Ae)
Mfumv2_0131	405	663	194	194	1676	452	452	3772	1515	1515	SSU ribosomal protein S9p (S16e)
Mfumv2_0132	1038	344	258	258	334	230	231	1261	1297	1298	N-acetyl-gamma-glutamyl-phosphate reductase (EC 1.2.1.38)
Mfumv2_0133	1215	471	413	413	711	574	575	965	1163	1163	Glutamate N-acetyltransferase (EC 2.3.1.35) / N-acetylglutamate synthase (EC 2.3.1.1)
Mfumv2_0134	891	463	298	298	497	295	295	790	698	698	Acetylglutamate kinase (EC 2.7.2.8)
Mfumv2_0135	1224	282	249	249	212	172	173	316	384	384	Acetylornithine aminotransferase (EC 2.6.1.11)
Mfumv2_0136	918	196	130	130	213	130	130	239	218	218	Ornithine carbamoyltransferase (EC 2.1.3.3)
Mfumv2_0137	138	0	0	0	22	2	2	37	5	5	hypothetical protein
Mfumv2_0138	1266	84	77	77	138	116	116	88	111	111	NADH dehydrogenase (EC 1.6.99.3)
Mfumv2_0139	717	93	48	48	119	57	57	149	106	106	cyclase family protein
Mfumv2_0140	1665	195	235	235	255	283	283	172	284	284	Glycogen synthase, ADP-glucose transglucosylase (EC 2.4.1.21)
Mfumv2_0141	615	171	76	76	271	111	111	162	99	99	hypothetical protein
Mfumv2_0142	1041	978	734	735	699	485	485	301	311	311	fatty acid desaturase
Mfumv2_0143	795	207	119	119	170	90	90	157	124	124	Thymidylate synthase (EC 2.1.1.45)
Mfumv2_0144	2514	56	101	101	94	158	158	123	307	307	DNA mismatch repair protein MutS
Mfumv2_0145	357	450	116	116	383	91	91	226	80	80	COG2363
Mfumv2_0146	2607	758	1425	1427	1128	1959	1959	786	2030	2032	ClpB protein
Mfumv2_0147	378	623	170	170	596	149	150	739	275	277	hypothetical protein
Mfumv2_0148	1014	164	120	120	210	142	142	363	364	365	Exopolyphosphatase (EC 3.6.1.11)
Mfumv2_0149	1314	71	67	67	151	132	132	329	429	429	Glutamate decarboxylase (EC 4.1.1.15)
Mfumv2_0150	1677	36	44	44	37	41	41	79	132	132	hypothetical protein
Mfumv2_0151	912	296	194	195	374	224	227	606	547	548	hypothetical protein
Mfumv2_0153	165	8	1	1	0	0	0	6	1	1	hypothetical protein
Mfumv2_0154	219	57	9	9	89	13	13	55	12	12	hypothetical protein
Mfumv2_0155	138	30	3	3	120	11	11	15	2	2	hypothetical protein
Mfumv2_0156	768	23	13	13	18	9	9	29	22	22	ABC transporter related
Mfumv2_0157	1011	37	27	27	22	15	15	54	54	54	transport system permease protein

Mfumv2_0158	1038	2446	1831	1834	359	248	248	331	341	341	hypothetical protein
Mfumv2_0159	1140	15	12	12	12	9	9	12	14	14	periplasmic binding protein
Mfumv2_0160	2865	42	86	86	14	27	27	21	60	60	Outer membrane receptor for ferric coprogen and ferric-rhodotorulic acid
Mfumv2_0161	213	20	3	3	56	8	8	33	7	7	hypothetical protein
Mfumv2_0163	234	112	19	19	160	25	25	181	42	42	hypothetical protein
Mfumv2_0164	126	22	2	2	12	1	1	8	1	1	hypothetical protein
Mfumv2_0165	201	83	12	12	67	9	9	60	12	12	hypothetical protein
Mfumv2_0166	168	288	35	35	384	43	43	174	29	29	hypothetical protein
Mfumv2_0167	678	449	219	220	255	115	115	184	124	124	Soluble lytic murein transglycosylase precursor (EC 3.2.1.-)
Mfumv2_0168	891	45	29	29	54	32	32	65	57	57	ribonuclease BN
Mfumv2_0169	474	961	328	329	1308	413	413	345	162	162	hypothetical protein
Mfumv2_0170	2463	50	89	89	28	45	46	45	110	110	TonB-dependent receptor
Mfumv2_0171	423	23	7	7	28	8	8	38	15	16	hypothetical protein
Mfumv2_0172	1326	69	65	66	119	105	105	95	125	125	GTPase and tRNA-U34 5-formylation enzyme TrmE
Mfumv2_0173	1029	113	84	84	175	120	120	172	176	176	Dihydroorotate dehydrogenase (EC 1.3.3.1)
Mfumv2_0174	681	209	103	103	117	53	53	210	142	142	metallo-beta-lactamase domain protein
Mfumv2_0175	2448	75	133	133	44	68	71	60	145	145	Outer membrane receptor protein, mostly Fe transport
Mfumv2_0176	1095	33	26	26	55	40	40	87	92	94	RNA methyltransferase, TrmA family
Mfumv2_0177	612	41	18	18	64	26	26	107	65	65	hypothetical protein
Mfumv2_0178	138	50	5	5	65	6	6	80	11	11	hypothetical protein
Mfumv2_0179	225	18	3	3	13	2	2	22	5	5	hypothetical protein
Mfumv2_0180	273	15	3	3	11	2	2	22	6	6	hypothetical protein
Mfumv2_0181	126	0	0	0	12	1	1	8	1	1	hypothetical protein
Mfumv2_0182	606	73	32	32	30	11	12	68	41	41	hypothetical protein
Mfumv2_0183	1125	70	57	57	113	85	85	97	108	108	Inner membrane protein
Mfumv2_0184	135	82	7	8	33	3	3	37	5	5	hypothetical protein
Mfumv2_0185	126	626	55	57	60	5	5	48	6	6	hypothetical protein
Mfumv2_0186	1722	84	105	105	134	154	154	67	114	114	Oxidoreductase, molybdopterin-binding
Mfumv2_0187	411	98	29	29	208	57	57	71	29	29	hypothetical protein
Mfumv2_0188	576	96	40	40	107	41	41	140	80	80	Predicted phosphatase homologous to the C-terminal domain of histone macroH2A1
Mfumv2_0190	453	199	62	65	192	58	58	93	42	42	Methyltransferase type 11
Mfumv2_0191	171	40	5	5	44	5	5	24	4	4	hypothetical protein
Mfumv2_0192	201	34	5	5	7	1	1	50	10	10	hypothetical protein
Mfumv2_0193	306	3326	735	735	5897	1201	1202	2953	896	896	DNA-binding protein HU-beta
Mfumv2_0194	1332	172	165	165	228	202	202	222	293	293	tRNA nucleotidyltransferase (EC 2.7.7.21) (EC 2.7.7.25)
Mfumv2_0195	1008	71	52	52	42	28	28	68	68	68	hypothetical protein
Mfumv2_0196	2835	270	549	552	206	389	389	381	1068	1070	Glycine dehydrogenase [decarboxylating] (glycine cleavage system P protein) (EC 1.4.4.2)
Mfumv2_0197	342	547	135	135	505	115	115	501	170	170	Glycine cleavage system H protein
Mfumv2_0198	1026	393	290	291	496	339	339	278	283	283	Aminomethyltransferase (glycine cleavage system T protein) (EC 2.1.2.10)
Mfumv2_0199	624	244	110	110	1140	474	474	1156	715	715	hypothetical protein
Mfumv2_0200	177	63	8	8	305	36	36	427	75	75	hypothetical protein
Mfumv2_0201	1599	556	642	642	547	583	583	665	1055	1055	Type I restriction-modification system, DNA-methyltransferase subunit M (EC 2.1.1.72)
Mfumv2_0202	1305	207	195	195	216	188	188	262	339	339	Type I restriction-modification system, specificity subunit S (EC 3.1.21.3)
Mfumv2_0203	3171	241	552	552	211	445	445	280	879	879	Type I restriction-modification system, restriction subunit R (EC 3.1.21.3)
Mfumv2_0207	1017	56	22	41	84	25	57	79	43	80	mrr restriction system protein
Mfumv2_0208	315	334	76	76	348	73	73	231	72	72	hypothetical protein
Mfumv2_0209	141	20	2	2	85	8	8	43	6	6	hypothetical protein
Mfumv2_0210	195	1008	137	142	1524	197	198	2312	444	447	hypothetical protein
Mfumv2_0211	1764	286	364	365	498	584	585	1108	1937	1938	hypothetical protein
Mfumv2_0212	165	0	0	0	27	3	3	79	13	13	hypothetical protein

Mfumv2_0213	198	14	2	2	30	4	4	15	3	3	hypothetical protein
Mfumv2_0215	204	75	11	11	44	6	6	99	20	20	hypothetical protein
Mfumv2_0216	678	6186	2886	3029	514	231	232	229	153	154	Phosphate transport system regulatory protein PhoU
Mfumv2_0217	552	291	116	116	264	96	97	148	81	81	GCN5-related N-acetyltransferase
Mfumv2_0218	588	82	35	35	56	22	22	154	90	90	transposase IS200-family protein
Mfumv2_0219	1173	572	484	485	667	520	521	634	737	737	Oxidoreductase (flavoprotein)
Mfumv2_0220	243	2273	399	399	2187	354	354	863	208	208	SirA family protein
Mfumv2_0221	414	2813	840	841	2299	632	634	1106	454	454	hypothetical protein
Mfumv2_0222	291	757	159	159	645	125	125	291	84	84	UspA domain protein
Mfumv2_0223	1335	30	29	29	27	24	24	77	102	102	Membrane fusion component of tripartite multidrug resistance system
Mfumv2_0224	1515	81	89	89	124	125	125	119	179	179	Inner membrane component of tripartite multidrug resistance system
Mfumv2_0225	132	31	3	3	34	2	3	53	7	7	hypothetical protein
Mfumv2_0226	1776	27	35	35	27	32	32	35	62	62	Outer membrane component of tripartite multidrug resistance system
Mfumv2_0227	531	31	12	12	57	20	20	57	30	30	probable membrane protein YPO0899
Mfumv2_0228	990	596	426	426	61	40	40	431	421	423	Riboflavin kinase (EC 2.7.1.26) / FMN adenylyltransferase (EC 2.7.7.2)
Mfumv2_0229	714	147	76	76	103	49	49	332	235	235	tRNA pseudouridine synthase B (EC 4.2.1.70)
Mfumv2_0230	984	236	168	168	195	128	128	485	473	473	FIG146085: 3'-to-5' oligoribonuclease A, Bacillus type
Mfumv2_0231	165	117	14	14	109	12	12	92	15	15	hypothetical protein
Mfumv2_0232	1929	380	529	529	287	369	369	328	626	627	Phosphomannomutase (EC 5.4.2.8)
Mfumv2_0233	1713	89	108	110	80	91	91	100	170	170	sodium/iodide co-transporter
Mfumv2_0234	174	40	5	5	26	3	3	133	23	23	hypothetical protein
Mfumv2_0235	918	297	197	197	378	231	231	172	157	157	Fructose-bisphosphate aldolase, archaeal class I (EC 4.1.2.13)
Mfumv2_0236	777	184	103	103	236	122	122	262	202	202	Acyl-[acyl-carrier-protein]--UDP-N-acetylglucosamine O-acyltransferase (EC 2.3.1.129)
Mfumv2_0237	1311	474	449	449	508	444	444	553	719	719	N-acetylglucosamine deacetylase / (3R)-hydroxymyristoyl-[acyl carrier protein] dehydratase
Mfumv2_0238	162	0	0	0	28	3	3	37	6	6	hypothetical protein
Mfumv2_0239	1638	44	52	52	71	76	77	153	249	249	Oligopeptide ABC transporter, periplasmic oligopeptide-binding protein OppA (TC 3.A.1.5.1)
Mfumv2_0240	513	335	123	124	208	71	71	252	128	128	Methylated-DNA--protein-cysteine methyltransferase (EC 2.1.1.63)
Mfumv2_0241	915	26	16	17	67	41	41	82	74	74	hypothetical protein
Mfumv2_0242	807	235	137	137	309	164	166	227	180	182	Oligopeptide transport ATP-binding protein OppF (TC 3.A.1.5.1)
Mfumv2_0243	843	128	78	78	119	67	67	230	192	192	Oligopeptide transport ATP-binding protein OppD (TC 3.A.1.5.1)
Mfumv2_0244	1035	72	54	54	120	83	83	148	152	152	Oligopeptide transport system permease protein OppC (TC 3.A.1.5.1)
Mfumv2_0245	969	63	44	44	76	49	49	113	109	109	Oligopeptide transport system permease protein OppB (TC 3.A.1.5.1)
Mfumv2_0246	1365	91	90	90	77	69	70	156	211	211	hypothetical protein
Mfumv2_0250	951	208	137	143	267	136	169	130	111	123	hypothetical protein
Mfumv2_0251	267	1665	321	321	1940	345	345	2984	790	790	SSU ribosomal protein S15p (S13e)
Mfumv2_0252	2130	1201	1845	1847	1776	2520	2520	2179	4598	4602	Polyribonucleotide nucleotidyltransferase (EC 2.7.7.8)
Mfumv2_0253	486	464	163	163	683	221	221	1145	552	552	bacterioferritin comigratory protein homolog
Mfumv2_0254	141	245	25	25	373	35	35	365	51	51	hypothetical protein
Mfumv2_0255	1278	555	512	512	1022	869	870	935	1184	1185	4-hydroxy-3-methylbut-2-enyl diphosphate reductase (EC 1.17.1.2)
Mfumv2_0256	1440	512	532	532	800	765	767	565	807	807	Cytosol aminopeptidase PepA (EC 3.4.11.1)
Mfumv2_0257	1359	223	219	219	373	338	338	229	309	309	hypothetical protein
Mfumv2_0258	828	1115	667	667	1090	599	601	1587	1303	1303	Predicted ATPase
Mfumv2_0259	717	798	411	413	1137	543	543	1765	1250	1255	hypothetical protein
Mfumv2_0260	465	259	87	87	303	94	94	167	77	77	Protein of unknown function UPF0074
Mfumv2_0261	831	37	22	22	31	16	17	55	43	45	hypothetical protein
Mfumv2_0262	741	73	39	39	197	97	97	182	134	134	Hypothetical protein YggS, proline synthase co-transcribed bacterial homolog PROSC
Mfumv2_0263	933	129	87	87	180	112	112	265	245	245	transcriptional regulator, LysR family
Mfumv2_0264	1965	240	341	341	290	379	380	524	1020	1021	Biosynthetic arginine decarboxylase (EC 4.1.1.19)
Mfumv2_0265	501	594	214	215	548	183	183	507	252	252	hypothetical protein
Mfumv2_0266	120	58	5	5	113	9	9	244	29	29	hypothetical protein

Mfumv2_0269	777	59	33	33	75	39	39	119	92	92	Pantoate--beta-alanine ligase (EC 6.3.2.1)
Mfumv2_0270	1008	335	244	244	481	323	323	392	392	392	N-acetylmuramoyl-L-alanine amidase (EC 3.5.1.28)
Mfumv2_0271	1221	177	155	156	204	166	166	255	308	309	Sensor protein zraS (EC 2.7.3.-)
Mfumv2_0272	1386	269	267	269	344	309	318	328	448	451	Response regulator of zinc sigma-54-dependent two-component system
Mfumv2_0273	1554	93	104	104	25	26	26	43	67	67	Protein of unknown function DUF1597
Mfumv2_0274	1590	273	185	314	31	18	33	470	475	741	hypothetical protein
Mfumv2_0275	1641	73	86	86	16	18	18	204	329	332	hypothetical protein
Mfumv2_0276	117	320	27	27	90	7	7	147	17	17	hypothetical protein
Mfumv2_0277	1221	981	865	865	1070	869	870	873	1057	1057	Aspartokinase (EC 2.7.2.4)
Mfumv2_0278	594	282	121	121	167	66	66	428	252	252	TPR repeats containing protein
Mfumv2_0279	675	125	61	61	211	94	95	136	91	91	hypothetical protein
Mfumv2_0280	126	11	1	1	95	8	8	72	9	9	hypothetical protein
Mfumv2_0281	129	21	2	2	23	2	2	55	7	7	hypothetical protein
Mfumv2_0282	2202	164	261	261	53	78	78	87	189	189	outer membrane autotransporter barrel
Mfumv2_0283	135	10	1	1	44	4	4	30	4	4	hypothetical protein
Mfumv2_0284	309	22	5	5	15	3	3	39	12	12	hypothetical protein
Mfumv2_0285	180	8	1	1	58	6	7	28	5	5	hypothetical protein
Mfumv2_0287	201	2432	345	353	1651	217	221	798	159	159	hypothetical protein
Mfumv2_0288	270	200	39	39	117	21	21	97	26	26	hypothetical protein
Mfumv2_0289	114	24	2	2	26	2	2	53	6	6	hypothetical protein
Mfumv2_0290	114	49	4	4	0	0	0	18	2	2	hypothetical protein
Mfumv2_0291	144	10	1	1	0	0	0	35	5	5	hypothetical protein
Mfumv2_0292	2520	127	231	231	120	201	202	130	325	325	Glycosyl transferase, group 2 family protein
Mfumv2_0293	1530	65	72	72	87	89	89	117	177	177	hypothetical protein
Mfumv2_0294	1197	45	39	39	64	51	51	118	140	140	hypothetical protein
Mfumv2_0298	2103	109	166	166	133	186	187	141	292	293	Glycosyl transferase, group 2 family protein
Mfumv2_0299	957	220	152	152	171	109	109	252	239	239	glycosyl transferase family 2
Mfumv2_0300	2484	87	155	156	30	50	50	37	92	92	Outer membrane receptor protein, mostly Fe transport
Mfumv2_0301	117	12	1	1	0	0	0	26	3	3	hypothetical protein
Mfumv2_0302	414	174	52	52	33	9	9	41	17	17	Rare lipoprotein A precursor
Mfumv2_0303	1038	55	41	41	43	30	30	51	52	52	hypothetical protein
Mfumv2_0304	921	69	46	46	75	46	46	81	74	74	Ribokinase (EC 2.7.1.15)
Mfumv2_0305	150	9	1	1	0	0	0	7	1	1	hypothetical protein
Mfumv2_0306	870	108	66	68	97	54	56	169	146	146	NAD kinase (EC 2.7.1.23)
Mfumv2_0308	456	52	17	17	112	34	34	71	32	32	hypothetical protein
Mfumv2_0309	585	140	59	59	421	164	164	290	168	168	hypothetical protein
Mfumv2_0310	1446	97	101	101	145	139	140	149	213	214	hypothetical protein
Mfumv2_0311	3228	44	101	102	77	166	166	42	136	136	Cobalt-zinc-cadmium resistance protein CzcA; Cation efflux system protein CusA
Mfumv2_0312	150	166	18	18	400	40	40	67	10	10	hypothetical protein
Mfumv2_0314	1302	29	27	27	29	25	25	43	55	56	Major facilitator superfamily
Mfumv2_0315	1314	70	65	66	65	57	57	68	88	88	major facilitator superfamily MFS_1
Mfumv2_0316	1929	172	239	240	137	176	176	240	460	460	hypothetical protein
Mfumv2_0317	828	2160	1281	1292	742	408	409	794	650	652	Indole-3-glycerol phosphate synthase (EC 4.1.1.48)
Mfumv2_0318	576	214	89	89	391	150	150	425	243	243	Phosphoribosylanthranilate isomerase (EC 5.3.1.24)
Mfumv2_0319	336	227	55	55	514	115	115	459	153	153	Tryptophan synthase beta chain (EC 4.2.1.20)
Mfumv2_0320	174	987	122	124	1312	151	152	974	166	168	hypothetical protein
Mfumv2_0322	141	29	3	3	53	5	5	150	20	21	hypothetical protein
Mfumv2_0323	474	231	79	79	212	67	67	387	182	182	Guanylate kinase (EC 2.7.4.8)
Mfumv2_0324	594	329	141	141	205	80	81	280	165	165	Phosphopantothenoylcysteine decarboxylase (EC 4.1.1.36) / Phosphopantothenoylcysteine synthetase (EC 6.3.2.5)
Mfumv2_0325	504	187	68	68	325	109	109	480	240	240	Phosphoribosylaminoimidazole carboxylase catalytic subunit (EC 4.1.1.21)

Mfumv2_0326	1155	159	133	133	270	208	208	270	309	309	Phosphoribosylaminoimidazole carboxylase ATPase subunit (EC 4.1.1.21)
Mfumv2_0327	213	52	8	8	155	22	22	137	29	29	Sulfur transfer protein involved in thiamine biosynthesis
Mfumv2_0328	1140	168	137	138	253	192	192	175	198	198	Thiazole biosynthesis protein ThiH
Mfumv2_0329	2562	245	451	453	357	606	609	461	1168	1172	Glycosyl transferase, group 1
Mfumv2_0330	804	72	42	42	118	63	63	186	148	148	hypothetical protein
Mfumv2_0331	2481	189	334	338	205	338	339	306	754	754	ATP-dependent protease La (EC 3.4.21.53) Type II
Mfumv2_0332	411	623	185	185	354	97	97	434	177	177	hypothetical protein
Mfumv2_0333	1017	1415	1039	1039	1025	694	694	1028	1036	1037	LysR family transcriptional regulator YeiE
Mfumv2_0334	1065	516	396	397	512	359	363	466	492	492	Radical SAM domain protein
Mfumv2_0335	555	67	27	27	78	29	29	114	63	63	Starvation lipoprotein Slp paralog
Mfumv2_0336	294	127	27	27	169	33	33	117	34	34	hypothetical protein
Mfumv2_0337	666	71	34	34	117	52	52	148	98	98	Ferric siderophore transport system, periplasmic binding protein TonB
Mfumv2_0338	1014	131	95	96	176	118	119	184	185	185	SAM-dependent methyltransferase
Mfumv2_0339	999	133	96	96	197	131	131	311	308	308	Glucokinase (EC 2.7.1.2)
Mfumv2_0340	1443	26	27	27	33	32	32	47	67	67	hypothetical protein
Mfumv2_0341	993	252	181	181	307	203	203	128	126	126	Putative oxidoreductase YncB
Mfumv2_0342	1584	113	128	129	90	95	95	151	237	237	Trehalose synthase (EC 5.4.99.16)
Mfumv2_0343	1995	169	243	243	114	151	151	276	545	546	Alpha-amylase (EC 3.2.1.1)
Mfumv2_0344	441	967	307	308	1362	400	400	432	189	189	hypothetical protein
Mfumv2_0345	384	94	26	26	199	51	51	223	85	85	Uncharacterized enzyme of heme biosynthesis
Mfumv2_0346	594	816	347	350	1347	533	533	732	431	431	hypothetical protein
Mfumv2_0347	996	138	99	99	137	91	91	215	212	212	Methylamine dehydrogenase heavy chain precursor (EC 1.4.99.3)
Mfumv2_0348	543	43	17	17	55	20	20	59	32	32	Methylamine utilization protein MauE
Mfumv2_0349	657	234	111	111	144	63	63	103	67	67	Methylamine utilization protein MauD
Mfumv2_0350	543	125	49	49	138	50	50	152	82	82	Methylamine dehydrogenase light chain precursor (EC 1.4.99.3)
Mfumv2_0351	402	31	9	9	26	7	7	55	22	22	Cytochrome c, class I
Mfumv2_0352	123	34	3	3	24	2	2	33	4	4	hypothetical protein
Mfumv2_0353	1233	265	236	236	275	225	226	357	437	437	Glycosyltransferase
Mfumv2_0354	726	95	50	50	145	69	70	154	111	111	Acetyltransferase, GNAT family
Mfumv2_0355	705	43	22	22	40	19	19	116	81	81	hypothetical protein
Mfumv2_0356	2415	112	196	196	102	164	164	370	884	886	glycosyl transferase, group 1
Mfumv2_0357	1731	254	317	317	304	350	350	348	597	597	Oligoendopeptidase F (EC 3.4.24.-)
Mfumv2_0358	348	1468	369	369	1130	262	262	496	171	171	Ribosome hibernation protein YhbH
Mfumv2_0359	1590	371	424	426	523	554	554	472	740	744	(R)-citramalate synthase (EC 2.3.1.182)
Mfumv2_0360	177	4607	587	589	5641	664	665	3601	632	632	hypothetical protein
Mfumv2_0361	840	89	54	54	64	36	36	186	155	155	Prolipoprotein diacylglyceryl transferase (EC 2.4.99.-)
Mfumv2_0362	1890	1227	1671	1675	555	697	699	506	949	949	Threonyl-tRNA synthetase (EC 6.1.1.3)
Mfumv2_0363	618	1001	446	447	1173	483	483	932	570	571	Translation initiation factor 3
Mfumv2_0364	216	3378	527	527	2732	393	393	2484	532	532	ribosomal protein L35
Mfumv2_0365	165	1536	183	183	2275	250	250	1962	321	321	hypothetical protein
Mfumv2_0366	1155	629	525	525	663	510	510	988	1132	1132	1-deoxy-D-xylulose 5-phosphate reductoisomerase (EC 1.1.1.267)
Mfumv2_0367	1437	438	455	455	452	433	433	532	758	758	Membrane-associated zinc metalloprotease
Mfumv2_0368	1755	622	788	789	874	1022	1022	640	1113	1114	1-hydroxy-2-methyl-2-(E)-butenyl 4-diphosphate synthase (EC 1.17.7.1)
Mfumv2_0369	936	2235	1510	1511	1739	1083	1084	855	794	794	Gentisate 1,2-dioxygenase (EC 1.13.11.4)
Mfumv2_0370	360	38	10	10	25	6	6	11	4	4	hypothetical protein
Mfumv2_0371	192	743	102	103	813	104	104	882	168	168	Twin-arginine translocation protein TatA
Mfumv2_0372	225	166	27	27	153	23	23	368	82	82	hypothetical protein
Mfumv2_0373	927	245	163	164	277	171	171	1578	1451	1451	Radical SAM protein required for addition of adenosine to hopane skeleton, HpnH
Mfumv2_0374	375	354	96	96	593	146	148	1565	581	582	hypothetical protein
Mfumv2_0375	642	220	102	102	208	89	89	441	281	281	protein of unknown function DUF152

Mfumv2_0377	354	66	17	17	25	6	6	66	23	23	hypothetical protein
Mfumv2_0378	525	158	60	60	372	130	130	177	92	92	Molybdopterin oxidoreductase subunit, predicted; chaperone protein HtpG
Mfumv2_0379	3138	219	496	496	298	623	623	220	684	684	Molybdopterin oxidoreductase, iron-sulfur binding subunit (EC 1.2.7.-)
Mfumv2_0380	2028	161	236	236	158	214	214	133	267	267	Molybdopterin oxidoreductase (EC 1.2.7.-)
Mfumv2_0381	618	181	80	81	248	102	102	302	184	185	ABC-type Fe3+ transport system protein; Molybdenum transport protein, putative
Mfumv2_0382	1203	227	197	197	271	215	217	119	142	142	Putative uncharacterized protein TTHA1760
Mfumv2_0383	372	283	76	76	537	133	133	737	268	272	hypothetical protein
Mfumv2_0384	1446	90	94	94	118	114	114	88	126	126	Cytochrome c oxidase subunit CcoN (EC 1.9.3.1)
Mfumv2_0385	597	366	158	158	327	129	130	157	93	93	Cytochrome c oxidase subunit CcoO (EC 1.9.3.1)
Mfumv2_0386	639	139	64	64	265	113	113	227	144	144	cytochrome c, class I
Mfumv2_0387	1848	736	982	982	795	977	979	766	1404	1404	Cytochrome c oxidase polypeptide I (EC 1.9.3.1)
Mfumv2_0388	792	1439	823	823	1295	682	683	763	599	599	Cytochrome c oxidase polypeptide III (EC 1.9.3.1)
Mfumv2_0389	297	788	168	169	733	145	145	540	159	159	hypothetical protein
Mfumv2_0390	198	944	135	135	1213	160	160	886	174	174	hypothetical protein
Mfumv2_0391	285	782	161	161	1728	328	328	807	228	228	hypothetical protein
Mfumv2_0392	792	883	501	505	1526	805	805	532	418	418	Alternative cytochrome c oxidase polypeptide CoxM (EC 1.9.3.1)
Mfumv2_0393	450	357	116	116	571	171	171	327	146	146	hypothetical protein
Mfumv2_0394	615	79	35	35	129	53	53	118	72	72	hypothetical protein
Mfumv2_0395	1128	128	104	104	152	114	114	181	202	202	conserved hypothetical protein
Mfumv2_0396	243	34	6	6	19	3	3	237	57	57	hypothetical protein
Mfumv2_0397	309	4521	1006	1009	3955	814	814	6857	2100	2101	hypothetical protein
Mfumv2_0398	153	380	42	42	383	38	39	270	41	41	hypothetical protein
Mfumv2_0399	2043	75	111	111	73	99	99	113	229	229	alpha-amylase 1, putative
Mfumv2_0400	2046	137	202	202	176	240	240	239	484	485	glycoside hydrolase family 57
Mfumv2_0401	1029	147	109	109	121	83	83	578	590	590	Galactose-1-phosphate uridylyltransferase (EC 2.7.7.10)
Mfumv2_0402	120	0	0	0	25	2	2	25	3	3	hypothetical protein
Mfumv2_0403	1143	38	31	31	35	26	27	152	172	172	tRNA-guanine transglycosylase (EC 2.4.2.29)
Mfumv2_0404	423	484	148	148	635	179	179	1185	496	497	Preprotein translocase subunit YajC (TC 3.A.5.1.1)
Mfumv2_0405	2295	243	402	403	327	500	500	522	1188	1188	Protein-export membrane protein SecD / Protein-export membrane protein SecF (TC 3.A.5.1.1)
Mfumv2_0406	732	191	101	101	353	171	172	413	300	300	Menaquinone via futalosine step 1
Mfumv2_0407	1101	390	310	310	574	421	421	554	604	605	Menaquinone via futalosine step 3
Mfumv2_0408	3234	334	779	780	383	823	826	517	1658	1658	Carbamoyl-phosphate synthase large chain (EC 6.3.5.5)
Mfumv2_0409	471	200	68	68	147	46	46	182	85	85	Membrane protein, distant similarity to thiosulphate:quinone oxidoreductase DoxD
Mfumv2_0410	1020	72	53	53	62	42	42	66	67	67	COG1408: Predicted phosphohydrolases
Mfumv2_0411	2349	73	123	124	73	114	114	85	196	198	Outer membrane receptor protein, mostly Fe transport
Mfumv2_0412	720	523	272	272	390	187	187	269	192	192	Phosphoribosylformimino-5-aminoimidazole carboxamide ribotide isomerase (EC 5.3.1.16)
Mfumv2_0413	639	158	73	73	169	72	72	546	346	346	Imidazole glycerol phosphate synthase amidotransferase subunit (EC 2.4.2.-)
Mfumv2_0414	822	296	176	176	468	256	256	248	202	202	Diaminopimelate epimerase (EC 5.1.1.7)
Mfumv2_0415	906	466	304	305	492	297	297	552	495	496	Dihydrodipicolinate synthase (EC 4.2.1.52)
Mfumv2_0416	744	190	102	102	305	151	151	504	372	372	Dihydrodipicolinate reductase (EC 1.3.1.26)
Mfumv2_0417	711	101	52	52	135	64	64	214	151	151	Methyltransferase type 11
Mfumv2_0418	177	172	22	22	59	7	7	51	9	9	hypothetical protein
Mfumv2_0419	150	83	9	9	110	11	11	54	8	8	hypothetical protein
Mfumv2_0420	141	314	32	32	170	16	16	107	15	15	hypothetical protein
Mfumv2_0421	1566	1202	1359	1359	825	861	861	636	986	987	hypothetical protein
Mfumv2_0422	126	154	14	14	143	12	12	216	27	27	hypothetical protein
Mfumv2_0423	135	390	38	38	378	34	34	105	14	14	hypothetical protein
Mfumv2_0424	150	5603	586	607	4054	383	405	2044	292	304	hypothetical protein
Mfumv2_0425	114	2053	153	169	1370	98	104	602	61	68	hypothetical protein
Mfumv2_0426	144	4884	456	508	2565	208	246	791	109	113	hypothetical protein

Mfumv2_0427	117	3077	248	260	2066	146	161	629	70	73	hypothetical protein
Mfumv2_0428	177	10052	1237	1285	5683	645	670	2581	430	453	hypothetical protein
Mfumv2_0429	378	11	3	3	36	9	9	59	22	22	hypothetical protein
Mfumv2_0430	1008	12	9	9	28	19	19	26	26	26	CRISPR-associated protein Cas1
Mfumv2_0431	1113	160	128	129	101	75	75	91	100	100	hypothetical protein
Mfumv2_0432	954	118	81	81	154	98	98	141	133	133	CRISPR-associated protein, Csx3 family
Mfumv2_0433	1095	78	62	62	108	79	79	132	143	143	CRISPR-associated protein TM1812
Mfumv2_0434	165	25	3	3	36	4	4	214	35	35	hypothetical protein
Mfumv2_0435	480	257	89	89	178	57	57	141	67	67	hypothetical protein
Mfumv2_0436	1383	279	277	279	284	261	262	203	277	278	CRISPR-associated RAMP Cmr6
Mfumv2_0437	366	178	47	47	139	34	34	204	74	74	hypothetical protein
Mfumv2_0438	951	195	134	134	268	170	170	141	132	133	CRISPR-associated RAMP Cmr4
Mfumv2_0439	1299	249	234	234	265	229	229	175	224	225	CRISPR-associated RAMP Cmr3
Mfumv2_0440	2997	150	325	325	173	344	345	154	457	458	CRISPR-associated RAMP Cmr2
Mfumv2_0442	168	58	7	7	277	24	31	54	7	9	hypothetical protein
Mfumv2_0443	1887	98	134	134	136	171	171	130	243	243	Adenosine deaminase (EC 3.5.4.4)
Mfumv2_0444	1272	652	598	599	600	508	508	407	513	513	CRISPR-associated protein TM1795 family-like
Mfumv2_0445	177	8	1	1	8	1	1	23	4	4	hypothetical protein
Mfumv2_0446	159	87	10	10	330	32	35	82	12	13	hypothetical protein
Mfumv2_0447	1416	116	119	119	67	62	63	31	42	43	Undecaprenyl-phosphate galactosephosphotransferase (EC 2.7.8.6)
Mfumv2_0448	1047	4160	3142	3146	3305	2304	2305	1561	1620	1621	hypothetical protein
Mfumv2_0449	1137	521	426	428	473	358	358	431	486	486	Capsule polysaccharide export protein
Mfumv2_0450	2322	274	459	459	239	369	369	191	439	440	exopolysaccharide biosynthesis protein
Mfumv2_0451	1182	80	67	68	91	72	72	71	83	83	hypothetical protein
Mfumv2_0452	1413	68	69	69	56	52	53	56	78	78	hypothetical protein
Mfumv2_0453	1230	136	121	121	151	124	124	147	178	179	a-glycosyltransferase, glycosyltransferase family 4 protein(EC:2.4.1.-)
Mfumv2_0457	687	54	27	27	118	54	54	185	125	126	hypothetical protein
Mfumv2_0458	555	307	123	123	379	140	140	153	84	84	3-polyprenyl-4-hydroxybenzoate carboxy-lyase UbiX (EC 4.1.1.-)
Mfumv2_0459	873	147	93	93	160	93	93	171	147	148	Menaquinone via futalosine polyprenyltransferase (MenA homolog)
Mfumv2_0460	1176	174	148	148	272	213	213	376	439	439	Gene SCO4494, often clustered with other genes in menaquinone via futalosine pathway
Mfumv2_0461	2049	1466	2165	2169	2075	2827	2832	1842	3742	3743	Cell division protein FtsH (EC 3.4.24.-)
Mfumv2_0462	270	354	69	69	106	19	19	239	64	64	hypothetical protein
Mfumv2_0468	177	704	90	90	517	61	61	142	25	25	hypothetical protein
Mfumv2_0469	141	776	79	79	213	20	20	286	40	40	hypothetical protein
Mfumv2_0470	126	0	0	0	12	1	1	16	2	2	hypothetical protein
Mfumv2_0471	1032	46	33	34	67	45	46	86	86	88	Glycosyl transferase, group 1 family protein
Mfumv2_0472	1050	152	115	115	132	92	92	101	105	105	Glycosyl transferase, group 1
Mfumv2_0473	1707	912	698	1125	762	482	866	424	455	717	hypothetical protein
Mfumv2_0476	1038	112	83	84	121	83	84	127	130	131	Glycosyltransferase (EC 2.4.1.-)
Mfumv2_0477	1371	93	92	92	83	76	76	111	146	151	hypothetical protein
Mfumv2_0478	1212	53	46	46	67	54	54	84	100	101	hypothetical protein
Mfumv2_0479	1347	68	65	66	93	83	83	74	97	99	hypothetical protein
Mfumv2_0480	1032	94	70	70	129	88	89	60	61	61	Uncharacterized protein
Mfumv2_0481	4068	605	1771	1778	120	325	325	65	264	264	hypothetical protein
Mfumv2_0482	951	22	15	15	28	18	18	88	82	83	Methyltransferase
Mfumv2_0483	858	252	155	156	126	72	72	145	122	123	Glycosyl transferase, family 2
Mfumv2_0485	699	83	42	42	64	30	30	68	47	47	Methyltransferase FkbM
Mfumv2_0486	1707	1061	845	1308	582	412	662	334	397	565	hypothetical protein
Mfumv2_0491	153	72	8	8	98	10	10	66	10	10	outer membrane efflux protein
Mfumv2_0492	924	69	46	46	50	31	31	121	110	111	Glycosyl transferase, group 2 family protein

Mfumv2_0493	786	159	90	90	210	110	110	222	173	173	Methyltransferase type 11
Mfumv2_0494	1200	159	138	138	134	107	107	99	117	118	glycosyl transferase, group 1
Mfumv2_0495	156	44	5	5	48	5	5	91	14	14	hypothetical protein
Mfumv2_0496	138	60	6	6	22	2	2	73	10	10	hypothetical protein
Mfumv2_0497	162	51	6	6	9	1	1	81	13	13	hypothetical protein
Mfumv2_0498	2160	89	139	139	76	109	110	181	387	387	hypothetical protein
Mfumv2_0499	1032	789	586	588	662	454	455	615	629	629	Cytochrome c551 peroxidase (EC 1.11.1.5)
Mfumv2_0500	1803	239	311	311	353	424	424	239	425	427	hypothetical protein
Mfumv2_0501	1359	180	177	177	234	212	212	197	264	265	Glutamate-1-semialdehyde aminotransferase (EC 5.4.3.8)
Mfumv2_0502	1887	233	309	317	421	518	529	313	582	586	DNA polymerase X family
Mfumv2_0503	171	16	2	2	79	9	9	24	4	4	hypothetical protein
Mfumv2_0504	864	104	64	65	120	69	69	117	99	100	Dihydropteroate synthase (EC 2.5.1.15)
Mfumv2_0505	744	80	43	43	101	50	50	91	67	67	Hypothetical protein YbbP, contains nucleotide-binding domain of DisA bacterial checkpoint controller
Mfumv2_0506	138	221	22	22	141	13	13	139	19	19	hypothetical protein
Mfumv2_0507	219	82	13	13	137	20	20	253	55	55	hypothetical protein
Mfumv2_0508	1374	240	238	238	303	277	277	297	405	405	Phosphoglucosamine mutase (EC 5.4.2.10)
Mfumv2_0509	657	107	51	51	272	119	119	365	238	238	hypothetical protein
Mfumv2_0510	579	187	78	78	119	46	46	108	62	62	Orotate phosphoribosyltransferase (EC 2.4.2.10)
Mfumv2_0511	114	340	28	28	461	34	35	133	15	15	hypothetical protein
Mfumv2_0512	474	137	47	47	231	72	73	87	41	41	hypothetical protein
Mfumv2_0513	951	329	225	226	541	343	343	323	304	305	hypothetical protein
Mfumv2_0514	1635	75	88	88	78	85	85	64	102	103	Proposed peptidoglycan lipid II flippase MurJ
Mfumv2_0515	996	79	57	57	119	79	79	75	74	74	ADP-heptose--lipooligosaccharide heptosyltransferase II (EC 2.4.1.-)
Mfumv2_0516	1050	200	152	152	180	126	126	246	255	256	Tetraacyldisaccharide 4'-kinase (EC 2.7.1.130)
Mfumv2_0517	861	111	68	69	56	32	32	212	180	181	Spermidine/putrescine-binding periplasmic protein
Mfumv2_0518	492	1835	652	652	1996	654	654	1453	709	709	hypothetical protein
Mfumv2_0519	2538	468	857	857	531	897	898	743	1867	1870	hypothetical protein
Mfumv2_0520	381	342	94	94	430	108	109	371	139	140	Protein of unknown function, DUF1844 family
Mfumv2_0521	1011	230	168	168	346	233	233	307	308	308	Molybdenum cofactor biosynthesis protein MoaA
Mfumv2_0522	279	149	30	30	339	63	63	304	84	84	hypothetical protein
Mfumv2_0523	399	142	41	41	309	82	82	374	148	148	Molybdopterin biosynthesis MoaE
Mfumv2_0525	1917	699	967	968	1271	1620	1623	991	1883	1884	Sulfate adenylyltransferase subunit 1 (EC 2.7.7.4) / Adenylylsulfate kinase (EC 2.7.1.25)
Mfumv2_0526	804	184	106	107	1189	637	637	1085	865	865	Sulfate adenylyltransferase subunit 2 (EC 2.7.7.4)
Mfumv2_0527	720	129	67	67	557	266	267	221	158	158	Phosphoadenylyl-sulfate reductase [thioredoxin] (EC 1.8.4.8)
Mfumv2_0528	1161	32	27	27	47	35	36	147	169	169	Homocitrate synthase (EC 2.3.3.14)
Mfumv2_0529	114	61	5	5	13	1	1	115	13	13	hypothetical protein
Mfumv2_0530	894	887	573	573	202	120	120	104	92	92	Nitrogenase (molybdenum-iron) reductase and maturation protein NifH
Mfumv2_0531	1476	397	423	423	81	80	80	68	99	99	Nitrogenase (molybdenum-iron) alpha chain (EC 1.18.6.1)
Mfumv2_0532	1581	723	825	826	106	112	112	147	230	230	Nitrogenase (molybdenum-iron) beta chain (EC 1.18.6.1)
Mfumv2_0533	1380	740	738	738	107	98	98	122	167	167	Nitrogenase FeMo-cofactor scaffold and assembly protein NifE
Mfumv2_0534	1344	229	222	222	61	55	55	91	121	121	Nitrogenase FeMo-cofactor scaffold and assembly protein NifN
Mfumv2_0535	399	274	79	79	98	26	26	78	30	31	Nitrogenase FeMo-cofactor carrier protein NifX
Mfumv2_0536	471	323	110	110	121	38	38	73	34	34	NifX-associated protein
Mfumv2_0537	192	130	18	18	0	0	0	68	13	13	hypothetical protein
Mfumv2_0538	579	708	295	296	913	352	352	853	489	490	Inorganic pyrophosphatase (EC 3.6.1.1)
Mfumv2_0539	129	118	11	11	105	9	9	797	101	102	hypothetical protein
Mfumv2_0540	393	173	49	49	130	34	34	472	183	184	transcriptional regulator, MerR family
Mfumv2_0541	114	24	2	2	53	4	4	257	28	29	hypothetical protein
Mfumv2_0542	1653	13	15	16	25	27	27	73	119	120	Mercuric ion reductase (EC 1.16.1.1)
Mfumv2_0543	1569	46	52	52	21	22	22	31	48	48	Nitrogenase FeMo-cofactor synthesis FeS core scaffold and assembly protein NifB

Mfumv2_0544	225	49	8	8	47	7	7	76	17	17	4Fe-4S ferredoxin, nitrogenase-associated
Mfumv2_0545	342	109	27	27	31	7	7	71	24	24	probable iron binding protein from the HesB_IscA_SufA family in Nif operon
Mfumv2_0546	516	67	25	25	35	12	12	39	20	20	hypothetical protein
Mfumv2_0547	822	130	76	77	80	44	44	249	198	203	LRV (FeS)4 cluster domain protein clustered with nitrogenase cofactor synthesis
Mfumv2_0548	282	137	28	28	128	24	24	104	29	29	NifZ protein
Mfumv2_0549	1167	123	104	104	95	74	74	121	140	140	Cysteine desulfurase (EC 2.8.1.7)
Mfumv2_0550	594	51	22	22	45	18	18	124	73	73	Nitrogenase FeMo-cofactor synthesis molybdenum delivery protein NifQ
Mfumv2_0551	858	147	91	91	182	104	104	167	142	142	Thiosulfate sulfurtransferase, rhodanese (EC 2.8.1.1)
Mfumv2_0552	1590	33	38	38	20	21	21	123	194	194	Nitrogenase (molybdenum-iron)-specific transcriptional regulator NifA
Mfumv2_0553	402	24	7	7	7	2	2	20	8	8	probable iron binding protein from the HesB_IscA_SufA family
Mfumv2_0554	330	63	15	15	18	4	4	12	4	4	Iron binding protein IscA for iron-sulfur cluster assembly
Mfumv2_0555	210	46	7	7	14	2	2	72	15	15	NifT protein
Mfumv2_0556	1149	57	47	47	26	20	20	111	127	127	DegT/DnrJ/EryC1/StrS aminotransferase
Mfumv2_0557	225	31	5	5	20	3	3	81	18	18	hypothetical protein
Mfumv2_0558	873	146	92	92	36	21	21	51	44	44	hypothetical protein
Mfumv2_0559	408	136	40	40	59	16	16	116	47	47	Nitrogenase stabilizing/protective protein NifW
Mfumv2_0560	840	166	100	101	32	18	18	73	61	61	Electron transfer flavoprotein, beta subunit
Mfumv2_0561	1125	252	205	205	44	33	33	54	60	60	Electron transfer flavoprotein, alpha subunit
Mfumv2_0562	1299	246	231	231	104	90	90	82	104	105	Probable electron transfer flavoprotein-quinone oxidoreductase FixC (EC 1.5.5.-)
Mfumv2_0563	285	646	132	133	248	47	47	166	47	47	Ferredoxin-like protein
Mfumv2_0564	696	2425	1218	1219	1797	833	833	726	500	501	hypothetical protein
Mfumv2_0565	1488	268	288	288	239	236	237	293	432	432	UbiD family decarboxylase associated with menaquinone via futalosine
Mfumv2_0566	1014	184	135	135	195	132	132	231	232	232	UDP-glucose 4-epimerase (EC 5.1.3.2)
Mfumv2_0567	363	114	30	30	124	30	30	100	36	36	hypothetical protein
Mfumv2_0568	411	71	21	21	128	35	35	113	46	46	hypothetical protein
Mfumv2_0569	471	247	84	84	284	88	89	265	124	124	hypothetical protein
Mfumv2_0571	147	160	13	17	276	12	27	48	5	7	hypothetical protein
Mfumv2_0573	420	422	84	128	175	29	49	125	36	52	hypothetical protein
Mfumv2_0574	2109	27	41	41	18	25	25	42	87	87	Molybdopterin oxidoreductase (EC 1.2.7.-)
Mfumv2_0575	879	112	71	71	82	48	48	211	183	184	DNA polymerase I (EC 2.7.7.7)
Mfumv2_0576	363	400	105	105	422	102	102	236	85	85	probable iron binding protein from the HesB_IscA_SufA family
Mfumv2_0577	1290	151	141	141	221	190	190	106	135	135	hypothetical protein
Mfumv2_0578	633	1424	651	651	2021	849	852	430	270	270	hypothetical protein
Mfumv2_0579	507	180	66	66	154	52	52	219	110	110	Crossover junction endodeoxyribonuclease RuvC (EC 3.1.22.4)
Mfumv2_0580	636	83	38	38	73	31	31	92	58	58	Holliday junction DNA helicase RuvA
Mfumv2_0581	1017	125	90	92	128	87	87	131	132	132	Holliday junction DNA helicase RuvB
Mfumv2_0582	417	750	226	226	702	195	195	520	215	215	Uncharacterized protein TM0723
Mfumv2_0583	564	552	224	225	532	200	200	874	489	489	rhodanese-like domain protein
Mfumv2_0584	1251	634	572	573	706	588	588	772	958	958	hypothetical protein
Mfumv2_0585	453	572	187	187	553	167	167	1939	871	871	hypothetical protein
Mfumv2_0586	207	54	8	8	58	8	8	166	33	34	hypothetical protein
Mfumv2_0587	183	83	11	11	82	10	10	127	22	23	hypothetical protein
Mfumv2_0588	645	105	49	49	147	63	63	158	101	101	Deoxyribose-phosphate aldolase (EC 4.1.2.4)
Mfumv2_0589	1020	133	98	98	141	96	96	142	144	144	hypothetical protein
Mfumv2_0590	729	36	19	19	35	17	17	53	37	38	Ribosomal RNA small subunit methyltransferase E (EC 2.1.1.-)
Mfumv2_0591	930	52	35	35	79	49	49	137	126	126	ADP-L-glycero-D-manno-heptose-6-epimerase (EC 5.1.3.20)
Mfumv2_0592	1551	212	237	237	335	346	346	286	439	440	ATP-dependent Clp protease ATP-binding subunit ClpX
Mfumv2_0595	1179	181	154	154	204	160	160	339	396	396	hypothetical protein
Mfumv2_0596	1008	29	20	21	28	19	19	91	91	91	hypothetical protein
Mfumv2_0597	1521	737	808	810	1042	1056	1056	735	1109	1109	Aldehyde dehydrogenase (EC 1.2.1.3)

Mfumv2_0601	678	176	85	86	204	92	92	199	134	134	Ribulose-phosphate 3-epimerase (EC 5.1.3.1)
Mfumv2_0602	384	191	53	53	250	64	64	213	80	81	Lactoylglutathione lyase (EC 4.4.1.5)
Mfumv2_0603	1065	66	51	51	86	61	61	167	175	176	Endoglucanase (EC 3.2.1.4)
Mfumv2_0604	138	90	9	9	109	10	10	117	16	16	hypothetical protein
Mfumv2_0605	192	1406	195	195	993	125	127	604	113	115	hypothetical protein
Mfumv2_0606	693	48	24	24	54	25	25	63	43	43	DedA protein
Mfumv2_0607	489	2758	973	974	3076	1000	1002	969	470	470	heat shock protein Hsp20
Mfumv2_0608	990	648	462	463	755	498	498	656	644	644	Opacity protein or related surface antigen
Mfumv2_0609	321	483	112	112	379	81	81	342	109	109	hypothetical protein
Mfumv2_0610	624	222	100	100	416	172	173	495	306	306	(Acyl-carrier-protein) phosphodiesterase(EC:3.1.4.14)
Mfumv2_0611	1548	169	188	189	229	233	236	173	266	266	hypothetical protein
Mfumv2_0612	450	89	29	29	37	11	11	36	15	16	Thiol peroxidase, Bcp-type (EC 1.11.1.15)
Mfumv2_0613	1362	177	174	174	207	188	188	161	217	217	Fe-S protein, homolog of lactate dehydrogenase SO1521
Mfumv2_0615	531	258	99	99	325	115	115	617	325	325	hypothetical protein
Mfumv2_0616	912	144	95	95	165	100	100	305	275	276	hypothetical protein
Mfumv2_0617	450	105	33	34	157	47	47	170	76	76	4-hydroxybenzoyl-CoA thioesterase family active site
Mfumv2_0618	996	103	74	74	92	60	61	141	139	139	hypothetical protein
Mfumv2_0619	1563	147	166	166	154	160	160	216	335	335	NAD(P)HX epimerase / NAD(P)HX dehydratase
Mfumv2_0620	1173	345	292	292	393	307	307	664	772	772	Sulfite reductase [NADPH] flavoprotein alpha-component (EC 1.8.1.2)
Mfumv2_0621	948	38	26	26	32	20	20	128	120	120	Mannose-6-phosphate isomerase (EC 5.3.1.8)
Mfumv2_0622	783	117	66	66	58	30	30	81	63	63	FIG003003: hypothetical protein
Mfumv2_0623	684	312	154	154	415	188	189	304	206	206	Phosphoribosyltransferase
Mfumv2_0624	2079	228	341	342	339	469	470	300	617	619	Excinuclease ABC subunit B
Mfumv2_0625	759	232	127	127	293	148	148	304	229	229	Uridylate kinase (EC 2.7.4.-)
Mfumv2_0626	618	567	253	253	794	326	327	335	204	205	Ribosome recycling factor
Mfumv2_0627	582	457	192	192	673	261	261	451	260	260	ThiJ/PfpI family protein
Mfumv2_0628	1146	151	123	125	161	122	123	148	168	168	FAD dependent oxidoreductase
Mfumv2_0629	2601	3535	6621	6640	1408	2429	2440	138	355	355	hypothetical protein
Mfumv2_0631	1053	80	61	61	87	61	61	149	156	156	Mannose-1-phosphate guanylyltransferase (GDP) (EC 2.7.7.22)
Mfumv2_0632	756	451	246	246	393	198	198	212	159	159	3-oxoacyl-[acyl-carrier protein] reductase (EC 1.1.1.100)
Mfumv2_0633	447	149	48	48	154	46	46	63	28	28	hypothetical protein
Mfumv2_0634	342	158	39	39	123	28	28	109	37	37	hypothetical protein
Mfumv2_0635	195	142	20	20	92	12	12	160	31	31	hypothetical protein
Mfumv2_0636	501	41	15	15	57	18	19	60	29	30	GCN5-related N-acetyltransferase
Mfumv2_0637	1365	232	229	229	231	210	210	151	205	205	Amino acid permease
Mfumv2_0638	126	55	5	5	0	0	0	48	6	6	hypothetical protein
Mfumv2_0639	384	361	100	100	235	59	60	307	117	117	hypothetical protein
Mfumv2_0640	525	42	16	16	66	23	23	207	108	108	RNA polymerase sigma-70 factor, ECF subfamily
Mfumv2_0641	225	345	56	56	214	32	32	264	59	59	hypothetical protein
Mfumv2_0642	651	262	123	123	187	81	81	198	128	128	Transfer origin protein, TraL, ATPase
Mfumv2_0643	531	183	70	70	229	81	81	179	94	94	hypothetical protein
Mfumv2_0646	1047	89	67	67	166	116	116	105	109	109	Rieske (2Fe-2S) domain protein
Mfumv2_0648	948	114	77	78	152	96	96	128	119	120	hypothetical protein
Mfumv2_0649	1224	6673	5849	5899	1846	1496	1505	55	67	67	Probable Co/Zn/Cd efflux system membrane fusion protein
Mfumv2_0650	3231	198	459	461	80	172	172	71	228	228	Cobalt-zinc-cadmium resistance protein CzcA; Cation efflux system protein CusA
Mfumv2_0651	543	135	53	53	149	54	54	98	51	53	Starvation lipoprotein Slp paralog
Mfumv2_0652	153	0	0	0	39	4	4	46	7	7	hypothetical protein
Mfumv2_0653	1359	49	47	48	54	49	49	72	97	97	MscS Mechanosensitive ion channel
Mfumv2_0654	765	85	47	47	137	70	70	146	111	111	hypothetical protein
Mfumv2_0655	747	54	29	29	72	36	36	127	94	94	hypothetical protein

Mfumv2_0656	1155	43	36	36	75	57	58	101	116	116	glycosyl transferase, group 1
Mfumv2_0657	807	60	35	35	99	53	53	134	107	107	HAD superfamily hydrolase
Mfumv2_0658	642	772	357	358	814	348	348	1227	780	781	Ferric siderophore transport system, biopolymer transport protein ExbB
Mfumv2_0659	447	362	117	117	373	111	111	932	412	413	Biopolymer transport protein ExbD/TolR
Mfumv2_0660	582	333	140	140	397	154	154	658	379	380	Periplasmic protein TonB
Mfumv2_0661	1488	89	96	96	120	119	119	191	282	282	Anthranilate synthase, aminase component (EC 4.1.3.27)
Mfumv2_0662	606	224	98	98	297	119	120	358	215	215	Anthranilate synthase, amidotransferase component (EC 4.1.3.27)
Mfumv2_0663	1257	192	174	174	313	262	262	333	415	415	NAD-specific glutamate dehydrogenase (EC 1.4.1.2); NADP-specific glutamate dehydrogenase (EC 1.4.1.4)
Mfumv2_0664	957	72	50	50	78	50	50	110	103	104	dienelactone hydrolase family protein
Mfumv2_0665	2070	377	559	564	725	997	999	1027	2062	2109	Squalene--hopene cyclase (EC 5.4.99.17)
Mfumv2_0666	696	372	187	187	742	343	344	987	680	681	Nucleoside phosphorylase
Mfumv2_0667	342	202	50	50	281	64	64	333	113	113	probable iron binding protein from the HesB_IscA_SufA family
Mfumv2_0668	2481	258	462	463	251	414	414	510	1255	1255	Leucyl-tRNA synthetase (EC 6.1.1.4)
Mfumv2_0669	1344	63	61	61	99	89	89	128	171	171	TldE/PmbA family protein, Actinobacterial subgroup
Mfumv2_0670	1584	199	228	228	277	292	292	150	235	235	TldD family protein, Actinobacterial subgroup
Mfumv2_0671	1248	700	631	631	899	747	747	673	833	833	Acetylornithine deacetylase (EC 3.5.1.16)
Mfumv2_0672	540	423	165	165	503	181	181	614	329	329	Ferritin, Dps family protein
Mfumv2_0673	765	103	57	57	82	41	42	117	89	89	Chromosome (plasmid) partitioning protein ParA / Sporulation initiation inhibitor protein Soj
Mfumv2_0674	939	201	136	136	261	163	163	655	610	610	ABC transporter related
Mfumv2_0675	750	87	47	47	120	60	60	234	174	174	ABC-type transport system involved in multi-copper enzyme maturation, permease component
Mfumv2_0676	1464	274	288	290	358	349	349	501	727	727	Mucin 2 precursor
Mfumv2_0677	1800	254	328	330	489	586	586	350	624	625	hypothetical protein
Mfumv2_0678	555	142	57	57	105	38	39	263	145	145	hypothetical protein
Mfumv2_0679	612	38	17	17	32	13	13	122	74	74	Transcriptional regulator, TetR family
Mfumv2_0680	1044	42	32	32	62	43	43	78	81	81	Membrane fusion component of tripartite multidrug resistance system
Mfumv2_0681	369	574	153	153	387	95	95	410	150	150	HNH endonuclease
Mfumv2_0682	1809	126	165	165	115	138	139	126	226	226	Polymyxin resistance protein ArnT, undecaprenyl phosphate-alpha-L-Ara4N transferase
Mfumv2_0683	183	182	24	24	591	72	72	303	55	55	hypothetical protein
Mfumv2_0684	441	116	37	37	180	53	53	137	59	60	hypothetical protein
Mfumv2_0685	1338	72	70	70	72	64	64	84	111	111	Acid phosphatase
Mfumv2_0686	585	62	26	26	131	48	51	109	61	63	hypothetical protein
Mfumv2_0687	525	174	66	66	203	71	71	271	141	141	Phosphopantetheine adenylyltransferase (EC 2.7.7.3)
Mfumv2_0688	1266	418	382	382	432	364	364	414	520	520	UDP-N-acetylglucosamine 1-carboxyvinyltransferase (EC 2.5.1.7)
Mfumv2_0689	810	121	71	71	139	74	75	509	409	409	Protein-N(5)-glutamine methyltransferase PrmC, methylates polypeptide chain release factors RF1 and RF2
Mfumv2_0690	1137	859	704	705	1202	910	910	1083	1221	1221	Peptide chain release factor 1
Mfumv2_0691	201	778	113	113	1800	241	241	933	186	186	hypothetical protein
Mfumv2_0692	1245	1034	927	930	1231	1021	1021	2582	3188	3188	3-oxoacyl-[acyl-carrier-protein] synthase, KASII (EC 2.3.1.41)
Mfumv2_0693	321	983	227	228	1730	369	370	3145	1001	1001	Acyl carrier protein
Mfumv2_0694	741	241	129	129	265	131	131	1262	924	927	3-oxoacyl-[acyl-carrier protein] reductase (EC 1.1.1.100)
Mfumv2_0695	921	113	75	75	116	71	71	487	444	445	Malonyl CoA-acyl carrier protein transacylase (EC 2.3.1.39)
Mfumv2_0696	678	280	137	137	396	179	179	509	341	342	Endonuclease III (EC 4.2.99.18)
Mfumv2_0697	1305	266	251	251	428	371	372	406	524	525	Ribosomal protein S12p Asp88 (E. coli) methylthiotransferase
Mfumv2_0698	1092	191	151	151	290	211	211	320	347	347	Transcriptional regulator
Mfumv2_0699	2463	194	344	345	240	394	394	258	628	630	Cell division protein FtsK
Mfumv2_0700	636	294	135	135	321	136	136	636	401	401	Manganese superoxide dismutase (EC 1.15.1.1)
Mfumv2_0701	297	159	34	34	253	50	50	540	154	159	hypothetical protein
Mfumv2_0702	525	76	29	29	77	27	27	213	111	111	hypothetical protein
Mfumv2_0703	822	93	55	55	177	97	97	317	258	258	Histidinol-phosphatase (EC 3.1.3.15)
Mfumv2_0704	888	109	70	70	79	46	47	122	107	107	Phytoene synthase (EC 2.5.1.32)
Mfumv2_0705	612	631	279	279	704	287	287	297	180	180	Dephospho-CoA kinase (EC 2.7.1.24)

Mfumv2_0706	1488	440	472	473	700	694	694	973	1436	1436	Transcription termination factor Rho
Mfumv2_0708	150	18	2	2	50	5	5	27	4	4	hypothetical protein
Mfumv2_0709	909	34	22	22	30	18	18	23	21	21	UDP-glucose 4-epimerase (EC 5.1.3.2)
Mfumv2_0710	906	853	555	558	143	85	86	207	181	186	Oligopeptide transport system permease protein OppC (TC 3.A.1.5.1)
Mfumv2_0711	918	112	74	74	139	84	85	269	245	245	Oligopeptide transport system permease protein OppB (TC 3.A.1.5.1)
Mfumv2_0712	267	353	68	68	602	107	107	585	155	155	Ribosome-binding factor A
Mfumv2_0713	2088	862	1298	1300	1081	1503	1503	937	1938	1940	Translation initiation factor 2
Mfumv2_0714	1257	1107	1004	1005	1112	931	931	859	1071	1071	Transcription termination protein NusA
Mfumv2_0715	843	105	64	64	82	46	46	230	192	192	UDP-2,3-diacylglucosamine pyrophosphatase
Mfumv2_0716	927	133	89	89	177	109	109	201	185	185	Oxidoreductase, Gfo/Idh/MocA family
Mfumv2_0717	1182	436	371	372	253	199	199	195	227	229	Lipid-A-disaccharide synthase (EC 2.4.1.182)
Mfumv2_0718	1059	732	560	560	540	381	381	383	402	402	hypothetical protein
Mfumv2_0719	615	894	397	397	937	384	384	859	524	524	RNA polymerase sigma factor RpoE
Mfumv2_0720	558	732	295	295	961	357	357	571	316	316	hypothetical protein
Mfumv2_0721	1086	261	205	205	442	320	320	361	389	389	HtrA protease/chaperone protein
Mfumv2_0722	927	675	451	452	915	565	565	393	360	361	Flagellar motor protein
Mfumv2_0723	186	2263	304	304	1897	235	235	1908	352	352	SSU ribosomal protein S21p
Mfumv2_0724	1101	207	159	165	370	249	271	410	435	448	Alcohol dehydrogenase (EC 1.1.1.1)
Mfumv2_0726	147	19	2	2	0	0	0	14	2	2	hypothetical protein
Mfumv2_0727	186	0	0	0	0	0	0	0	0	0	hypothetical protein
Mfumv2_0728	123	0	0	0	12	1	1	25	3	3	hypothetical protein
Mfumv2_0729	2478	146	262	262	99	163	164	182	446	447	Glycosyltransferase (EC 2.4.1.-)
Mfumv2_0730	162	154	18	18	120	13	13	87	14	14	hypothetical protein
Mfumv2_0732	279	273	0	55	124	0	23	58	0	16	RNA-directed DNA polymerase (Reverse transcriptase)
Mfumv2_0733	492	203	0	72	79	0	26	72	0	35	Retron-type RNA-directed DNA polymerase (EC 2.7.7.49)
Mfumv2_0735	129	11	1	1	0	0	0	0	0	0	hypothetical protein
Mfumv2_0736	3879	677	1894	1898	585	1512	1512	465	1789	1789	hypothetical protein
Mfumv2_0738	348	183	40	46	47	11	11	1904	561	657	hypothetical protein
Mfumv2_0740	849	514	314	315	596	336	337	685	577	577	Glucose-1-phosphate cytidylyltransferase (EC 2.7.7.33)
Mfumv2_0741	996	659	474	474	894	592	593	774	764	764	UDP-glucose 4-epimerase (EC 5.1.3.2)
Mfumv2_0742	546	238	94	94	308	112	112	416	225	225	dTDP-4-dehydrorhamnose 3,5-epimerase (EC 5.1.3.13)
Mfumv2_0743	933	145	98	98	266	162	165	473	432	438	hypothetical protein
Mfumv2_0744	120	138	12	12	375	29	30	218	26	26	hypothetical protein
Mfumv2_0745	1197	239	202	207	171	135	136	349	407	414	L-2-hydroxyglutarate oxidase (EC 1.1.3.15)
Mfumv2_0748	1224	109	96	96	98	77	80	74	88	90	Na+/H+ antiporter
Mfumv2_0749	1137	297	244	244	334	253	253	346	390	390	NADH:flavin oxidoreductase/NADH oxidase
Mfumv2_0750	672	181	88	88	159	71	71	150	100	100	Glutamine amidotransferase, class I
Mfumv2_0751	2001	304	437	439	244	325	325	259	514	514	Hydrogenase-4 component B (EC 1.-.-.-) / Formate hydrogenlyase subunit 3
Mfumv2_0752	951	335	229	230	212	133	134	198	187	187	Formate hydrogenlyase subunit 4
Mfumv2_0753	576	365	151	152	177	68	68	278	159	159	Hydrogenase-4 component E (EC 1.-.-.-)
Mfumv2_0754	1431	239	247	247	135	128	129	196	278	278	Formate hydrogenlyase subunit 3/Multisubunit Na+/H+ antiporter, MnhD subunit
Mfumv2_0755	1533	285	315	316	191	194	195	272	411	414	Formate hydrogenlyase subunit 5
Mfumv2_0756	513	305	113	113	357	122	122	301	152	153	Formate hydrogenlyase subunit 7
Mfumv2_0758	177	0	0	0	25	3	3	34	6	6	hypothetical protein
Mfumv2_0759	1242	52	47	47	33	27	27	27	33	33	hypothetical protein
Mfumv2_0760	345	3576	887	891	1884	433	433	783	268	268	hypothetical protein
Mfumv2_0761	393	958	272	272	611	160	160	354	137	138	hypothetical protein
Mfumv2_0762	192	620	86	86	469	60	60	536	102	102	hypothetical protein
Mfumv2_0763	390	572	161	161	489	127	127	228	88	88	hypothetical protein
Mfumv2_0764	468	228	77	77	106	33	33	142	66	66	hypothetical protein

Mfumv2_0765	1017	131	96	96	66	45	45	88	89	89	Radical SAM domain protein
Mfumv2_0766	306	249	55	55	128	26	26	92	28	28	hypothetical protein
Mfumv2_0767	2142	130	201	201	81	116	116	72	152	152	Membrane-fusion protein, HlyD family
Mfumv2_0768	921	138	92	92	117	72	72	172	157	157	secretion protein HlyD
Mfumv2_0769	2184	96	151	151	56	81	81	91	197	198	cyclic nucleotide-binding protein
Mfumv2_0770	1518	26	29	29	13	13	13	68	103	103	Protein containing domains DUF404, DUF407
Mfumv2_0771	912	26	17	17	28	17	17	41	36	37	hypothetical protein
Mfumv2_0772	2400	57	99	99	48	76	76	66	157	157	hypothetical protein
Mfumv2_0773	858	53	33	33	49	28	28	53	45	45	transglutaminase domain protein
Mfumv2_0774	684	49	24	24	59	26	27	69	47	47	hypothetical protein
Mfumv2_0775	135	164	16	16	289	26	26	60	8	8	hypothetical protein
Mfumv2_0776	147	19	2	2	71	7	7	75	11	11	hypothetical protein
Mfumv2_0777	738	77	41	41	33	16	16	115	83	84	COG2771: DNA-binding HTH domain-containing proteins
Mfumv2_0778	189	81	11	11	48	6	6	59	11	11	hypothetical protein
Mfumv2_0779	681	309	152	152	196	89	89	299	202	202	Two component Transcriptional regulator, Winged helix family protein
Mfumv2_0782	510	68	25	25	74	25	25	47	24	24	hypothetical protein
Mfumv2_0783	909	78	51	51	61	37	37	73	66	66	hypothetical protein
Mfumv2_0784	1626	130	153	153	160	172	173	136	218	219	hypothetical protein
Mfumv2_0785	2076	87	130	130	126	174	174	227	468	468	N-methylhydantoinase A (EC 3.5.2.14)
Mfumv2_0786	1566	186	208	210	123	125	128	210	324	326	N-methylhydantoinase B (EC 3.5.2.14)
Mfumv2_0787	549	45	18	18	79	26	29	121	66	66	hypothetical protein
Mfumv2_0788	3180	58	132	134	99	210	210	147	461	463	Cobalt-zinc-cadmium resistance protein CzcA; Cation efflux system protein CusA
Mfumv2_0789	867	54	34	34	132	76	76	185	159	159	Membrane-fusion protein
Mfumv2_0790	1314	57	54	54	135	118	118	165	215	215	Heavy metal RND efflux outer membrane protein, CzcC family
Mfumv2_0791	1323	48	46	46	65	57	57	114	149	149	Heavy metal RND efflux outer membrane protein, CzcC family
Mfumv2_0792	747	213	115	115	277	138	138	331	245	245	Phosphoglycerate mutase (EC 5.4.2.1)
Mfumv2_0793	1407	49	50	50	62	58	58	85	117	118	putative two-component sensor
Mfumv2_0794	672	157	76	76	150	67	67	218	145	145	two component transcriptional regulator, winged helix family
Mfumv2_0795	1089	303	237	238	376	272	273	796	856	860	Phosphoserine aminotransferase (EC 2.6.1.52)
Mfumv2_0797	798	73	42	42	77	41	41	183	144	145	hypothetical protein
Mfumv2_0798	1065	113	87	87	135	96	96	102	108	108	NADH:flavin oxidoreductase/NADH oxidase
Mfumv2_0799	690	339	168	169	366	168	168	322	220	220	Ribulose-phosphate 3-epimerase (EC 5.1.3.1)
Mfumv2_0800	1404	828	797	840	673	618	629	586	815	816	2-methylcitrate dehydratase (EC 4.2.1.79)
Mfumv2_0802	1404	87	87	88	164	152	153	190	265	265	Selenium-binding protein 1
Mfumv2_0803	627	73	33	33	65	27	27	98	61	61	hypothetical protein
Mfumv2_0804	240	450	78	78	438	70	70	143	34	34	hypothetical protein
Mfumv2_0807	342	170	42	42	167	38	38	221	74	75	hypothetical protein
Mfumv2_0808	621	203	91	91	118	49	49	253	156	156	Aquaporin Z
Mfumv2_0809	1413	129	132	132	109	103	103	83	116	116	alkaline phosphatase D
Mfumv2_0810	324	1872	438	438	2553	550	551	423	136	136	hypothetical protein
Mfumv2_0811	1185	65	56	56	58	46	46	59	69	69	DNA polymerase IV (EC 2.7.7.7)
Mfumv2_0812	117	36	3	3	26	2	2	9	1	1	hypothetical protein
Mfumv2_0813	1005	207	150	150	184	123	123	149	147	148	S-adenosylmethionine:tRNA ribosyltransferase-isomerase (EC 5.-.-.-)
Mfumv2_0814	831	497	298	298	1841	1017	1019	789	650	650	transcriptional regulator, GntR family
Mfumv2_0815	1728	675	843	843	2369	2726	2727	1409	2413	2415	Sulfite reductase [NADPH] hemoprotein beta-component (EC 1.8.1.2)
Mfumv2_0816	444	508	163	163	565	166	167	293	129	129	UspA domain protein
Mfumv2_0817	582	209	88	88	335	130	130	374	216	216	Alkyl hydroperoxide reductase and/or thiol-specific antioxidant family (AhpC/TSA) protein
Mfumv2_0818	336	91	22	22	290	65	65	234	78	78	hypothetical protein
Mfumv2_0819	600	411	177	178	418	167	167	726	431	432	Recombination protein RecR
Mfumv2_0820	309	986	220	220	714	145	147	927	284	284	FIG000557: hypothetical protein co-occurring with RecR

Mfumv2_0821	1701	255	311	313	327	370	371	473	798	798	DNA polymerase III subunits gamma and tau (EC 2.7.7.7)
Mfumv2_0822	150	738	79	80	1121	110	112	934	127	139	hypothetical protein
Mfumv2_0823	453	1152	376	377	1200	362	362	1701	764	764	hypothetical protein
Mfumv2_0824	2166	376	588	588	426	615	615	625	1343	1343	Serine hydroxymethyltransferase (EC 2.1.2.1)
Mfumv2_0825	834	445	268	268	322	179	179	495	406	409	Acetyl-coenzyme A carboxyl transferase beta chain (EC 6.4.1.2)
Mfumv2_0826	231	12	2	2	58	9	9	13	3	3	Competence protein F homolog, phosphoribosyltransferase domain
Mfumv2_0827	183	0	0	0	8	1	1	0	0	0	hypothetical protein
Mfumv2_0828	153	0	0	0	0	0	0	33	5	5	hypothetical protein
Mfumv2_0829	135	0	0	0	0	0	0	37	5	5	hypothetical protein
Mfumv2_0830	147	0	0	0	0	0	0	7	1	1	hypothetical protein
Mfumv2_0831	303	101	21	22	74	15	15	87	26	26	hypothetical protein
Mfumv2_0832	138	451	44	45	620	54	57	314	41	43	hypothetical protein
Mfumv2_0833	147	179	18	19	92	9	9	82	12	12	hypothetical protein
Mfumv2_0835	201	586	82	85	403	53	54	346	69	69	hypothetical protein
Mfumv2_0836	132	21	2	2	11	1	1	23	3	3	hypothetical protein
Mfumv2_0837	732	155	82	82	94	46	46	18	13	13	hypothetical protein
Mfumv2_0838	2823	16	33	33	15	29	29	23	65	65	Outer membrane receptor protein, mostly Fe transport
Mfumv2_0839	588	24	10	10	13	5	5	15	9	9	hypothetical protein
Mfumv2_0840	153	27	2	3	88	9	9	46	7	7	hypothetical protein
Mfumv2_0841	438	38	12	12	69	20	20	28	12	12	Competence protein F homolog, phosphoribosyltransferase domain
Mfumv2_0842	852	67	40	41	79	44	45	46	39	39	CAAX amino terminal protease family
Mfumv2_0843	966	255	177	178	337	217	217	80	77	77	Acetyl-coenzyme A carboxyl transferase alpha chain (EC 6.4.1.2)
Mfumv2_0844	513	135	50	50	143	49	49	57	29	29	6,7-dimethyl-8-ribityllumazine synthase (EC 2.5.1.78)
Mfumv2_0845	129	21	2	2	23	2	2	16	2	2	hypothetical protein
Mfumv2_0846	444	137	44	44	186	54	55	207	89	91	Transcription termination protein NusB
Mfumv2_0847	849	142	87	87	196	111	111	277	232	233	Signal recognition particle receptor protein FtsY (=alpha subunit) (TC 3.A.5.1.1)
Mfumv2_0848	1065	436	334	335	368	260	261	167	175	176	Diaminohydroxyphosphoribosylaminopyrimidine deaminase (EC 3.5.4.26)
Mfumv2_0849	513	19	7	7	35	11	12	71	35	36	Hypoxanthine-guanine phosphoribosyltransferase (EC 2.4.2.8)
Mfumv2_0850	480	49	17	17	47	15	15	71	34	34	hypothetical protein
Mfumv2_0851	1584	29	32	33	38	40	40	42	66	66	Apolipoprotein N-acyltransferase (EC 2.3.1.-) / Copper homeostasis protein CutE
Mfumv2_0852	1527	100	110	110	133	134	135	144	218	218	Peptidyl-prolyl cis-trans isomerase PpiD (EC 5.2.1.8)
Mfumv2_0853	1110	92	73	74	134	99	99	78	86	86	permease YjgP/YjgQ family protein
Mfumv2_0854	336	288	70	70	210	47	47	102	34	34	HNH endonuclease
Mfumv2_0855	591	225	96	96	269	106	106	191	112	112	Phosphoheptose isomerase (EC 5.3.1.-)
Mfumv2_0856	447	232	75	75	238	71	71	250	111	111	Biotin carboxyl carrier protein of acetyl-CoA carboxylase
Mfumv2_0857	117	71	6	6	38	3	3	138	16	16	hypothetical protein
Mfumv2_0858	894	136	88	88	212	126	126	192	170	170	DNA polymerase III delta prime subunit (EC 2.7.7.7)
Mfumv2_0859	633	221	101	101	180	76	76	182	114	114	Thymidylate kinase (EC 2.7.4.9)
Mfumv2_0860	1191	90	77	77	156	124	124	171	202	202	Cysteinyl-tRNA synthetase (EC 6.1.1.16)
Mfumv2_0861	1422	63	65	65	78	74	74	234	330	330	Mercuric ion reductase (EC 1.16.1.1)
Mfumv2_0862	693	64	32	32	93	43	43	207	142	142	hypothetical protein
Mfumv2_0863	783	111	63	63	104	54	54	182	141	141	Enoyl-[acyl-carrier-protein] reductase [NADH] (EC 1.3.1.9)
Mfumv2_0864	195	36	5	5	23	3	3	0	0	0	transposase IS605 OrfB
Mfumv2_0865	375	33	9	9	36	9	9	38	14	14	transposase, IS605 OrfB
Mfumv2_0866	630	1374	237	625	667	102	280	435	128	272	hypothetical protein
Mfumv2_0871	1938	629	879	880	710	915	917	554	1065	1065	hypothetical protein
Mfumv2_0872	795	63	36	36	96	51	51	114	90	90	Biotin operon repressor / Biotin-protein ligase (EC 6.3.4.15)
Mfumv2_0873	816	789	465	465	822	447	447	702	568	568	Similar to Hydroxyacylglutathione hydrolase, but in an organism lacking glutathione biosynthesis
Mfumv2_0874	441	10380	3301	3306	11037	3241	3242	7098	3104	3104	hypothetical protein
Mfumv2_0875	945	100	67	68	92	58	58	236	221	221	Periplasmic solute binding protein

Mfumv2_0876	762	240	132	132	193	98	98	144	109	109	hypothetical protein
Mfumv2_0877	867	65	41	41	74	43	43	70	60	60	Zinc ABC transporter, inner membrane permease protein ZnuB
Mfumv2_0878	630	37	17	17	60	25	25	75	47	47	Phosphoglycolate phosphatase (EC 3.1.3.18)
Mfumv2_0879	1044	133	100	100	160	111	111	120	124	124	hypothetical protein
Mfumv2_0880	1836	136	181	181	163	199	199	146	265	265	5,10-methylenetetrahydrofolate reductase (EC 1.5.1.20) / Homolog of homocysteine-binding domain
Mfumv2_0881	381	160	44	44	213	54	54	217	82	82	peptidase M48, Ste24p(EC:3.4.24.84)
Mfumv2_0882	123	146	13	13	293	24	24	590	72	72	hypothetical protein
Mfumv2_0883	618	45	20	20	112	46	46	62	38	38	hypothetical protein
Mfumv2_0884	1140	52	41	43	84	64	64	65	73	73	hypothetical protein
Mfumv2_0885	771	88	49	49	95	49	49	105	80	80	hypothetical protein
Mfumv2_0886	810	112	195	195	269	145	145	157	126	126	Hydrogenase expression/formation protein hoxQ
Mfumv2_0887	1530	73	81	81	73	74	74	92	140	140	Glucans biosynthesis protein G precursor
Mfumv2_0888	1503	119	129	129	146	146	146	208	309	310	Glucans biosynthesis protein G precursor
Mfumv2_0889	393	67	19	19	84	22	22	174	68	68	hypothetical protein
Mfumv2_0890	2103	69	105	105	47	66	66	88	184	184	Glucans biosynthesis glucosyltransferase H (EC 2.4.1.-)
Mfumv2_0891	480	184	55	55	191	61	61	160	76	76	hydrogenase maturation protease
Mfumv2_0892	342	137	19	19	145	33	33	156	53	53	[NiFe] hydrogenase nickel incorporation protein HybF
Mfumv2_0893	693	102	51	51	130	60	60	486	334	334	Endonuclease V (EC 3.1.21.7)
Mfumv2_0894	303	1243	271	272	1908	384	385	320	96	96	hypothetical protein
Mfumv2_0897	141	29	0	3	0	0	0	14	0	2	hypothetical protein
Mfumv2_0898	156	0	0	0	38	4	4	13	2	2	hypothetical protein
Mfumv2_0899	123	23	2	2	12	1	1	16	2	2	hypothetical protein
Mfumv2_0900	1014	97	70	71	142	92	96	83	83	83	Methyltransferase(EC:2.1.1.-)
Mfumv2_0901	120	104	9	9	350	28	28	2185	260	260	hypothetical protein
Mfumv2_0902	585	178	75	75	213	83	83	290	168	168	Carbonic anhydrase (EC 4.2.1.1)
Mfumv2_0903	120	12	1	1	88	7	7	17	2	2	hypothetical protein
Mfumv2_0904	120	46	4	4	38	3	3	25	3	3	hypothetical protein
Mfumv2_0905	267	47	9	9	67	12	12	45	12	12	hypothetical protein
Mfumv2_0906	441	50	16	16	48	14	14	85	37	37	hypothetical protein
Mfumv2_0907	693	116	57	58	223	103	103	220	149	151	Ribonucleotide reductase of class III (anaerobic), activating protein (EC 1.97.1.4)
Mfumv2_0908	1836	125	166	166	253	310	310	281	510	512	Ribonucleotide reductase of class III (anaerobic), large subunit (EC 1.17.4.2)
Mfumv2_0909	381	185	51	51	378	96	96	299	113	113	hypothetical protein
Mfumv2_0910	1035	135	101	101	191	131	132	202	204	207	Glycosyltransferase
Mfumv2_0912	696	121	61	61	52	24	24	128	88	88	Periplasmic thiol:disulfide interchange protein DsbA
Mfumv2_0913	297	89	19	19	101	20	20	122	36	36	hypothetical protein
Mfumv2_0914	789	223	126	127	190	99	100	148	116	116	metal dependent hydrolase
Mfumv2_0915	1326	108	103	103	216	188	191	183	241	241	RNA polymerase sigma-54 factor RpoN
Mfumv2_0916	222	443	71	71	467	69	69	486	107	107	hypothetical protein
Mfumv2_0917	213	143	22	22	120	16	17	80	17	17	hypothetical protein
Mfumv2_0918	2748	255	503	506	319	579	584	343	933	935	Alanyl-tRNA synthetase (EC 6.1.1.7)
Mfumv2_0919	747	387	209	209	484	240	241	362	268	268	FIG000859: hypothetical protein YebC
Mfumv2_0920	1137	272	221	223	294	223	223	208	234	234	Ribonuclease D (EC 3.1.26.3)
Mfumv2_0921	1188	1223	1049	1049	1249	986	988	1196	1407	1409	Sulfur carrier protein adenylyltransferase ThiF
Mfumv2_0922	306	507	111	112	952	194	194	1032	313	313	ATP-dependent Clp protease adaptor protein ClpS
Mfumv2_0923	510	244	90	90	492	165	167	384	194	194	hypothetical protein
Mfumv2_0924	2460	210	372	373	255	418	418	362	884	884	Glycogen phosphorylase (EC 2.4.1.1)
Mfumv2_0925	1344	532	516	516	542	484	485	439	584	585	hypothetical protein
Mfumv2_0926	120	692	60	60	1076	86	86	538	64	64	hypothetical protein
Mfumv2_0927	138	30	3	3	22	2	2	37	5	5	hypothetical protein
Mfumv2_0930	381	36	10	10	63	16	16	69	26	26	Cupin 2 conserved barrel domain protein

Mfumv2_0931	117	36	3	3	38	3	3	26	3	3	hypothetical protein
Mfumv2_0932	2778	72	144	144	87	161	161	136	373	375	Mg(2+) transport ATPase, P-type (EC 3.6.3.2)
Mfumv2_0933	972	615	430	432	704	455	456	643	619	620	Arabinose 5-phosphate isomerase (EC 5.3.1.13)
Mfumv2_0934	561	1254	507	508	1740	650	650	949	528	528	Translation elongation factor P
Mfumv2_0935	834	297	179	179	450	249	250	424	351	351	beta-lactamase domain protein
Mfumv2_0936	969	320	224	224	392	253	253	826	793	794	Ribose-phosphate pyrophosphokinase (EC 2.7.6.1)
Mfumv2_0937	510	266	98	98	271	92	92	546	276	276	Transcriptional regulator, AsnC family
Mfumv2_0938	1236	274	245	245	411	335	338	756	924	926	Aspartate aminotransferase (EC 2.6.1.1)
Mfumv2_0939	462	1175	392	392	1004	309	309	941	431	431	Amino acid-binding ACT
Mfumv2_0940	1191	524	451	451	782	619	620	820	968	968	S-adenosylmethionine synthetase (EC 2.5.1.6)
Mfumv2_0941	1320	1544	1465	1472	916	805	805	1257	1646	1646	Adenosylhomocysteinase (EC 3.3.1.1)
Mfumv2_0942	459	139	46	46	82	25	25	358	162	163	Cytochrome c family protein
Mfumv2_0943	1305	76	72	72	67	58	58	165	213	214	Twin-arginine translocation pathway signal
Mfumv2_0944	672	163	79	79	176	76	79	149	99	99	ErfK/YbiS/YcfS/YnhG family protein
Mfumv2_0945	192	101	14	14	156	17	20	147	28	28	hypothetical protein
Mfumv2_0946	549	55	22	22	60	22	22	84	46	46	putative methyltransferase
Mfumv2_0947	1278	44	41	41	43	37	37	68	86	86	AsmA family protein
Mfumv2_0948	882	46	29	29	71	42	42	80	69	70	Transcription regulator [contains diacylglycerol kinase catalytic domain]
Mfumv2_0949	426	748	230	230	1339	380	380	1910	806	807	SSU ribosomal protein S12p (S23e)
Mfumv2_0951	471	2349	799	799	1948	611	611	1608	751	751	SSU ribosomal protein S7p (S5e)
Mfumv2_0952	2133	1111	1710	1711	1464	2079	2080	1989	4205	4208	Translation elongation factor G
Mfumv2_0953	306	706	156	156	1266	258	258	2449	743	743	SSU ribosomal protein S10p (S20e)
Mfumv2_0954	708	909	464	465	1444	680	681	866	608	608	LSU ribosomal protein L3p (L3e)
Mfumv2_0955	657	1170	552	555	1341	583	587	1078	692	702	LSU ribosomal protein L4p (L1e)
Mfumv2_0956	285	515	106	106	885	168	168	1023	288	289	LSU ribosomal protein L23p (L23Ae)
Mfumv2_0957	852	907	557	558	1729	981	981	973	821	822	LSU ribosomal protein L2p (L8e)
Mfumv2_0958	276	1184	235	236	1295	238	238	1162	318	318	SSU ribosomal protein S19p (S15e)
Mfumv2_0959	339	1278	313	313	1656	374	374	2064	694	694	LSU ribosomal protein L22p (L17e)
Mfumv2_0960	648	1192	557	558	1893	817	817	1556	1000	1000	SSU ribosomal protein S3p (S3e)
Mfumv2_0961	426	2483	763	764	2203	625	625	3077	1300	1300	LSU ribosomal protein L16p (L10e)
Mfumv2_0962	213	1671	257	257	2418	342	343	2405	508	508	hypothetical protein
Mfumv2_0963	294	2115	449	449	3299	646	646	1691	493	493	SSU ribosomal protein S17p (S11e)
Mfumv2_0964	306	1353	299	299	2586	527	527	2340	709	710	LSU ribosomal protein L14p (L23e)
Mfumv2_0965	315	1147	261	261	3703	777	777	2094	653	654	LSU ribosomal protein L24p (L26e)
Mfumv2_0966	570	782	322	322	1559	592	592	1341	758	758	LSU ribosomal protein L5p (L11e)
Mfumv2_0967	387	762	213	213	1036	267	267	808	310	310	SSU ribosomal protein S8p (S15Ae)
Mfumv2_0968	540	1131	441	441	1357	488	488	1302	697	697	LSU ribosomal protein L6p (L9e)
Mfumv2_0969	354	833	210	213	899	210	212	897	314	315	LSU ribosomal protein L18p (L5e)
Mfumv2_0970	639	1313	606	606	1722	733	733	1577	998	999	SSU ribosomal protein S5p (S2e)
Mfumv2_0971	444	1924	616	617	2776	816	821	3527	1551	1553	LSU ribosomal protein L15p (L27Ae)
Mfumv2_0972	132	273	26	26	671	59	59	795	104	104	hypothetical protein
Mfumv2_0973	1491	656	705	706	922	916	916	643	950	951	Preprotein translocase secY subunit (TC 3.A.5.1.1)
Mfumv2_0974	948	191	131	131	184	116	116	184	173	173	Multidrug resistance protein A
Mfumv2_0975	1953	101	142	142	98	127	128	119	231	231	hypothetical protein
Mfumv2_0976	642	345	160	160	297	127	127	149	95	95	hypothetical protein
Mfumv2_0977	135	2985	291	291	2814	253	253	1404	188	188	hypothetical protein
Mfumv2_0978	1056	1853	2652	2653	3449	2426	2426	2076	2174	2174	Uptake hydrogenase small subunit precursor (EC 1.12.99.6)
Mfumv2_0979	1815	803	1768	1770	2233	2698	2699	1746	3143	3143	Uptake hydrogenase large subunit (EC 1.12.99.6)
Mfumv2_0980	546	388	125	126	328	127	127	580	314	314	Hydrogenase maturation protease (EC 3.4.24.-)
Mfumv2_0981	1245	201	181	181	248	206	206	271	334	334	hypothetical protein

Mfumv2_0982	636	226	104	104	279	118	118	260	164	164	hypothetical protein
Mfumv2_0983	135	51	5	5	100	9	9	30	4	4	hypothetical protein
Mfumv2_0984	234	24	4	4	26	4	4	22	5	5	hypothetical protein
Mfumv2_0985	495	11	4	4	9	3	3	12	6	6	hypothetical protein
Mfumv2_0986	543	214	84	84	138	50	50	132	71	71	Outer membrane receptor protein, mostly Fe transport
Mfumv2_0987	123	45	4	4	37	3	3	57	7	7	hypothetical protein
Mfumv2_0988	117	543	214	214	613	524	524	480	321	322	hypothetical protein
Mfumv2_0989	801	218	515	516	325	588	589	230	381	381	[NiFe] hydrogenase nickel incorporation-associated protein HypB
Mfumv2_0990	711	43	72	73	77	79	79	92	160	162	Nickel transporter UreH
Mfumv2_0991	2340	353	132	132	240	120	120	524	212	213	[NiFe] hydrogenase metallocenter assembly protein HypF
Mfumv2_0992	375	483	44	44	727	60	60	948	195	195	[NiFe] hydrogenase metallocenter assembly protein HypC
Mfumv2_0993	1113	734	386	386	843	538	539	560	1046	1046	[NiFe] hydrogenase metallocenter assembly protein HypD
Mfumv2_0994	1017	576	423	423	742	569	571	660	565	565	[NiFe] hydrogenase metallocenter assembly protein HypE
Mfumv2_0995	1206	364	315	317	281	226	226	263	315	315	Phosphoheptose isomerase 1 (EC 5.3.1.-)
Mfumv2_0996	381	22	6	6	32	8	8	40	15	15	Sodium-dependent transporter
Mfumv2_0998	474	32	11	11	16	5	5	23	11	11	hypothetical protein
Mfumv2_0999	126	0	0	0	0	0	0	0	0	0	hypothetical protein
Mfumv2_1002	129	64	6	6	47	4	4	55	7	7	hypothetical protein
Mfumv2_1003	186	15	2	2	16	2	2	11	2	2	hypothetical protein
Mfumv2_1004	150	0	0	0	40	4	4	27	4	4	hypothetical protein
Mfumv2_1005	366	1313	347	347	1686	411	411	353	128	128	hypothetical protein
Mfumv2_1006	165	176	21	21	255	28	28	251	41	41	hypothetical protein
Mfumv2_1007	777	84	47	47	83	43	43	189	145	146	probable permease of ABC transporter
Mfumv2_1008	747	222	120	120	251	125	125	273	202	202	Methionine ABC transporter ATP-binding protein
Mfumv2_1009	816	244	144	144	322	175	175	315	255	255	Mammalian cell entry related domain protein
Mfumv2_1010	696	338	169	170	354	164	164	287	198	198	2-C-methyl-D-erythritol 4-phosphate cytidylyltransferase (EC 2.7.7.60)
Mfumv2_1011	168	511	62	62	652	73	73	216	36	36	LSU ribosomal protein L33p @ LSU ribosomal protein L33p, zinc-independent
Mfumv2_1012	894	156	101	101	181	108	108	117	103	104	CHAD domain containing protein
Mfumv2_1013	1284	67	62	62	108	92	92	70	89	89	alternate gene name: yzbB
Mfumv2_1014	1719	129	160	160	144	165	165	125	213	213	Glycogen phosphorylase (EC 2.4.1.1)
Mfumv2_1015	852	817	496	503	192	109	109	299	253	253	hypothetical protein
Mfumv2_1016	1392	326	327	328	342	317	317	451	622	622	Fumarate hydratase class II (EC 4.2.1.2)
Mfumv2_1017	522	178	67	67	276	96	96	375	194	194	ADP-heptose synthase (EC 2.7.-.-) / D-glycero-beta-D-manno-heptose 7-phosphate kinase
Mfumv2_1018	609	86	38	38	222	90	90	462	279	279	Phosphoribosylglycinamide formyltransferase (EC 2.1.2.2)
Mfumv2_1019	1353	265	259	259	317	286	286	368	494	494	Exodeoxyribonuclease VII large subunit (EC 3.1.11.6)
Mfumv2_1023	741	80	25	43	34	7	17	27	14	20	hypothetical protein
Mfumv2_1024	1185	2248	1917	1924	3461	2732	2732	6751	7932	7933	Translation elongation factor Tu
Mfumv2_1025	303	1184	259	259	1903	383	384	1900	571	571	Preprotein translocase subunit SecE (TC 3.A.5.1.1)
Mfumv2_1026	603	1022	443	445	1379	554	554	3014	1801	1802	Transcription antitermination protein NusG
Mfumv2_1027	438	1129	357	357	1810	528	528	2074	901	901	LSU ribosomal protein L11p (L12e)
Mfumv2_1028	720	1300	675	676	1512	723	725	2570	1835	1835	LSU ribosomal protein L1p (L10Ae)
Mfumv2_1029	537	737	286	286	682	244	244	1087	578	579	LSU ribosomal protein L10p (P0)
Mfumv2_1030	390	4708	1325	1326	3815	991	991	7005	2709	2709	LSU ribosomal protein L7/L12 (P1/P2)
Mfumv2_1031	3858	1198	3319	3339	1348	3463	3465	1977	7561	7563	DNA-directed RNA polymerase beta subunit (EC 2.7.7.6)
Mfumv2_1032	4173	878	2646	2646	1442	4004	4007	1295	5359	5359	DNA-directed RNA polymerase beta' subunit (EC 2.7.7.6)
Mfumv2_1033	891	311	198	200	389	230	231	369	325	326	Methylenetetrahydrofolate dehydrogenase (NADP+) / Methenyltetrahydrofolate cyclohydrolase
Mfumv2_1034	837	38	23	23	43	24	24	39	32	32	Nucleoside-diphosphate-sugar epimerases
Mfumv2_1035	810	337	196	197	576	311	311	451	362	362	Glutamate 5-kinase (EC 2.7.2.11) / RNA-binding C-terminal domain PUA
Mfumv2_1036	1200	280	243	243	424	339	339	430	511	512	Gamma-glutamyl phosphate reductase (EC 1.2.1.41)
Mfumv2_1037	666	1480	710	712	1350	597	599	495	327	327	hypothetical protein

Mfumv2_1038	1110	44	35	35	28	21	21	44	47	48	hypothetical protein
Mfumv2_1040	462	90	30	30	39	12	12	65	30	30	hypothetical protein
Mfumv2_1041	162	26	3	3	28	3	3	12	2	2	hypothetical protein
Mfumv2_1042	132	10	1	1	0	0	0	8	1	1	hypothetical protein
Mfumv2_1043	807	122	70	71	186	100	100	136	109	109	Undecaprenyl-diphosphatase (EC 3.6.1.27)
Mfumv2_1044	963	308	214	214	438	279	281	268	256	256	D-alanyl-D-alanine carboxypeptidase (EC 3.4.16.4)
Mfumv2_1045	879	1066	676	677	927	541	543	1667	1452	1453	ATP phosphoribosyltransferase (EC 2.4.2.17)
Mfumv2_1046	510	244	90	90	262	89	89	293	148	148	FIG049476: HIT family protein
Mfumv2_1047	1131	61	50	50	54	41	41	37	41	41	DNA polymerase III, delta subunit
Mfumv2_1048	765	183	101	101	285	145	145	194	147	147	Glycosyl transferase, group 2 family protein
Mfumv2_1049	573	172	71	71	204	78	78	169	96	96	Adenylate kinase (EC 2.7.4.3)
Mfumv2_1050	795	341	196	196	312	165	165	170	134	134	putative biopolymer transport protein
Mfumv2_1051	429	255	79	79	504	144	144	259	110	110	Biopolymer transport protein ExbD/TolR
Mfumv2_1052	2316	105	175	175	143	220	220	86	197	197	TPR-domain containing protein
Mfumv2_1053	1026	215	159	159	341	233	233	198	200	201	Outer membrane protein A precursor
Mfumv2_1054	570	136	56	56	79	30	30	53	29	30	hypothetical protein
Mfumv2_1055	129	0	0	0	0	0	0	39	5	5	hypothetical protein
Mfumv2_1056	1791	71	90	92	110	131	131	132	234	234	hypothetical protein
Mfumv2_1057	840	48	29	29	66	37	37	68	57	57	hypothetical protein
Mfumv2_1058	1071	116	90	90	87	62	62	71	75	75	hypothetical protein
Mfumv2_1059	2799	98	198	198	144	265	268	84	234	234	hypothetical protein
Mfumv2_1060	3186	70	159	160	86	183	183	92	290	291	UvrD/REP helicase
Mfumv2_1061	378	150	41	41	119	30	30	584	219	219	hypothetical protein
Mfumv2_1065	486	51	18	18	127	41	41	100	48	48	hypothetical protein
Mfumv2_1066	297	89	19	19	217	43	43	312	92	92	hypothetical protein
Mfumv2_1067	783	152	86	86	215	112	112	174	135	135	Twin-arginine translocation protein TatC
Mfumv2_1068	693	320	160	160	539	249	249	403	274	277	FIG053235: Diacylglucosamine hydrolase like
Mfumv2_1069	195	0	0	0	0	0	0	21	4	4	hypothetical protein
Mfumv2_1070	168	338	41	41	89	10	10	162	27	27	hypothetical protein
Mfumv2_1071	1347	84	82	82	74	66	66	196	254	262	Nicotinate phosphoribosyltransferase (EC 2.4.2.11)
Mfumv2_1072	1464	195	206	206	151	147	147	176	255	255	ATP synthase beta chain (EC 3.6.3.14)
Mfumv2_1073	408	98	29	29	195	53	53	136	55	55	hypothetical protein
Mfumv2_1074	279	1022	205	206	194	36	36	101	28	28	hypothetical protein
Mfumv2_1075	654	72	34	34	85	37	37	65	42	42	ATP synthase A chain (EC 3.6.3.14)
Mfumv2_1076	267	52	10	10	118	21	21	189	50	50	ATP synthase C chain (EC 3.6.3.14)
Mfumv2_1077	747	217	116	117	203	101	101	240	178	178	ATP synthase B chain (EC 3.6.3.14)
Mfumv2_1078	1515	79	86	86	154	155	155	178	267	267	ATP synthase alpha chain (EC 3.6.3.14)
Mfumv2_1079	807	81	47	47	149	80	80	264	211	211	ATP synthase gamma chain (EC 3.6.3.14)
Mfumv2_1080	126	88	8	8	179	15	15	128	16	16	hypothetical protein
Mfumv2_1081	1533	90	100	100	99	101	101	169	257	257	Phosphoenolpyruvate carboxykinase [ATP] (EC 4.1.1.49)
Mfumv2_1082	1029	22	16	16	19	13	13	32	32	33	Glycosyl transferase, group 1
Mfumv2_1083	2202	58	91	93	82	121	121	136	297	297	Glycogen debranching enzyme
Mfumv2_1084	1221	86	76	76	92	75	75	132	160	160	Isocitrate lyase (EC 4.1.3.1)
Mfumv2_1085	1545	80	89	89	153	157	157	204	311	312	Malate synthase (EC 2.3.3.9)
Mfumv2_1086	471	79	27	27	51	16	16	103	48	48	hypothetical protein
Mfumv2_1087	168	82	10	10	27	3	3	30	5	5	hypothetical protein
Mfumv2_1088	1485	129	137	138	186	183	184	136	200	200	Glycolate dehydrogenase (EC 1.1.99.14), subunit GlcD
Mfumv2_1089	1077	85	65	66	79	56	57	116	124	124	Glycolate dehydrogenase (EC 1.1.99.14), FAD-binding subunit GlcE
Mfumv2_1090	1173	67	55	57	90	70	70	130	151	151	Glycolate dehydrogenase (EC 1.1.99.14), iron-sulfur subunit GlcF
Mfumv2_1091	891	489	314	315	452	268	268	530	467	468	Opacity protein or related surface antigen

Mfumv2_1092	1293	75	70	70	106	91	91	111	142	142	Membrane fusion component of tripartite multidrug resistance system
Mfumv2_1093	1413	49	50	50	66	62	62	96	134	134	Outer membrane component of tripartite multidrug resistance system
Mfumv2_1094	1569	51	58	58	83	87	87	48	75	75	Inner membrane component of tripartite multidrug resistance system
Mfumv2_1095	1512	121	132	132	130	130	131	73	110	110	Inner membrane component of tripartite multidrug resistance system
Mfumv2_1096	2370	230	393	393	197	311	311	199	466	468	Xylulose-5-phosphate phosphoketolase (EC 4.1.2.9); Fructose-6-phosphate phosphoketolase (EC 4.1.2.22)
Mfumv2_1097	132	21	2	2	23	2	2	23	3	3	hypothetical protein
Mfumv2_1098	2574	189	351	351	317	544	544	387	987	988	DNA topoisomerase III (EC 5.99.1.2)
Mfumv2_1099	1077	67	52	52	95	68	68	92	98	98	Rossmann fold nucleotide-binding protein Smf possibly involved in DNA uptake
Mfumv2_1101	1032	154	115	115	128	88	88	179	182	183	3-dehydroquinate synthase (EC 4.2.3.4)
Mfumv2_1102	3486	145	366	366	228	528	529	211	730	730	DNA polymerase III alpha subunit (EC 2.7.7.7)
Mfumv2_1103	1902	86	118	118	105	133	133	146	276	276	ATP-dependent DNA helicase UvrD/PcrA
Mfumv2_1104	984	179	127	127	157	103	103	241	235	235	ABC transporter, ATP-binding protein
Mfumv2_1105	798	352	202	203	386	204	205	172	136	136	hypothetical protein
Mfumv2_1106	273	304	59	60	231	42	42	428	116	116	Glutaredoxin
Mfumv2_1107	573	348	144	144	477	182	182	496	281	282	Peptide deformylase (EC 3.5.1.88)
Mfumv2_1108	348	103	26	26	121	28	28	128	44	44	Biopolymer transport protein ExbD/TolR
Mfumv2_1109	120	69	6	6	88	7	7	101	12	12	hypothetical protein
Mfumv2_1110	675	330	161	161	349	157	157	441	295	295	MotA/TolQ/ExbB proton channel family protein
Mfumv2_1111	1218	253	222	223	281	227	228	91	110	110	hypothetical protein
Mfumv2_1112	228	1154	190	190	1139	173	173	2017	456	456	PUR-alpha/beta/gamma DNA/RNA-binding
Mfumv2_1113	1077	450	350	350	484	347	347	1160	1239	1239	Octaprenyl diphosphate synthase (EC 2.5.1.90) / Dimethylallyltransferase (EC 2.5.1.1)
Mfumv2_1114	183	401	53	53	599	73	73	942	171	171	hypothetical protein
Mfumv2_1115	387	190	53	53	186	48	48	565	217	217	Ribonuclease P protein component (EC 3.1.26.5)
Mfumv2_1116	267	166	32	32	202	36	36	389	103	103	Protein YidD
Mfumv2_1117	1776	136	175	175	150	177	177	227	400	400	Inner membrane protein translocase component YidC, long form
Mfumv2_1118	1575	177	201	201	192	201	201	386	602	603	L-aspartate oxidase (EC 1.4.3.16)
Mfumv2_1119	264	84	16	16	199	35	35	661	173	173	transcriptional regulator, HxlR family
Mfumv2_1120	984	34	24	24	639	418	419	273	266	266	Copper-containing nitrite reductase (EC 1.7.2.1)
Mfumv2_1121	420	63	19	19	468	130	131	46	19	19	cytochrome c, class IC
Mfumv2_1122	699	24	12	12	198	89	92	43	30	30	cytochrome c, class I
Mfumv2_1123	624	16	7	7	221	92	92	26	16	16	Multicopper oxidase
Mfumv2_1124	1722	64	79	79	101	113	116	83	142	142	Glycosyl transferase, family 39
Mfumv2_1126	129	0	0	0	35	3	3	16	2	2	hypothetical protein
Mfumv2_1128	147	28	3	3	92	9	9	34	5	5	hypothetical protein
Mfumv2_1129	117	0	0	0	0	0	0	17	2	2	hypothetical protein
Mfumv2_1130	477	87	30	30	94	30	30	97	46	46	hypothetical protein
Mfumv2_1131	465	107	36	36	103	32	32	93	43	43	hypothetical protein
Mfumv2_1132	1446	464	477	485	225	217	217	310	445	445	Isocitrate dehydrogenase [NADP] (EC 1.1.1.42)
Mfumv2_1133	861	106	66	66	185	105	106	151	129	129	hypothetical protein
Mfumv2_1134	429	413	124	128	427	122	122	237	100	101	hypothetical protein
Mfumv2_1135	123	23	2	2	12	1	1	57	7	7	hypothetical protein
Mfumv2_1136	1695	18265	22287	22360	4753	5354	5366	107	180	180	4-alpha-glucanotransferase (amylomaltase) (EC 2.4.1.25)
Mfumv2_1137	213	20	3	3	28	4	4	28	6	6	hypothetical protein
Mfumv2_1138	1179	53	42	45	75	55	59	65	75	76	COG1565: Uncharacterized conserved protein
Mfumv2_1139	123	11	1	1	0	0	0	8	1	1	hypothetical protein
Mfumv2_1140	309	67	15	15	44	9	9	52	16	16	hypothetical protein
Mfumv2_1141	939	233	158	158	309	192	193	279	259	260	DnaJ-class molecular chaperone CbpA
Mfumv2_1142	897	77	50	50	72	43	43	126	112	112	8-oxoguanine DNA glycosylase
Mfumv2_1145	117	36	0	3	0	0	0	43	0	5	hypothetical protein
Mfumv2_1146	189	7	1	1	8	1	1	5	1	1	hypothetical protein

Mfumv2_1147	117	0	0	0	0	0	0	52	6	6	hypothetical protein
Mfumv2_1148	1398	380	384	384	348	324	324	451	625	625	Aminotransferase HpnO, required for aminobacteriohopanetriol
Mfumv2_1149	636	96	44	44	172	73	73	235	148	148	Similar to Hydroxyacylglutathione hydrolase, but in an organism lacking glutathione biosynthesis
Mfumv2_1150	1602	122	141	141	161	171	172	218	346	346	Beta-glucosidase (EC 3.2.1.21)
Mfumv2_1151	1182	199	170	170	250	197	197	240	281	281	Monogalactosyldiacylglycerol synthase
Mfumv2_1152	507	68	25	25	127	43	43	169	85	85	hypothetical protein
Mfumv2_1153	531	180	69	69	192	67	68	247	129	130	hypothetical protein
Mfumv2_1154	789	174	97	99	339	174	178	396	305	310	2-Keto-3-deoxy-D-manno-octulosonate-8-phosphate synthase (EC 2.5.1.55)
Mfumv2_1155	1614	179	209	209	292	314	314	224	358	359	CTP synthase (EC 6.3.4.2)
Mfumv2_1156	765	141	78	78	232	118	118	163	124	124	3-deoxy-manno-octulosonate cytidylyltransferase (EC 2.7.7.38)
Mfumv2_1157	540	82	32	32	53	19	19	140	75	75	D-glycero-D-manno-heptose 1,7-bisphosphate phosphatase (EC 3.1.1.-)
Mfumv2_1158	1020	201	148	148	146	99	99	270	273	273	ADP-heptose synthase (EC 2.7.-.-) / D-glycero-beta-D-manno-heptose 7-phosphate kinase
Mfumv2_1159	864	45	28	28	78	45	45	100	86	86	Chorismate mutase I (EC 5.4.99.5) / Prephenate dehydratase (EC 4.2.1.51) # AroHI/PheAIp/ACT domain
Mfumv2_1160	342	101	25	25	83	19	19	68	23	23	Starvation lipoprotein Slp paralog
Mfumv2_1161	870	53	33	33	85	49	49	326	281	281	N-carbamoylputrescine amidase (3.5.1.53)
Mfumv2_1162	1068	96	74	74	125	89	89	184	195	195	Agmatine deiminase (EC 3.5.3.12)
Mfumv2_1163	552	95	38	38	84	31	31	172	94	94	hypothetical protein
Mfumv2_1164	930	31	21	21	58	36	36	179	165	165	ADP-heptose--lipooligosaccharide heptosyltransferase II (EC 2.4.1.-)
Mfumv2_1165	1155	271	226	226	382	293	294	408	465	467	Cysteine desulfurase (EC 2.8.1.7)
Mfumv2_1166	1173	178	150	151	184	143	144	224	260	260	Cysteine desulfurase (EC 2.8.1.7)
Mfumv2_1167	792	129	74	74	178	92	94	303	238	238	Histidinol-phosphatase (EC 3.1.3.15)
Mfumv2_1168	624	53	24	24	67	28	28	113	70	70	Molybdopterin-guanine dinucleotide biosynthesis protein MobA
Mfumv2_1169	531	136	52	52	130	46	46	198	104	104	Molybdopterin-guanine dinucleotide biosynthesis protein MobB
Mfumv2_1170	1221	105	93	93	171	137	139	215	257	260	Molybdopterin biosynthesis enzyme
Mfumv2_1171	915	62	41	41	79	48	48	102	93	93	hypothetical protein
Mfumv2_1172	651	793	373	373	231	100	100	119	77	77	Cytochrome c family protein
Mfumv2_1173	426	133	41	41	127	36	36	104	44	44	hypothetical protein
Mfumv2_1174	453	193	63	63	139	42	42	165	74	74	Thiol peroxidase, Bcp-type (EC 1.11.1.15)
Mfumv2_1175	423	311	95	95	504	141	142	319	134	134	hypothetical protein
Mfumv2_1176	1551	326	365	365	388	401	401	146	224	225	hypothetical protein
Mfumv2_1177	858	134	83	83	142	81	81	132	112	112	Formate dehydrogenase chain D (EC 1.2.1.2)
Mfumv2_1178	702	140	71	71	118	55	55	59	41	41	Molybdenum ABC transporter, periplasmic molybdenum-binding protein ModA (TC 3.A.1.8.1)
Mfumv2_1179	681	57	28	28	42	19	19	65	44	44	Molybdenum transport system permease protein ModB (TC 3.A.1.8.1)
Mfumv2_1180	1146	36	30	30	39	30	30	44	50	50	molybdate ABC transporter, ATPase subunit
Mfumv2_1181	588	42	18	18	84	33	33	84	49	49	hypothetical protein
Mfumv2_1182	1191	53	46	46	72	56	57	47	56	56	transporter, putative
Mfumv2_1183	1836	3757	4981	4982	5660	6919	6922	4986	9075	9077	Methanol dehydrogenase large subunit protein (EC 1.1.99.8)
Mfumv2_1184	873	704	441	444	714	414	415	1630	1411	1411	extracellular solute-binding protein family 3
Mfumv2_1185	852	1388	852	854	1188	674	674	1214	1025	1026	hypothetical protein
Mfumv2_1186	369	1264	337	337	1615	397	397	1610	589	589	Pterin-4-alpha-carbinolamine dehydratase (EC 4.2.1.96)
Mfumv2_1187	159	496	57	57	274	29	29	1560	246	246	hypothetical protein
Mfumv2_1190	771	329	182	183	166	83	85	272	208	208	hypothetical protein
Mfumv2_1191	897	1959	1266	1269	2221	1326	1327	2187	1945	1945	2-hydroxy-3-oxopropionate reductase (EC 1.1.1.60)
Mfumv2_1192	1386	354	354	354	575	531	531	527	724	724	Dihydrolipoamide dehydrogenase (EC 1.8.1.4)
Mfumv2_1193	1221	684	603	603	505	410	411	999	1209	1209	Dihydrolipoamide acetyltransferase component of pyruvate dehydrogenase complex (EC 2.3.1.12)
Mfumv2_1194	135	0	0	0	22	2	2	67	9	9	hypothetical protein
Mfumv2_1195	1701	70	86	86	95	108	108	149	251	251	Tetratricopeptide TPR_2 repeat protein
Mfumv2_1196	978	541	379	382	827	538	539	1358	1315	1317	Pyruvate dehydrogenase E1 component beta subunit (EC 1.2.4.1)
Mfumv2_1197	1074	900	696	698	1204	861	861	695	740	740	Pyruvate dehydrogenase E1 component alpha subunit (EC 1.2.4.1)
Mfumv2_1198	1365	68	67	67	93	85	85	83	112	112	aminotransferase class-III

Mfumv2_1203	327	76	18	18	78	17	17	204	66	66	hypothetical protein
Mfumv2_1204	306	195	43	43	123	25	25	152	46	46	hypothetical protein
Mfumv2_1205	567	689	281	282	532	199	201	647	363	364	hypothetical protein
Mfumv2_1206	900	866	561	563	1054	631	632	1759	1569	1570	hypothetical protein
Mfumv2_1207	204	828	122	122	1016	138	138	1335	269	270	hypothetical protein
Mfumv2_1209	252	912	165	166	1787	294	300	948	237	237	SSU ribosomal protein S18p @ SSU ribosomal protein S18p, zinc-independent
Mfumv2_1210	684	615	304	304	777	350	354	1325	899	899	hypothetical protein
Mfumv2_1211	1644	89	106	106	88	96	96	104	169	169	Glucose-6-phosphate isomerase (EC 5.3.1.9)
Mfumv2_1212	114	12	1	1	26	2	2	18	2	2	hypothetical protein
Mfumv2_1213	138	20	2	2	54	5	5	0	0	0	hypothetical protein
Mfumv2_1214	2829	155	316	316	244	457	459	192	538	538	Malto-oligosyltrehalose synthase (EC 5.4.99.15)
Mfumv2_1215	633	140	37	64	33	5	14	107	33	67	transposase
Mfumv2_1218	1719	120	149	149	137	157	157	181	308	308	Malto-oligosyltrehalose trehalohydrolase (EC 3.2.1.141)
Mfumv2_1219	3408	217	533	535	244	553	555	220	741	742	Trehalose synthase (EC 5.4.99.16)
Mfumv2_1220	132	42	4	4	23	2	2	31	4	4	hypothetical protein
Mfumv2_1221	1395	58	58	58	75	68	70	83	115	115	sigma-54 dependent transcriptional regulator/response regulator
Mfumv2_1222	1446	44	46	46	88	85	85	98	141	141	histidine kinase
Mfumv2_1223	1965	105	149	149	129	169	169	171	332	333	1,4-alpha-glucan (glycogen) branching enzyme, GH-13-type (EC 2.4.1.18)
Mfumv2_1224	1314	44	42	42	78	68	68	88	115	115	Manganese transport protein MntH
Mfumv2_1225	714	240	124	124	210	100	100	230	163	163	transcriptional repressor
Mfumv2_1226	2745	114	226	226	225	409	412	268	729	729	Aconitate hydratase (EC 4.2.1.3)
Mfumv2_1227	579	41	17	17	75	29	29	85	48	49	alpha/beta hydrolase superfamily protein
Mfumv2_1228	1590	47	54	54	52	55	55	110	173	173	Oligopeptide ABC transporter, periplasmic oligopeptide-binding protein OppA (TC 3.A.1.5.1)
Mfumv2_1229	321	190	44	44	281	60	60	386	122	123	Preprotein translocase subunit SecG (TC 3.A.5.1.1)
Mfumv2_1230	768	206	114	114	403	206	206	483	368	368	Triosephosphate isomerase (EC 5.3.1.1)
Mfumv2_1231	1215	224	197	197	445	360	360	731	881	881	Phosphoglycerate kinase (EC 2.7.2.3)
Mfumv2_1232	1044	318	240	240	640	445	445	927	960	960	NAD-dependent glyceraldehyde-3-phosphate dehydrogenase (EC 1.2.1.12)
Mfumv2_1233	144	0	0	0	42	4	4	77	11	11	hypothetical protein
Mfumv2_1234	954	29	20	20	72	46	46	78	74	74	Peptide methionine sulfoxide reductase MsrB (EC 1.8.4.12) / MsrA (EC 1.8.4.11)
Mfumv2_1235	675	51	24	25	118	53	53	140	94	94	hypothetical protein
Mfumv2_1236	315	84	19	19	167	35	35	106	33	33	Periplasmic divalent cation tolerance protein CutA
Mfumv2_1237	171	73	9	9	18	2	2	59	10	10	hypothetical protein
Mfumv2_1238	1107	263	209	210	454	334	335	362	397	397	tRNA-specific 2-thiouridylase MnmA
Mfumv2_1239	1263	386	351	352	664	559	559	600	751	751	ATP-dependent Clp protease ATP-binding subunit ClpX
Mfumv2_1240	660	789	376	376	830	363	365	1005	658	658	ATP-dependent Clp protease proteolytic subunit (EC 3.4.21.92)
Mfumv2_1241	1332	313	300	301	406	360	360	351	464	464	Cell division trigger factor (EC 5.2.1.8)
Mfumv2_1242	195	703	99	99	670	87	87	305	59	59	hypothetical protein
Mfumv2_1243	933	251	169	169	351	218	218	267	247	247	Thioredoxin reductase (EC 1.8.1.9)
Mfumv2_1244	1311	186	176	176	216	189	189	339	440	441	hypothetical protein
Mfumv2_1245	1185	293	250	251	220	174	174	353	415	415	Succinyl-CoA ligase [ADP-forming] beta chain (EC 6.2.1.5)
Mfumv2_1246	891	357	230	230	431	256	256	585	516	517	Succinyl-CoA ligase [ADP-forming] alpha chain (EC 6.2.1.5)
Mfumv2_1247	2073	250	375	375	372	512	513	515	1058	1058	5-Enolpyruvylshikimate-3-phosphate synthase (EC 2.5.1.19) / Cytidylate kinase (EC 2.7.4.14)
Mfumv2_1248	282	987	200	201	1203	226	226	1069	299	299	hypothetical protein
Mfumv2_1249	180	215	28	28	125	15	15	583	104	104	hypothetical protein
Mfumv2_1250	441	63	20	20	10	3	3	46	20	20	hypothetical protein
Mfumv2_1251	645	21	10	10	5	2	2	33	21	21	hypothetical protein
Mfumv2_1252	648	30	14	14	39	17	17	149	95	96	1-acyl-sn-glycerol-3-phosphate acyltransferase (EC 2.3.1.51)
Mfumv2_1253	279	412	82	83	613	114	114	578	160	160	Fe2+ transport system protein A
Mfumv2_1255	2118	108	165	165	245	345	346	199	417	417	Ferrous iron transport protein B
Mfumv2_1256	1677	1787	2163	2165	1922	2147	2147	1762	2930	2930	SSU ribosomal protein S1p

Mfumv2_1257	255	271	50	50	171	29	29	63	16	16	hypothetical protein
Mfumv2_1258	156	328	37	37	395	41	41	142	22	22	hypothetical protein
Mfumv2_1259	483	192	67	67	155	50	50	63	30	30	Cytochrome c oxidase (B(O/a)3-type) chain II (EC 1.9.3.1)
Mfumv2_1260	1686	1087	1320	1324	515	576	578	507	841	847	Cytochrome c oxidase (B(O/a)3-type) chain I (EC 1.9.3.1)
Mfumv2_1261	150	0	0	0	60	6	6	13	2	2	hypothetical protein
Mfumv2_1262	1053	164	125	125	207	145	145	213	222	222	Opacity protein or related surface antigen
Mfumv2_1268	783	37	21	21	48	25	25	43	33	33	hypothetical protein
Mfumv2_1269	183	159	21	21	115	14	14	127	23	23	hypothetical protein
Mfumv2_1270	1263	109	97	99	140	118	118	192	240	241	L-sorbosone dehydrogenase
Mfumv2_1271	510	315	116	116	365	124	124	259	131	131	PEBP family protein
Mfumv2_1272	495	84	30	30	143	47	47	122	60	60	hypothetical protein
Mfumv2_1273	321	250	58	58	393	84	84	320	102	102	hypothetical protein
Mfumv2_1274	279	854	172	172	1195	222	222	741	205	205	hypothetical protein
Mfumv2_1278	777	549	308	308	375	193	194	360	277	277	Putative bacterial haemoglobin
Mfumv2_1279	654	470	128	222	560	127	244	204	71	132	Ribonucleotide reductase of class Ia (aerobic), alpha subunit (EC 1.17.4.1)
Mfumv2_1282	1056	218	166	166	670	470	471	384	402	402	Ribonucleotide reductase of class Ia (aerobic), beta subunit (EC 1.17.4.1)
Mfumv2_1283	1347	201	196	196	468	420	420	301	402	402	Diaminopimelate decarboxylase (EC 4.1.1.20)
Mfumv2_1284	531	289	110	111	447	158	158	446	235	235	Isochorismatase (EC 3.3.2.1)
Mfumv2_1285	381	251	69	69	319	81	81	267	101	101	Nitrogen regulatory protein P-II
Mfumv2_1286	429	77	24	24	161	46	46	106	45	45	UPF0047 protein Bsu YugU
Mfumv2_1287	915	45	30	30	71	43	43	140	127	127	Ribokinase (EC 2.7.1.15)
Mfumv2_1288	243	285	50	50	686	111	111	353	85	85	conserved hypothetical protein
Mfumv2_1289	444	1048	335	336	166	49	49	186	82	82	OsmC-like protein
Mfumv2_1290	363	84	22	22	112	26	27	122	44	44	lipoprotein, RlpA family
Mfumv2_1291	660	61	28	29	93	41	41	127	82	83	hypothetical protein
Mfumv2_1292	804	79	46	46	58	31	31	102	81	81	Putative bacterial haemoglobin
Mfumv2_1293	2424	61	106	107	26	42	42	26	61	62	Outer membrane receptor protein, mostly Fe transport
Mfumv2_1294	1302	183	172	172	42	35	36	29	38	38	Nitrate/nitrite transporter
Mfumv2_1295	360	162	42	42	13	3	3	25	9	9	nitrite reductase [NAD(P)H], small subunit(EC:1.7.1.4)
Mfumv2_1296	2442	404	709	712	26	43	43	26	63	63	Nitrite reductase [NAD(P)H] large subunit (EC 1.7.1.4)
Mfumv2_1297	2151	71	111	111	39	56	56	27	58	58	Assimilatory nitrate reductase large subunit (EC:1.7.99.4)
Mfumv2_1298	126	0	0	0	0	0	0	0	0	0	hypothetical protein
Mfumv2_1299	1083	35	27	27	15	11	11	37	40	40	Nitrate ABC transporter, nitrate-binding protein
Mfumv2_1300	924	76	51	51	91	56	56	26	24	24	transcriptional regulator, LysR family
Mfumv2_1301	843	182	111	111	249	140	140	153	128	128	Phosphosulfolactate synthase (EC 4.4.1.19)
Mfumv2_1302	504	1904	689	693	346	115	116	200	99	100	hypothetical protein
Mfumv2_1303	579	175	73	73	83	32	32	247	142	142	hypothetical protein
Mfumv2_1304	678	57	28	28	31	14	14	430	289	289	Periplasmic protein TonB
Mfumv2_1305	132	262	25	25	182	16	16	31	4	4	hypothetical protein
Mfumv2_1306	1434	136	141	141	166	159	159	127	179	180	Aldehyde decarbonylase
Mfumv2_1307	189	37	5	5	24	3	3	16	3	3	hypothetical protein
Mfumv2_1308	1428	45	46	46	36	34	34	46	65	65	hypothetical protein
Mfumv2_1309	123	0	0	0	24	2	2	8	1	1	hypothetical protein
Mfumv2_1310	1020	257	189	189	318	216	216	220	223	223	Methyltransferase type 12
Mfumv2_1312	192	555	75	77	375	43	48	446	81	85	hypothetical protein
Mfumv2_1314	144	77	8	8	83	8	8	203	29	29	hypothetical protein
Mfumv2_1315	156	80	8	9	19	1	2	26	3	4	hypothetical protein
Mfumv2_1316	654	78	37	37	96	42	42	142	92	92	phosphoribosyltransferase
Mfumv2_1317	1011	175	128	128	221	149	149	242	243	243	Porphobilinogen synthase (EC 4.2.1.24)
Mfumv2_1318	2538	120	219	220	199	336	336	224	565	565	Glutamate-ammonia-ligase adenylyltransferase (EC 2.7.7.42)

Mfumv2_1319	924	291	192	194	177	109	109	188	172	172	Site-specific recombinase XerD
Mfumv2_1320	705	61	31	31	119	56	56	216	151	151	Nucleotidyl transferase
Mfumv2_1321	591	82	35	35	76	30	30	96	56	56	Nucleoside 5-triphosphatase RdgB (dHAPTP, dITP, XTP-specific) (EC 3.6.1.15)
Mfumv2_1322	270	10	2	2	72	13	13	34	9	9	hypothetical protein
Mfumv2_1323	114	24	2	2	13	1	1	9	1	1	hypothetical protein
Mfumv2_1324	219	114	18	18	82	12	12	41	9	9	hypothetical protein
Mfumv2_1326	144	250	25	26	104	6	10	70	4	10	hypothetical protein
Mfumv2_1327	213	52	0	8	14	0	2	5	0	1	hypothetical protein
Mfumv2_1329	279	0	0	0	5	1	1	14	4	4	hypothetical protein
Mfumv2_1331	1086	89	70	70	69	49	50	100	108	108	Transaldolase (EC 2.2.1.2)
Mfumv2_1332	666	195	94	94	440	195	195	215	142	142	FIG137478: Hypothetical protein
Mfumv2_1333	282	1910	389	389	1815	340	341	1205	337	337	DNA-directed RNA polymerase omega subunit (EC 2.7.7.6)
Mfumv2_1334	459	724	240	240	1138	348	348	780	355	355	tmRNA-binding protein SmpB
Mfumv2_1335	234	36	6	6	83	13	13	73	17	17	hypothetical protein
Mfumv2_1336	177	47	6	6	51	6	6	51	9	9	hypothetical protein
Mfumv2_1340	1512	218	237	238	323	325	325	251	377	377	IMP cyclohydrolase (EC3.5.4.10)/Phosphoribosylaminoimidazolecarboxamide formyltransferase (EC2.1.2.3)
Mfumv2_1341	714	372	192	192	515	245	245	302	214	214	hypothetical protein
Mfumv2_1342	711	119	61	61	116	55	55	261	184	184	Ubiquinone/menaquinone biosynthesis methyltransferase UbiE (EC 2.1.1.-)
Mfumv2_1343	546	289	113	114	324	118	118	597	323	323	hypothetical protein
Mfumv2_1344	468	396	134	134	658	205	205	955	443	443	Fe-S metabolism associated SufE
Mfumv2_1345	855	721	444	445	1082	616	616	907	767	769	Thiosulfate sulfurtransferase, rhodanese (EC 2.8.1.1)
Mfumv2_1346	963	216	150	150	262	168	168	250	239	239	Biotin synthase (EC 2.8.1.6)
Mfumv2_1348	1455	47	48	49	54	52	52	64	91	92	two-component system sensor protein
Mfumv2_1349	1224	35	31	31	101	82	82	100	121	121	two-component system regulatory protein
Mfumv2_1350	816	20	12	12	50	27	27	41	32	33	Opacity protein or related surface antigen
Mfumv2_1351	336	742	180	180	1189	266	266	486	162	162	Thioredoxin
Mfumv2_1352	858	302	187	187	411	234	235	390	331	332	hypothetical protein
Mfumv2_1353	1809	201	261	262	313	377	377	234	420	420	Cell division protein FtsH (EC 3.4.24.-)
Mfumv2_1354	792	101	58	58	226	119	119	241	188	189	Uracil-DNA glycosylase, family 4
Mfumv2_1355	972	471	331	331	1024	663	663	1477	1423	1424	Ribosomal large subunit pseudouridine synthase D (EC 4.2.1.70)
Mfumv2_1356	276	3135	624	625	8067	1482	1483	2474	677	677	Ferredoxin
Mfumv2_1357	114	61	5	5	132	10	10	80	9	9	hypothetical protein
Mfumv2_1358	645	125	58	58	149	64	64	161	102	103	Dethiobiotin synthetase (EC 6.3.3.3)
Mfumv2_1359	1428	248	255	256	307	292	292	177	251	251	Nicotinamide mononucleotide adenylyltransferase
Mfumv2_1360	651	121	57	57	85	37	37	195	126	126	Pyridoxamine 5'-phosphate oxidase (EC 1.4.3.5)
Mfumv2_1361	474	1972	675	675	2116	668	668	1904	894	895	Cytochrome c oxidase polypeptide II (EC 1.9.3.1)
Mfumv2_1362	129	1953	182	182	931	79	80	946	120	121	hypothetical protein
Mfumv2_1363	198	252	36	36	106	14	14	637	125	125	hypothetical protein
Mfumv2_1364	192	50	7	7	31	4	4	58	11	11	hypothetical protein
Mfumv2_1365	216	19	3	3	70	10	10	140	30	30	hypothetical protein
Mfumv2_1369	744	67	36	36	42	21	21	47	35	35	hypothetical protein
Mfumv2_1370	657	36	17	17	30	13	13	34	22	22	hypothetical protein
Mfumv2_1371	660	27	13	13	25	11	11	35	23	23	hypothetical protein
Mfumv2_1372	576	231	96	96	138	53	53	33	19	19	hypothetical protein
Mfumv2_1374	399	28	8	8	68	18	18	33	13	13	hypothetical protein
Mfumv2_1375	558	45	18	18	124	44	46	58	32	32	hypothetical protein
Mfumv2_1376	903	123	79	80	96	56	58	49	44	44	hypothetical protein
Mfumv2_1377	174	64	8	8	78	9	9	214	37	37	hypothetical protein
Mfumv2_1378	984	62	44	44	56	36	37	77	75	75	hypothetical protein
Mfumv2_1380	246	23	4	4	18	3	3	20	5	5	hypothetical protein

Mfumv2_1381	918	33	22	22	34	20	21	27	25	25	ATPase, AAA family
Mfumv2_1384	129	32	3	3	0	0	0	0	0	0	hypothetical protein
Mfumv2_1385	129	64	6	6	0	0	0	55	7	7	hypothetical protein
Mfumv2_1386	1869	59	79	79	54	67	67	44	82	82	hypothetical protein
Mfumv2_1387	633	85	39	39	50	21	21	43	27	27	hypothetical protein
Mfumv2_1388	243	11	2	2	12	2	2	12	3	3	hypothetical protein
Mfumv2_1389	393	25	7	7	23	6	6	23	9	9	hypothetical protein
Mfumv2_1390	2130	16	25	25	16	23	23	27	56	56	hypothetical protein
Mfumv2_1391	660	23	11	11	9	4	4	12	8	8	hypothetical protein
Mfumv2_1393	714	35	18	18	38	18	18	47	33	33	hypothetical protein
Mfumv2_1394	597	30	13	13	33	13	13	68	40	40	hypothetical protein
Mfumv2_1395	702	189	95	96	222	104	104	96	67	67	hypothetical protein
Mfumv2_1396	927	8697	5818	5823	8193	5059	5059	3039	2792	2794	hypothetical protein
Mfumv2_1397	2058	20	29	29	15	21	21	26	53	53	type II secretion system protein E
Mfumv2_1398	789	5	3	3	10	5	5	5	4	4	hypothetical protein
Mfumv2_1399	972	16	11	11	15	10	10	18	17	17	hypothetical protein
Mfumv2_1400	1203	31	27	27	21	17	17	33	39	39	Type IV secretory pathway, VirB10 component
Mfumv2_1401	903	6	4	4	18	11	11	12	11	11	hypothetical protein
Mfumv2_1402	1596	25	29	29	45	48	48	45	71	71	Chromosome (plasmid) partitioning protein ParB / Stage 0 sporulation protein J
Mfumv2_1403	897	795	513	515	532	318	318	280	249	249	RNA polymerase sigma factor RpoD
Mfumv2_1404	1368	45	42	44	48	43	44	30	41	41	hypothetical protein
Mfumv2_1405	765	63	34	35	65	33	33	111	84	84	hypothetical protein
Mfumv2_1406	795	21	12	12	30	16	16	47	37	37	hypothetical protein
Mfumv2_1407	345	28	7	7	35	8	8	6	2	2	hypothetical protein
Mfumv2_1408	459	42	14	14	62	19	19	31	14	14	hypothetical protein
Mfumv2_1409	408	7	2	2	11	3	3	15	6	6	hypothetical protein
Mfumv2_1410	2664	45	87	87	34	60	60	50	132	132	hypothetical protein
Mfumv2_1411	2478	43	77	77	36	60	60	22	53	53	hypothetical protein
Mfumv2_1412	390	32	9	9	85	22	22	34	13	13	Outer membrane protein or related peptidoglycan-associated (lipo)protein
Mfumv2_1413	213	7	1	1	0	0	0	24	5	5	hypothetical protein
Mfumv2_1414	783	21	12	12	33	17	17	75	58	58	hypothetical protein
Mfumv2_1415	624	659	297	297	830	345	345	367	227	227	hypothetical protein
Mfumv2_1416	1392	118	119	119	175	161	162	199	273	275	Dihydrolipoamide dehydrogenase (EC 1.8.1.4)
Mfumv2_1417	150	28	3	3	30	2	3	7	1	1	hypothetical protein
Mfumv2_1418	117	0	0	0	0	0	0	26	3	3	hypothetical protein
Mfumv2_1419	345	630	156	157	557	128	128	497	170	170	Nitrogen regulatory protein P-II
Mfumv2_1420	1434	671	695	695	672	642	642	2075	2950	2950	Glutamine synthetase type I (EC 6.3.1.2)
Mfumv2_1421	129	526	49	49	489	42	42	907	116	116	hypothetical protein
Mfumv2_1422	1203	55	48	48	61	49	49	92	110	110	glycosyl transferase, group 1
Mfumv2_1423	768	43	24	24	57	29	29	84	63	64	Phosphoesterase, PA-phosphatase related
Mfumv2_1424	1203	140	120	122	220	174	176	226	270	270	Cysteine desulfurase (EC 2.8.1.7)
Mfumv2_1425	798	345	199	199	348	184	185	186	147	147	hypothetical protein
Mfumv2_1426	354	278	71	71	534	126	126	464	163	163	histidine triad (HIT) protein
Mfumv2_1427	477	601	206	207	746	235	237	271	127	128	Ribonucleotide reductase transcriptional regulator NrdR
Mfumv2_1428	453	83	27	27	89	27	27	169	76	76	hypothetical protein
Mfumv2_1429	192	865	120	120	1001	128	128	709	135	135	hypothetical protein
Mfumv2_1430	1044	195	147	147	250	174	174	556	576	576	Phosphate:acyl-ACP acyltransferase PlsX
Mfumv2_1431	1074	358	277	278	665	475	476	489	520	521	3-oxoacyl-[acyl-carrier-protein] synthase, KASIII (EC 2.3.1.41)
Mfumv2_1432	1089	79	62	62	103	75	75	84	91	91	peptidase M42 family protein
Mfumv2_1433	1455	236	248	248	448	434	434	353	509	509	Amidophosphoribosyltransferase (EC 2.4.2.14)

Mfumv2_1434	1014	111	81	81	179	121	121	154	155	155	Phosphoribosylformylglycinamidine cyclo-ligase (EC 6.3.3.1)
Mfumv2_1436	114	12	0	1	0	0	0	18	0	2	hypothetical protein
Mfumv2_1438	123	11	1	1	0	0	0	8	1	1	hypothetical protein
Mfumv2_1439	138	10	1	1	0	0	0	0	0	0	hypothetical protein
Mfumv2_1440	204	869	123	128	515	69	70	811	162	164	hypothetical protein
Mfumv2_1442	129	642310	59822	59844	581385	49945	49955	556268	71155	71158	hypothetical protein
Mfumv2_1443	144	96	0	10	31	0	3	77	0	11	hypothetical protein
Mfumv2_1449	585	464	196	196	418	163	163	207	120	120	Hemoglobin-like protein HbO
Mfumv2_1450	492	65	23	23	262	86	86	178	87	87	HNH endonuclease family protein
Mfumv2_1451	2805	168	341	341	193	361	361	181	501	503	hypothetical protein
Mfumv2_1452	648	75	35	35	83	36	36	145	93	93	hypothetical protein
Mfumv2_1453	372	141	38	38	153	38	38	73	27	27	hypothetical protein
Mfumv2_1454	132	189	18	18	250	22	22	191	25	25	hypothetical protein
Mfumv2_1455	687	189	93	94	153	70	70	649	442	442	3-methyladenine DNA glycosylase
Mfumv2_1456	228	729	119	120	461	70	70	845	191	191	NAD-dependent formate dehydrogenase delta subunit
Mfumv2_1457	2973	487	1045	1045	558	1104	1105	1355	3996	3996	NAD-dependent formate dehydrogenase alpha subunit
Mfumv2_1458	1569	252	286	286	273	285	285	1346	2090	2094	NAD-dependent formate dehydrogenase beta subunit
Mfumv2_1459	459	220	73	73	239	72	73	989	450	450	NAD-dependent formate dehydrogenase gamma subunit
Mfumv2_1460	1233	52	45	46	55	44	45	96	117	117	Surface antigen gene
Mfumv2_1461	897	621	402	402	825	492	493	641	567	570	Coenzyme PQQ synthesis protein B
Mfumv2_1462	786	787	446	447	1213	635	635	554	431	432	Coenzyme PQQ synthesis protein C
Mfumv2_1463	273	330	64	65	687	125	125	362	98	98	Coenzyme PQQ synthesis protein D
Mfumv2_1464	1023	562	415	415	1048	713	714	421	427	427	Coenzyme PQQ synthesis protein E
Mfumv2_1465	204	2199	323	324	4607	626	626	1958	396	396	hypothetical protein
Mfumv2_1466	3579	497	1284	1285	513	1224	1224	375	1329	1330	Indolepyruvate ferredoxin oxidoreductase, alpha and beta subunits
Mfumv2_1467	1374	331	328	328	409	374	374	435	593	593	Glycyl-tRNA synthetase (EC 6.1.1.14)
Mfumv2_1468	2190	52	82	82	75	109	109	69	149	149	hypothetical protein
Mfumv2_1469	876	76	48	48	98	57	57	99	86	86	Endonuclease IV (EC 3.1.21.2)
Mfumv2_1470	345	193	48	48	261	60	60	1441	493	493	glutaredoxin
Mfumv2_1471	2070	27	41	41	17	23	23	16	33	33	TonB-dependent receptor protein
Mfumv2_1477	213	72	6	11	49	2	7	66	7	14	hypothetical protein
Mfumv2_1478	708	133	68	68	197	93	93	188	132	132	Uracil-DNA glycosylase, family 5
Mfumv2_1479	468	364	123	123	340	106	106	276	128	128	Cytosine deaminase (EC 3.5.4.1)
Mfumv2_1481	1239	170	152	152	279	230	230	345	423	424	Threonine synthase (EC 4.2.3.1)
Mfumv2_1482	291	866	182	182	1166	226	226	977	282	282	FIG038648: MoaD and/or ThiS families
Mfumv2_1483	243	501	88	88	890	144	144	652	157	157	hypothetical protein
Mfumv2_1484	324	11568	2683	2707	2076	446	448	949	305	305	hypothetical protein
Mfumv2_1485	1842	644	856	857	853	1046	1046	826	1507	1508	Transcription elongation factor GreA
Mfumv2_1486	654	42	20	20	60	26	26	65	42	42	Transcriptional regulator, ArsR family
Mfumv2_1487	1596	874	1008	1008	744	791	791	986	1560	1560	D-3-phosphoglycerate dehydrogenase (EC 1.1.1.95)
Mfumv2_1488	330	2312	551	551	2921	642	642	1366	446	447	hypothetical protein
Mfumv2_1489	375	834	226	226	1173	293	293	436	162	162	probable uroporphyrin-III c-methyltransferase (EC 2.1.1.107)
Mfumv2_1490	705	261	133	133	200	93	94	349	244	244	hypothetical protein
Mfumv2_1491	603	604	263	263	722	290	290	888	531	531	Phosphoglycerate mutase (EC 5.4.2.1)
Mfumv2_1492	2028	1813	2655	2656	2819	3806	3808	2777	5585	5585	Transketolase (EC 2.2.1.1)
Mfumv2_1493	1008	853	620	621	1391	933	934	3104	3102	3103	Uridine kinase (EC 2.7.1.48)
Mfumv2_1494	951	2351	1612	1615	2824	1789	1789	12032	11346	11347	probable RuBisCo-expression protein CbbX
Mfumv2_1495	417	3456	1041	1041	6826	1894	1896	8520	3521	3523	Ribulose bisphosphate carboxylase small chain (EC 4.1.1.39)
Mfumv2_1496	1464	4028	4255	4259	7576	7387	7388	6419	9318	9319	Ribulose bisphosphate carboxylase large chain (EC 4.1.1.39)
Mfumv2_1497	1344	152	147	148	178	159	159	182	243	243	hypothetical protein

Mfumv2_1498	333	553	133	133	577	128	128	2722	899	899	LSU ribosomal protein L21p
Mfumv2_1499	258	521	97	97	855	147	147	2767	708	708	LSU ribosomal protein L27p
Mfumv2_1500	1095	660	522	522	701	511	511	1602	1739	1739	GTP-binding protein Obg
Mfumv2_1501	351	162	41	41	184	43	43	198	69	69	hypothetical protein
Mfumv2_1503	1710	168	165	208	106	87	121	163	219	277	hypothetical protein
Mfumv2_1504	204	14	2	2	29	4	4	25	5	5	hypothetical protein
Mfumv2_1508	849	215	106	132	118	51	67	129	80	109	Transposase, IS605 OrfB
Mfumv2_1510	639	626	289	289	472	200	201	568	359	360	Ribonuclease HII (EC 3.1.26.4)
Mfumv2_1511	345	999	249	249	731	167	168	1219	415	417	LSU ribosomal protein L19p
Mfumv2_1512	708	727	372	372	668	315	315	1544	1084	1084	tRNA (Guanine37-N1) -methyltransferase (EC 2.1.1.31)
Mfumv2_1513	258	1851	343	345	1478	254	254	1935	495	495	SSU ribosomal protein S16p
Mfumv2_1514	1314	445	422	422	466	407	408	464	604	604	Signal recognition particle, subunit Ffh SRP54 (TC 3.A.5.1.1)
Mfumv2_1515	1392	587	589	590	737	683	683	981	1352	1354	hypothetical protein
Mfumv2_1516	768	680	377	377	1013	518	518	745	565	567	20S proteasome, alpha subunit
Mfumv2_1517	837	774	468	468	1306	727	728	1199	995	995	Proteasome subunit beta (EC 3.4.25.1), bacterial
Mfumv2_1518	186	4347	584	584	6046	749	749	6956	1279	1283	hypothetical protein
Mfumv2_1519	1548	1014	1133	1134	1451	1494	1496	894	1372	1372	Pup ligase PafA' paralog, possible component of postulated heterodimer PafA-PafA'
Mfumv2_1520	1647	653	776	777	1087	1191	1192	944	1539	1541	Bacterial proteasome-activating AAA-ATPase (PAN)
Mfumv2_1521	735	471	250	250	566	277	277	251	183	183	Orotidine 5'-phosphate decarboxylase (EC 4.1.1.23)
Mfumv2_1522	861	127	79	79	113	65	65	221	189	189	4-diphosphocytidyl-2-C-methyl-D-erythritol kinase (EC 2.7.1.148)
Mfumv2_1523	1185	259	221	222	323	255	255	292	343	343	Ferredoxin reductase
Mfumv2_1524	690	626	311	312	659	303	303	512	350	350	Queuosine Biosynthesis QueE Radical SAM
Mfumv2_1525	747	1060	572	572	939	467	467	828	613	613	hypothetical protein-signal peptide and transmembrane prediction
Mfumv2_1526	696	149	75	75	235	109	109	245	169	169	PDZ domain containing protein
Mfumv2_1527	300	277	60	60	596	119	119	316	94	94	hypothetical protein
Mfumv2_1528	933	902	602	608	698	433	434	444	409	411	Oxidoreductase
Mfumv2_1529	399	62	18	18	38	10	10	136	54	54	Queuosine biosynthesis QueD, PTPS-I
Mfumv2_1530	1461	116	122	122	138	133	134	257	371	372	Lysyl-tRNA synthetase (class II) (EC 6.1.1.6)
Mfumv2_1531	576	62	26	26	109	42	42	249	142	142	hypothetical protein
Mfumv2_1532	399	38	11	11	64	17	17	45	18	18	putative exported protein
Mfumv2_1533	135	0	0	0	11	1	1	0	0	0	hypothetical protein
Mfumv2_1535	129	54	5	5	93	8	8	78	9	10	hypothetical protein
Mfumv2_1536	117	36	3	3	26	2	2	9	1	1	hypothetical protein
Mfumv2_1537	894	34	22	22	49	29	29	77	68	68	Methyltransferase (EC 2.1.1.-)
Mfumv2_1538	117	189	16	16	359	28	28	121	14	14	hypothetical protein
Mfumv2_1539	684	269	133	133	187	85	85	35	24	24	hypothetical protein
Mfumv2_1540	171	16	2	2	9	1	1	24	4	4	hypothetical protein
Mfumv2_1543	177	8	1	1	17	2	2	23	4	4	hypothetical protein
Mfumv2_1544	123	34	3	3	12	1	1	49	6	6	hypothetical protein
Mfumv2_1546	591	190	81	81	282	111	111	191	112	112	Error-prone repair protein UmuD
Mfumv2_1547	1266	124	113	113	244	206	206	209	263	263	Error-prone, lesion bypass DNA polymerase V (UmuC)
Mfumv2_1548	138	40	4	4	185	17	17	95	13	13	hypothetical protein
Mfumv2_1549	186	223	30	30	202	25	25	211	39	39	hypothetical protein
Mfumv2_1551	1257	58	53	53	76	64	64	116	143	144	glycosyl transferase group 1
Mfumv2_1552	162	9	1	1	19	2	2	44	7	7	hypothetical protein
Mfumv2_1553	456	24	8	8	16	5	5	42	19	19	Starvation lipoprotein Slp paralog
Mfumv2_1554	117	24	2	2	26	2	2	0	0	0	hypothetical protein
Mfumv2_1555	123	11	1	1	12	1	1	57	7	7	hypothetical protein
Mfumv2_1556	795	155	89	89	293	155	155	197	155	155	hypothetical protein
Mfumv2_1557	1116	29	23	23	34	25	25	114	126	126	5-methylthioribose kinase

Mfumv2_1558	483	181	63	63	183	59	59	194	93	93	Phosphohistidine Phosphatase, SixA
Mfumv2_1559	288	38	8	8	52	10	10	25	7	7	hypothetical protein
Mfumv2_1560	1890	107	146	146	37	46	46	54	101	101	Cadmium-transporting ATPase (EC 3.6.3.3)
Mfumv2_1561	120	35	3	3	38	3	3	25	3	3	hypothetical protein
Mfumv2_1562	828	247	223	223	608	321	320	27	75	77	hypothetical protein
Mfumv2_1563	714	266	227	227	818	289	289	36	19	19	hypothetical protein
Mfumv2_1564	696	751	232	232	1596	379	379	239	25	25	Ni,Fe-hydrogenase I cytochrome b subunit
Mfumv2_1565	1800	1370	2081	2083	3219	1913	1913	47	417	426	Uptake hydrogenase large subunit (EC 1.12.99.6)
Mfumv2_1566	1119	1730	1393	1398	2456	2399	2399	60	52	52	Uptake hydrogenase small subunit precursor (EC 1.12.99.6)
Mfumv2_1567	141	0	0	0	0	0	0	0	0	0	hypothetical protein
Mfumv2_1569	1197	246	213	213	218	174	174	1509	1791	1791	NAD-dependent formate dehydrogenase (EC 1.2.1.2)
Mfumv2_1570	693	26	13	13	19	9	9	45	31	31	FIG056164: rhomboid family serine protease
Mfumv2_1571	1257	356	323	323	440	368	368	170	212	212	Uncharacterized conserved protein
Mfumv2_1572	123	34	3	3	49	4	4	74	9	9	hypothetical protein
Mfumv2_1573	354	74	19	19	157	36	37	239	83	84	hypothetical protein
Mfumv2_1574	414	43	13	13	69	19	19	88	36	36	Glyoxalase family protein
Mfumv2_1575	957	246	167	170	344	219	219	435	413	413	Alpha amylase, catalytic region
Mfumv2_1576	1146	4221	3409	3494	358	268	273	147	166	167	hypothetical protein
Mfumv2_1577	1158	368	308	308	497	383	383	108	124	124	Glycosyltransferase (EC 2.4.1.-)
Mfumv2_1578	1119	157	127	127	176	131	131	184	204	204	Serine--pyruvate aminotransferase (EC 2.6.1.51) / L-alanine:glyoxylate aminotransferase (EC 2.6.1.44)
Mfumv2_1579	378	286	78	78	143	36	36	181	68	68	Arsenate reductase (EC 1.20.4.1)
Mfumv2_1580	825	176	105	105	213	117	117	345	282	282	Conserved domain protein
Mfumv2_1581	858	852	528	528	903	516	516	718	611	611	Uncharacterized protein conserved in bacteria, NMA0228-like
Mfumv2_1582	234	3775	637	638	3169	491	494	974	226	226	hypothetical protein
Mfumv2_1585	2142	151	233	233	307	438	438	287	609	610	Glycogen debranching enzyme (EC 3.2.1.-)
Mfumv2_1586	1845	107	143	143	138	167	170	183	334	334	Glucoamylase (EC 3.2.1.3)
Mfumv2_1591	114	24	2	2	40	3	3	35	4	4	hypothetical protein
Mfumv2_1592	210	119	18	18	100	14	14	53	11	11	hypothetical protein
Mfumv2_1596	2427	152	266	266	16	26	26	35	85	85	Outer membrane receptor protein, mostly Fe transport
Mfumv2_1600	612	262	110	116	172	62	70	178	105	108	Multimeric flavodoxin WrbA
Mfumv2_1601	750	18	10	10	40	20	20	35	26	26	Opacity protein or related surface antigen
Mfumv2_1602	2454	13	23	23	48	78	79	24	59	59	Phosphoenolpyruvate synthase (EC 2.7.9.2)
Mfumv2_1603	630	7	3	3	7	3	3	18	11	11	hypothetical protein
Mfumv2_1604	1116	12	10	10	42	30	31	33	35	36	Particulate methane monooxygenase B-subunit (EC 1.14.13.25)
Mfumv2_1605	750	18	10	10	138	69	69	66	49	49	Particulate methane monooxygenase A-subunit (EC 1.14.13.25)
Mfumv2_1606	864	59	37	37	566	326	326	105	90	90	Particulate methane monooxygenase C-subunit (EC 1.14.13.25)
Mfumv2_1608	1332	94	90	90	112	96	99	107	141	141	Glycolate dehydrogenase (EC 1.1.99.14), iron-sulfur subunit GlcF
Mfumv2_1609	1392	228	228	229	98	90	91	59	82	82	Fe-S protein, homolog of lactate dehydrogenase SO1521
Mfumv2_1610	1581	49	56	56	122	128	128	56	88	88	Glucose-methanol-choline (GMC) oxidoreductase:NAD binding site
Mfumv2_1611	576	41	17	17	94	36	36	44	25	25	hypothetical protein
Mfumv2_1612	390	0	0	0	162	42	42	41	16	16	Lactoylglutathione lyase (EC 4.4.1.5)
Mfumv2_1613	141	79	8	8	64	6	6	36	5	5	hypothetical protein
Mfumv2_1614	120	69	6	6	25	2	2	59	7	7	hypothetical protein
Mfumv2_1615	1977	206	293	294	436	573	574	353	691	692	transcriptional regulator
Mfumv2_1616	1065	820	629	631	1211	858	859	739	780	780	2-keto-3-deoxy-D-arabino-heptulosonate-7-phosphate synthase I alpha (EC 2.5.1.54)
Mfumv2_1617	1074	35	14	27	52	17	37	68	40	72	A/G-specific adenine glycosylase (EC 3.2.2.-)
Mfumv2_1618	702	201	26	102	261	26	122	56	20	39	Ribonuclease HIII (EC 3.1.26.4)
Mfumv2_1619	141	206	21	21	106	10	10	64	9	9	hypothetical protein
Mfumv2_1620	144	462	48	48	167	16	16	105	15	15	hypothetical protein
Mfumv2_1621	744	229	123	123	75	37	37	313	231	231	transcriptional regulator, Crp/Fnr family

Mfumv2_1623	147	791	84	84	1440	141	141	55	8	8	hypothetical protein
Mfumv2_1624	483	1347	470	470	2229	716	717	29	14	14	cytochrome c oxidase subunit II
Mfumv2_1625	1686	1058	1284	1288	1475	1649	1656	13077	21758	21864	Cytochrome c oxidase (B(O/a)3-type) chain I (EC 1.9.3.1)
Mfumv2_1626	156	142	16	16	0	0	0	13	2	2	hypothetical protein
Mfumv2_1627	312	44	10	10	34	7	7	29	9	9	hypothetical protein
Mfumv2_1628	132	0	0	0	68	6	6	46	6	6	hypothetical protein
Mfumv2_1629	882	50	15	32	19	6	11	40	19	35	A/G-specific adenine glycosylase (EC 3.2.2.-)
Mfumv2_1633	1593	213	245	245	251	266	266	196	309	309	HtrA protease/chaperone protein
Mfumv2_1634	903	210	137	137	234	141	141	156	140	140	Rhomboid family protein
Mfumv2_1635	1104	508	404	405	666	489	490	449	492	492	Fructose-1,6-bisphosphatase, GlpX type (EC 3.1.3.11)
Mfumv2_1636	618	119	53	53	63	26	26	73	45	45	Nicotinate-nucleotide adenylyltransferase (EC 2.7.7.18)
Mfumv2_1637	135	103	10	10	67	6	6	45	5	6	hypothetical protein
Mfumv2_1638	438	104	33	33	117	34	34	99	43	43	Iojap protein
Mfumv2_1639	1023	60	44	44	63	43	43	123	125	125	Potassium channel protein
Mfumv2_1640	939	66	45	45	75	47	47	368	343	343	dTDP-glucose 4,6-dehydratase (EC 4.2.1.46)
Mfumv2_1641	1062	134	103	103	204	144	144	418	440	440	TsaD/Kae1/Qri7 protein, required for threonylcarbamoyladenosine t(6)A37 formation in tRNA
Mfumv2_1642	1170	338	286	286	420	327	327	893	1035	1036	Nucleoside diphosphate kinase (EC 2.7.4.6)
Mfumv2_1643	1164	344	289	289	509	395	395	800	923	923	Aspartate aminotransferase (EC 2.6.1.1)
Mfumv2_1644	333	333	78	80	451	99	100	715	231	236	Predicted membrane protein
Mfumv2_1645	1173	61	52	52	76	59	59	150	174	174	hypothetical protein
Mfumv2_1646	924	390	260	260	354	218	218	242	222	222	Malate dehydrogenase (EC 1.1.1.37)
Mfumv2_1648	198	14	1	2	0	0	0	25	4	5	hypothetical protein
Mfumv2_1649	198	203	29	29	182	23	24	316	62	62	hypothetical protein
Mfumv2_1651	354	47	12	12	51	12	12	100	35	35	hypothetical protein
Mfumv2_1652	1323	45	43	43	44	39	39	82	107	108	alternate gene name: yzbB
Mfumv2_1653	642	183	85	85	138	58	59	237	151	151	peptidase, M50 family
Mfumv2_1654	1035	218	163	163	290	200	200	467	479	479	protein of unknown function DUF59
Mfumv2_1655	240	288	50	50	525	84	84	391	93	93	hypothetical protein
Mfumv2_1656	138	50	5	5	87	8	8	460	63	63	hypothetical protein
Mfumv2_1657	1425	138	142	142	117	111	111	457	646	646	hypothetical protein
Mfumv2_1658	564	98	40	40	83	31	31	134	75	75	Probable transmembrane protein
Mfumv2_1659	225	597	97	97	540	81	81	247	55	55	hypothetical protein
Mfumv2_1660	792	182	104	104	243	126	128	252	198	198	ABC-type multidrug transport system, permease component
Mfumv2_1661	738	49	26	26	69	34	34	66	48	48	ABC-type multidrug transport system, ATPase component
Mfumv2_1662	438	25	8	8	27	8	8	28	12	12	D-tyrosyl-tRNA(Tyr) deacylase
Mfumv2_1663	1158	77	63	64	78	60	60	104	118	119	Na+/H+ antiporter
Mfumv2_1664	993	197	140	141	268	177	177	167	164	164	Putative dihydropyrimidine dehydrogenase [NADP+], similar to dihydroorotate dehydrogenase
Mfumv2_1665	162	85	10	10	83	9	9	87	14	14	hypothetical protein
Mfumv2_1666	606	78	34	34	79	32	32	115	68	69	hypothetical protein
Mfumv2_1667	141	354	36	36	224	21	21	114	16	16	hypothetical protein
Mfumv2_1671	150	74	8	8	70	7	7	121	18	18	hypothetical protein
Mfumv2_1674	234	77	13	13	45	5	7	43	9	10	hypothetical protein
Mfumv2_1675	195	57	8	8	46	6	6	57	11	11	hypothetical protein
Mfumv2_1676	3255	65	153	153	82	177	178	60	195	195	acriflavin resistance protein
Mfumv2_1677	3192	57	132	132	83	174	176	66	209	209	acriflavin resistance protein
Mfumv2_1678	507	371	136	136	412	139	139	249	125	125	hypothetical protein
Mfumv2_1679	597	1046	451	451	1084	431	431	635	376	376	RNA polymerase sigma factor RpoE
Mfumv2_1680	1539	73	80	81	71	72	73	107	164	164	Type I secretion outer membrane protein, TolC precursor
Mfumv2_1681	162	85	10	10	74	8	8	62	10	10	hypothetical protein
Mfumv2_1682	1083	345	270	270	377	272	272	348	374	374	metal dependent phosphohydrolase

Mfumv2_1683	897	219	142	142	209	125	125	188	167	167	Phosphoribosylaminoimidazole-succinocarboxamide synthase (EC 6.3.2.6)
Mfumv2_1684	2550	171	315	315	145	247	247	143	362	362	glycosyl transferase, family 2
Mfumv2_1685	1854	122	163	163	150	185	185	86	158	158	ABC transporter related
Mfumv2_1686	1146	251	208	208	316	241	241	194	220	220	transcriptional regulator of molybdate metabolism, XRE family
Mfumv2_1687	1113	183	147	147	277	204	205	233	257	257	Cell division protein FtsZ (EC 3.4.24.-)
Mfumv2_1688	1239	255	228	228	236	195	195	241	296	296	Cell division protein FtsA
Mfumv2_1689	870	204	128	128	261	151	151	180	154	155	Cell division protein FtsQ
Mfumv2_1690	930	216	144	145	360	223	223	347	320	320	D-alanine--D-alanine ligase (EC 6.3.2.4)
Mfumv2_1691	2286	234	386	387	311	474	474	340	768	771	UDP-N-acetylmuramate--alanine ligase (EC 6.3.2.8)
Mfumv2_1692	1110	230	184	184	327	239	242	302	331	332	UDP-N-acetylglucosamine--N-acetylmuramyl-(pentapeptide) pyrophosphoryl-undecaprenol N-acetylglucosamine transferase (EC 2.4.1.227)
Mfumv2_1693	1182	110	94	94	131	103	103	118	138	138	Cell division protein FtsW
Mfumv2_1694	687	119	59	59	166	76	76	201	137	137	hypothetical protein(EC:3.2.1.-)
Mfumv2_1695	1101	91	72	72	100	73	73	180	197	197	Phospho-N-acetylmuramoyl-pentapeptide-transferase (EC 2.7.8.13)
Mfumv2_1696	1380	204	203	203	208	191	191	236	320	323	UDP-N-acetylmuramoylalanyl-D-glutamyl-2,6-diaminopimelate--D-alanyl-D-alanine ligase (EC 6.3.2.10)
Mfumv2_1700	1488	151	161	162	171	169	169	191	281	282	UDP-N-acetylmuramoylalanyl-D-glutamate--2,6-diaminopimelate ligase (EC 6.3.2.13)
Mfumv2_1702	177	0	0	0	51	6	6	40	7	7	hypothetical protein
Mfumv2_1704	222	499	80	80	480	71	71	432	95	95	hypothetical protein
Mfumv2_1705	120	185	16	16	200	16	16	395	47	47	hypothetical protein
Mfumv2_1706	945	397	271	271	559	352	352	395	370	370	rRNA small subunit methyltransferase H
Mfumv2_1707	441	1196	381	381	1038	304	305	759	332	332	Cell division protein MraZ
Mfumv2_1708	120	288	25	25	238	19	19	361	43	43	hypothetical protein
Mfumv2_1709	690	163	81	81	191	88	88	145	99	99	Glucosamine-1-phosphate N-acetyltransferase (EC 2.3.1.157)
Mfumv2_1710	1005	81	59	59	100	66	67	74	74	74	BatA (Bacteroides aerotolerance operon)
Mfumv2_1711	918	63	42	42	93	57	57	104	95	95	hypothetical protein PA3071
Mfumv2_1712	981	155	110	110	148	97	97	241	233	234	MoxR-like ATPase in aerotolerance operon
Mfumv2_1713	159	9	1	1	38	4	4	63	10	10	hypothetical protein
Mfumv2_1714	1050	467	354	354	505	351	353	139	145	145	Heme A synthase, cytochrome oxidase biogenesis protein Cox15-CtaA
Mfumv2_1715	951	370	252	254	508	322	322	126	119	119	Heme O synthase, protoheme IX farnesyltransferase (EC 2.5.1.-) COX10-CtaB
Mfumv2_1716	1434	136	141	141	140	134	134	153	217	217	TPR repeats containing protein
Mfumv2_1717	1005	61	44	44	61	39	41	159	158	158	hypothetical protein
Mfumv2_1718	543	520	204	204	589	212	213	574	308	309	isochorismatase hydrolase
Mfumv2_1719	1833	390	516	516	580	706	708	562	1021	1021	GTP-binding protein TypA/BipA
Mfumv2_1720	927	87	58	58	144	89	89	205	188	188	hypothetical protein
Mfumv2_1721	1095	104	82	82	62	45	45	45	49	49	hypothetical protein
Mfumv2_1723	189	116912	15840	15959	23314	2910	2935	59	11	11	hypothetical protein
Mfumv2_1726	129	75	7	7	23	2	2	31	4	4	hypothetical protein
Mfumv2_1727	1299	71	67	67	76	66	66	90	116	116	DNA recombination protein RmuC
Mfumv2_1728	2154	105	162	163	210	301	301	199	423	425	Polyphosphate kinase (EC 2.7.4.1)
Mfumv2_1729	396	1273	364	364	1202	317	317	1064	418	418	LSU ribosomal protein L17p
Mfumv2_1730	987	1285	914	916	1278	840	840	2235	2187	2187	DNA-directed RNA polymerase alpha subunit (EC 2.7.7.6)
Mfumv2_1731	609	1189	522	523	1699	689	689	2161	1304	1305	SSU ribosomal protein S4p (S9e)
Mfumv2_1732	543	2348	920	921	3478	1258	1258	3256	1751	1753	SSU ribosomal protein S11p (S14e)
Mfumv2_1733	396	1399	400	400	2510	661	662	2386	937	937	SSU ribosomal protein S13p (S18e)
Mfumv2_1734	819	720	425	426	1402	763	765	2147	1737	1744	Methionine aminopeptidase (EC 3.4.11.18)
Mfumv2_1735	270	123	24	24	295	53	53	1143	306	306	SSU ribosomal protein S20p
Mfumv2_1736	1743	284	356	358	96	111	112	141	244	244	Arginyl-tRNA synthetase (EC 6.1.1.19)
Mfumv2_1737	1092	246	194	194	263	191	191	319	345	345	sensor protein fixL(EC:2.7.3.-)
Mfumv2_1738	714	124	64	64	206	98	98	284	201	201	hypothetical protein
Mfumv2_1739	1521	38	42	42	46	47	47	78	117	117	hypothetical protein

Mfumv2_1740	1248	242	218	218	191	159	159	232	287	287	protein of unknown function DUF763
Mfumv2_1741	1062	239	152	183	49	22	35	18	12	19	hypothetical protein
Mfumv2_1744	1356	204	200	200	47	41	42	36	48	48	protein of unknown function DUF1504
Mfumv2_1745	1395	56	56	56	140	129	130	143	198	198	Adenosylmethionine-8-amino-7-oxononanoate aminotransferase (EC 2.6.1.62)
Mfumv2_1746	1179	153	130	130	211	166	166	233	271	272	8-amino-7-oxononanoate synthase (EC 2.3.1.47)
Mfumv2_1747	1221	138	122	122	252	205	205	226	273	274	hypothetical protein
Mfumv2_1748	147	9	1	1	0	0	0	0	0	0	hypothetical protein
Mfumv2_1749	660	36	17	17	82	36	36	55	36	36	phosphoesterase PA-phosphatase related
Mfumv2_1750	2064	193	286	287	239	328	329	213	436	436	Lead, cadmium, zinc and mercury transporting ATPase (EC 3.6.3.3) (EC 3.6.3.5); Copper-translocating P-type ATPase (EC 3.6.3.4)
Mfumv2_1751	996	457	329	329	529	351	351	649	641	641	Aspartate-semialdehyde dehydrogenase (EC 1.2.1.11)
Mfumv2_1752	609	621	273	273	688	279	279	563	340	340	hypothetical protein
Mfumv2_1753	1773	132	169	169	139	163	164	275	484	484	hypothetical protein
Mfumv2_1754	363	324	85	85	414	100	100	344	124	124	hypothetical protein
Mfumv2_1755	699	252	127	127	479	223	223	247	171	171	hypothetical protein
Mfumv2_1756	468	355	120	120	796	248	248	278	129	129	Predicted metal transporter
Mfumv2_1757	399	809	233	233	1975	523	525	270	107	107	hypothetical protein
Mfumv2_1758	1656	803	958	960	1155	1272	1274	342	558	561	Multicopper oxidase
Mfumv2_1759	696	1194	600	600	1810	839	839	281	194	194	hypothetical protein
Mfumv2_1760	135	892	87	87	1101	98	99	247	33	33	hypothetical protein
Mfumv2_1761	837	175	106	106	208	116	116	217	180	180	hypothetical protein
Mfumv2_1762	1851	169	226	226	218	269	269	178	326	326	Lipid A export ATP-binding/permease protein MsbA (EC 3.6.3.25)
Mfumv2_1763	597	37	16	16	33	13	13	79	47	47	COG0596: Predicted hydrolases or acyltransferases (alpha/beta hydrolase superfamily)
Mfumv2_1764	807	151	88	88	221	118	119	284	227	227	hypothetical protein
Mfumv2_1765	1431	307	316	317	452	430	431	185	262	262	actin binding protein
Mfumv2_1767	2526	23	42	42	17	29	29	31	78	78	Outer membrane receptor for ferric coprogen and ferric-rhodotorulic acid
Mfumv2_1768	537	93	35	36	112	40	40	56	30	30	hypothetical protein
Mfumv2_1769	1146	242	198	200	265	202	202	397	451	451	NAD(P) transhydrogenase alpha subunit (EC 1.6.1.2)
Mfumv2_1770	183	242	32	32	107	13	13	287	52	52	NAD(P) transhydrogenase alpha subunit (EC 1.6.1.2)
Mfumv2_1771	1377	269	266	268	207	190	190	326	445	445	NAD(P) transhydrogenase subunit beta (EC 1.6.1.2)
Mfumv2_1772	633	147	67	67	126	53	53	473	297	297	Globin domain
Mfumv2_1773	759	212	113	116	152	77	77	276	208	208	hypothetical protein
Mfumv2_1774	1566	1472	1665	1665	1762	1838	1838	788	1223	1223	hypothetical protein
Mfumv2_1776	1563	16616	18675	18757	27811	28916	28953	6857	10591	10628	Outer membrane lipoprotein omp16 precursor
Mfumv2_1777	3039	220	481	483	372	753	753	342	1030	1031	hypothetical protein
Mfumv2_1778	147	47	5	5	61	6	6	69	10	10	hypothetical protein
Mfumv2_1779	765	92	51	51	110	56	56	149	113	113	hypothetical protein
Mfumv2_1780	738	86	46	46	71	35	35	105	77	77	TPR repeats containing protein
Mfumv2_1783	249	1718	289	309	1242	185	206	1126	197	278	hypothetical protein
Mfumv2_1785	669	122	59	59	130	58	58	327	217	217	6-phosphogluconolactonase (EC 3.1.1.31), eukaryotic type
Mfumv2_1786	1569	199	226	226	173	180	181	273	424	425	Glucose-6-phosphate 1-dehydrogenase (EC 1.1.1.49)
Mfumv2_1787	633	453	206	207	555	234	234	408	255	256	hypothetical protein
Mfumv2_1790	141	0	0	0	11	1	1	43	6	6	hypothetical protein
Mfumv2_1791	1290	755	703	703	826	710	710	15059	19258	19263	Particulate methane monooxygenase B-subunit (EC 1.14.13.25)
Mfumv2_1792	738	976	520	520	559	275	275	24921	18232	18238	Particulate methane monooxygenase A-subunit (EC 1.14.13.25)
Mfumv2_1794	1311	4351	4111	4120	10253	8951	8953	111	144	144	Particulate methane monooxygenase B-subunit (EC 1.14.13.25)
Mfumv2_1795	801	4225	2441	2444	12147	6476	6481	53	42	42	Particulate methane monooxygenase A-subunit (EC 1.14.13.25)
Mfumv2_1796	828	26675	15937	15952	31613	17429	17435	283	232	232	Particulate methane monooxygenase C-subunit (EC 1.14.13.25)
Mfumv2_1797	132	420	40	40	637	56	56	153	20	20	hypothetical protein
Mfumv2_1798	246	1069	188	190	1086	178	178	143	35	35	hypothetical protein

Mfumv2_1799	699	3294	1660	1663	6559	3051	3054	322	223	223	hypothetical protein
Mfumv2_1800	1110	317	252	254	289	213	214	52	57	57	ABC transport system, permease component YbhR
Mfumv2_1801	1125	43	35	35	71	53	53	39	43	43	ABC transport system, permease component YbhS
Mfumv2_1802	1752	51	64	64	58	68	68	66	114	114	ABC transporter multidrug efflux pump, fused ATP-binding domains
Mfumv2_1803	879	90	57	57	121	71	71	67	58	58	Predicted membrane fusion protein (MFP) component of efflux pump, membrane anchor protein YbhG
Mfumv2_1804	1335	76	73	73	65	58	58	43	57	57	Type I secretion outer membrane protein, TolC precursor
Mfumv2_1805	2079	138	206	207	175	243	243	462	938	953	NAD synthetase (EC 6.3.1.5) / Glutamine amidotransferase chain of NAD synthetase
Mfumv2_1806	153	489	54	54	206	21	21	119	18	18	hypothetical protein
Mfumv2_1807	2160	149	232	232	204	293	293	140	298	299	Cytochrome c-type biogenesis protein DsbD, protein-disulfide reductase (EC 1.8.1.8)
Mfumv2_1808	1449	112	117	117	170	163	164	83	119	119	Excinuclease ABC subunit C
Mfumv2_1811	2277	246	402	404	225	342	342	193	436	436	Phosphoribosylformylglycinamidine synthase, synthetase subunit (EC 6.3.5.3)
Mfumv2_1812	1161	295	247	247	323	250	250	125	143	144	N-acetylglucosamine-6-phosphate deacetylase (EC 3.5.1.25)
Mfumv2_1813	1395	177	177	178	265	246	246	231	319	320	tRNA-i(6)A37 methylthiotransferase
Mfumv2_1814	1206	238	207	207	178	143	143	271	324	324	putative membrane protein
Mfumv2_1815	3003	77	166	167	58	115	116	69	206	206	Proline dehydrogenase (EC 1.5.99.8) (Proline oxidase) / Delta-1-pyrroline-5-carboxylate dehydrogenase (EC 1.5.1.12)
Mfumv2_1816	1233	93	83	83	49	40	40	154	188	188	TPR repeats containing protein
Mfumv2_1817	714	347	179	179	393	186	187	661	468	468	3-oxoacyl-[acyl-carrier protein] reductase (EC 1.1.1.100)
Mfumv2_1818	594	846	363	363	1021	403	404	710	418	418	5-formyltetrahydrofolate cyclo-ligase (EC 6.3.3.2)
Mfumv2_1819	156	435	49	49	587	61	61	317	49	49	hypothetical protein
Mfumv2_1820	687	758	376	376	887	406	406	686	467	467	hypothetical protein
Mfumv2_1821	555	409	164	164	573	208	212	372	203	205	hypothetical protein
Mfumv2_1822	144	29	3	3	73	7	7	49	7	7	hypothetical protein
Mfumv2_1823	129	268	25	25	221	19	19	211	27	27	hypothetical protein
Mfumv2_1824	168	16	2	2	9	1	1	54	9	9	hypothetical protein
Mfumv2_1825	186	119	15	16	73	9	9	87	16	16	hypothetical protein
Mfumv2_1826	5091	665	2444	2445	418	1416	1416	658	3319	3320	DNA helicase
Mfumv2_1827	168	799	97	97	733	82	82	816	136	136	hypothetical protein
Mfumv2_1830	213	689	103	106	317	43	45	374	77	79	hypothetical protein
Mfumv2_1831	1068	36	28	28	58	40	41	40	42	42	hypothetical protein
Mfumv2_1832	588	318	135	135	531	208	208	110	64	64	Globin domain
Mfumv2_1833	501	166	60	60	156	52	52	185	92	92	2-C-methyl-D-erythritol 2,4-cyclodiphosphate synthase (EC 4.6.1.12)
Mfumv2_1834	1155	158	131	132	165	127	127	148	168	169	2-hydroxy-3-oxopropionate reductase (EC 1.1.1.60)
Mfumv2_1835	1725	71	87	88	90	103	103	102	174	174	hypothetical protein
Mfumv2_1836	2715	91	178	178	149	269	269	127	342	342	[Protein-PII] uridylyltransferase (EC 2.7.7.59)
Mfumv2_1837	744	195	105	105	192	94	95	207	153	153	Pyridoxine 5'-phosphate synthase (EC 2.6.99.2)
Mfumv2_1838	372	1012	272	272	1174	291	291	623	230	230	Holo-[acyl-carrier protein] synthase (EC 2.7.8.7)
Mfumv2_1839	294	5199	1104	1104	6976	1366	1366	1039	303	303	hypothetical protein
Mfumv2_1840	114	12	1	1	13	1	1	88	10	10	hypothetical protein
Mfumv2_1841	930	168	113	113	210	130	130	413	381	381	Transcriptional regulator, AraC family
Mfumv2_1842	723	329	172	172	260	125	125	173	124	124	DNA-binding response regulator KdpE
Mfumv2_1843	2712	495	965	969	445	803	804	206	553	553	Osmosensitive K+ channel histidine kinase KdpD (EC 2.7.3.-)
Mfumv2_1844	663	950	453	455	969	428	428	1576	1036	1036	LSU ribosomal protein L25p
Mfumv2_1845	555	247	99	99	376	139	139	858	471	472	Peptidyl-tRNA hydrolase (EC 3.1.1.29)
Mfumv2_1846	336	692	168	168	858	192	192	930	310	310	SSU ribosomal protein S6p
Mfumv2_1847	441	2091	666	666	1971	579	579	2611	1142	1142	Single-stranded DNA-binding protein
Mfumv2_1848	579	902	377	377	801	305	309	1416	813	813	LSU ribosomal protein L9p
Mfumv2_1849	2703	76	147	148	80	144	144	187	501	502	Outer membrane protein assembly factor YaeT precursor
Mfumv2_1850	600	1357	587	588	963	385	385	866	515	515	Outer membrane protein H precursor
Mfumv2_1851	1062	703	536	539	739	523	523	737	774	776	UDP-3-O-[3-hydroxymyristoyl] glucosamine N-acyltransferase (EC 2.3.1.-)

Mfumv2_1852	1317	308	293	293	294	258	258	455	593	594	Homoserine dehydrogenase (EC 1.1.1.3)
Mfumv2_1853	1101	249	197	198	356	259	261	501	546	547	Threonine synthase (EC 4.2.3.1)
Mfumv2_1854	1368	503	496	497	672	612	612	1283	1739	1741	3-isopropylmalate dehydratase large subunit (EC 4.2.1.33)
Mfumv2_1855	342	421	104	104	496	113	113	631	214	214	hypothetical protein
Mfumv2_1856	2343	53	89	89	56	88	88	72	167	167	hypothetical protein
Mfumv2_1857	363	275	72	72	190	45	46	289	104	104	hypothetical protein
Mfumv2_1858	174	16	2	2	26	3	3	46	8	8	hypothetical protein
Mfumv2_1859	204	27	4	4	44	6	6	59	12	12	hypothetical protein
Mfumv2_1860	150	28	3	3	0	0	0	13	2	2	hypothetical protein
Mfumv2_1861	132	10	1	1	11	1	1	8	1	1	hypothetical protein
Mfumv2_1866	702	45	23	23	75	35	35	52	36	36	hypothetical protein
Mfumv2_1877	1236	20	18	18	47	39	39	77	94	94	hypothetical protein
Mfumv2_1878	681	43	21	21	29	13	13	25	17	17	hypothetical protein
Mfumv2_1879	714	91	47	47	86	41	41	145	103	103	hypothetical protein
Mfumv2_1880	120	46	4	4	50	4	4	84	10	10	hypothetical protein
Mfumv2_1881	840	196	119	119	202	113	113	212	177	177	D-alanyl-D-alanine carboxypeptidase
Mfumv2_1882	510	16	6	6	53	18	18	53	27	27	protein of unknown function DUF123
Mfumv2_1883	561	52	21	21	118	44	44	167	91	93	hypothetical protein
Mfumv2_1884	117	12	1	1	77	6	6	155	18	18	hypothetical protein
Mfumv2_1885	1458	111	117	117	98	94	95	180	260	260	Glycosyltransferase
Mfumv2_1886	645	82	38	38	95	41	41	163	104	104	hypothetical protein
Mfumv2_1887	846	205	125	125	273	154	154	249	209	209	Lipoate synthase
Mfumv2_1888	138	50	0	5	44	0	4	88	0	12	hypothetical protein
Mfumv2_1890	141	20	0	2	11	0	1	64	0	9	hypothetical protein
Mfumv2_1892	153	27	0	3	10	0	1	20	0	3	hypothetical protein
Mfumv2_1893	129	2673	241	249	1455	114	125	3557	438	455	hypothetical protein
Mfumv2_1894	768	310	170	172	272	139	139	293	222	223	N-acetylmuramoyl-L-alanine amidase (EC 3.5.1.28)
Mfumv2_1895	1083	60	47	47	93	67	67	83	89	89	beta-lactamase, putative
Mfumv2_1896	1089	52	40	41	36	26	26	49	53	53	conserved hypothetical protein
Mfumv2_1904	1242	152	135	136	164	136	136	66	80	81	amino acid permease family protein
Mfumv2_1905	1326	96	91	92	108	95	95	103	136	136	Adenosylmethionine-8-amino-7-oxononanoate aminotransferase (EC 2.6.1.62)
Mfumv2_1906	711	409	210	210	412	195	195	233	164	164	Hydrolases of the alpha/beta superfamily
Mfumv2_1907	1221	418	369	369	756	615	615	474	574	574	Argininosuccinate synthase (EC 6.3.4.5)
Mfumv2_1908	1974	800	1141	1141	1107	1455	1455	947	1854	1854	1-deoxy-D-xylulose 5-phosphate synthase (EC 2.2.1.7)
Mfumv2_1909	246	805	143	143	1251	205	205	828	202	202	Exodeoxyribonuclease VII small subunit (EC 3.1.11.6)
Mfumv2_1910	516	566	210	211	719	246	247	537	275	275	hypothetical protein
Mfumv2_1911	183	8	1	1	8	1	1	6	1	1	hypothetical protein
Mfumv2_1912	2646	138	263	263	242	425	427	321	842	843	Valyl-tRNA synthetase (EC 6.1.1.9)
Mfumv2_1913	1041	642	483	483	650	451	451	403	416	416	RecA protein
Mfumv2_1914	1878	828	1120	1123	1363	1701	1705	1665	3099	3101	RNA polymerase sigma factor RpoD
Mfumv2_1915	1776	90	115	115	92	109	109	149	263	263	DNA primase (EC 2.7.7.-)
Mfumv2_1916	1122	89	72	72	102	76	76	83	92	92	hypothetical protein
Mfumv2_1917	732	161	85	85	152	74	74	376	272	273	Nucleoside triphosphate pyrophosphohydrolase MazG (EC 3.6.1.8)
Mfumv2_1918	180	54	7	7	242	29	29	241	40	43	hypothetical protein
Mfumv2_1919	831	25	15	15	22	12	12	51	41	42	hypothetical protein
Mfumv2_1920	723	82	43	43	66	32	32	128	92	92	1-acyl-sn-glycerol-3-phosphate acyltransferase (EC 2.3.1.51)
Mfumv2_1921	663	84	40	40	154	68	68	179	118	118	Leucyl/phenylalanyl-tRNA--protein transferase (EC 2.3.2.6)
Mfumv2_1922	1239	171	153	153	237	196	196	269	330	330	Molybdopterin binding motif, CinA N-terminal domain / C-terminal domain of CinA type S
Mfumv2_1923	3021	504	1099	1100	736	1479	1482	569	1706	1706	Protein export cytoplasm protein SecA ATPase RNA helicase (TC 3.A.5.1.1)
Mfumv2_1924	1191	191	164	164	228	181	181	256	301	302	putative serine protease containing two PDZ domains(EC:3.4.21.-)

Mfumv2_1925	1083	137	107	107	172	124	124	264	283	283	Anthranilate phosphoribosyltransferase (EC 2.4.2.18)
Mfumv2_1926	1212	175	152	153	384	310	310	391	470	470	Carbamoyl-phosphate synthase small chain (EC 6.3.5.5)
Mfumv2_1927	1320	136	130	130	181	159	159	304	398	398	Dihydroorotase (EC 3.5.2.3)
Mfumv2_1928	981	85	60	60	57	37	37	197	191	192	Aspartate carbamoyltransferase (EC 2.1.3.2)
Mfumv2_1929	549	421	167	167	388	142	142	593	323	323	3-isopropylmalate dehydratase small subunit (EC 4.2.1.33)
Mfumv2_1930	1113	415	332	334	444	329	329	796	879	879	3-isopropylmalate dehydrogenase (EC 1.1.1.85)
Mfumv2_1931	1140	707	578	582	666	504	506	907	1022	1025	Heme biosynthesis protein related to NirD and NirG / Heme biosynthesis protein related to NirL and NirH
Mfumv2_1932	1848	85	113	113	115	141	141	133	243	243	Sulfate permease
Mfumv2_1933	1158	398	333	333	417	321	322	475	546	546	Tyrosyl-tRNA synthetase (EC 6.1.1.1)
Mfumv2_1934	435	1735	544	545	2054	595	595	1586	684	684	putative RNA-binding protein
Mfumv2_1935	387	186	52	52	209	54	54	73	28	28	ApaG protein
Mfumv2_1936	774	349	194	195	415	213	214	328	252	252	Phosphomethylpyrimidine kinase (EC 2.7.4.7)
Mfumv2_1937	1029	240	176	178	212	145	145	263	268	268	Alcohol dehydrogenase (EC 1.1.1.1)
Mfumv2_1938	534	137	53	53	143	51	51	117	62	62	hypothetical protein
Mfumv2_1939	1086	68	53	53	80	58	58	108	116	116	hypothetical protein
Mfumv2_1940	1194	48	41	41	41	33	33	163	192	193	hypothetical protein
Mfumv2_1941	1596	107	122	123	131	139	139	167	265	265	Glycogen branching enzyme, GH-57-type, archaeal (EC 2.4.1.18)
Mfumv2_1942	159	44	5	5	85	9	9	25	4	4	hypothetical protein
Mfumv2_1943	864	35	22	22	40	23	23	27	23	23	hypothetical protein
Mfumv2_1944	435	290	91	91	252	73	73	144	62	62	Arsenate reductase (EC 1.20.4.1)
Mfumv2_1945	348	231	58	58	349	81	81	125	43	43	Arsenical resistance operon repressor
Mfumv2_1946	1071	41	32	32	35	25	25	17	18	18	Arsenical-resistance protein ACR3
Mfumv2_1947	681	61	30	30	86	39	39	47	32	32	hypothetical protein
Mfumv2_1948	1020	91	67	67	72	49	49	54	55	55	FAD dependent oxidoreductase
Mfumv2_1949	1041	44	32	33	30	20	21	39	40	40	Chalcone and stilbene synthases domain protein
Mfumv2_1950	2412	48	84	84	50	81	81	67	161	161	hypothetical protein
Mfumv2_1951	114	0	0	0	0	0	0	27	3	3	hypothetical protein
Mfumv2_1952	492	8	3	3	3	1	1	14	7	7	Starvation lipoprotein Slp paralog
Mfumv2_1953	222	0	0	0	7	1	1	23	5	5	hypothetical protein
Mfumv2_1954	144	10	1	1	10	1	1	21	3	3	hypothetical protein
Mfumv2_1955	276	50	10	10	60	11	11	55	15	15	hypothetical protein
Mfumv2_1956	138	60	6	6	120	11	11	168	23	23	hypothetical protein
Mfumv2_1957	135	82	8	8	211	19	19	112	15	15	hypothetical protein
Mfumv2_1959	252	637	116	116	733	123	123	276	69	69	hypothetical protein
Mfumv2_1960	1407	105	107	107	46	42	43	42	58	58	outer membrane efflux protein
Mfumv2_1961	153	81	9	9	20	2	2	53	8	8	hypothetical protein
Mfumv2_1962	1326	28	27	27	34	30	30	34	45	45	FAD dependent oxidoreductase
Mfumv2_1963	276	216	41	43	370	64	68	183	48	50	hypothetical protein
Mfumv2_1964	168	190	23	23	107	12	12	150	25	25	hypothetical protein
Mfumv2_1965	1047	29	22	22	44	31	31	62	64	64	hypothetical protein
Mfumv2_1966	738	53	28	28	41	20	20	27	20	20	Periplasmic protein TonB
Mfumv2_1967	486	142	50	50	68	22	22	58	28	28	Cupin domain-containing protein
Mfumv2_1968	2970	143	306	307	191	377	378	129	379	379	Cell division protein FtsK
Mfumv2_1969	753	230	125	125	237	119	119	192	143	143	hypothetical protein
Mfumv2_1970	1440	103	107	107	109	105	105	190	272	272	Aspartyl-tRNA(Asn) amidotransferase subunit A (EC 6.3.5.6)
Mfumv2_1971	441	94	30	30	129	38	38	222	97	97	Cyanate hydratase (EC 4.2.1.104)
Mfumv2_1972	864	67	42	42	94	54	54	70	60	60	hypothetical protein
Mfumv2_1973	1365	264	260	260	205	186	186	172	233	233	Copper-containing nitrite reductase (EC 1.7.2.1)
Mfumv2_1974	1770	56	72	72	90	106	106	65	114	114	hypothetical protein
Mfumv2_1975	828	77	46	46	105	58	58	83	68	68	TPR repeat:HAT (Half-A-TPR) repeat

Mfumv2_1976	240	52	9	9	181	28	29	63	15	15	hypothetical protein
Mfumv2_1977	525	29	11	11	23	8	8	23	12	12	hypothetical protein
Mfumv2_1978	1290	194	181	181	173	147	149	231	295	295	Pyridine nucleotide-disulphide oxidoreductase associated with reductive pyrimidine catabolism
Mfumv2_1979	1341	71	69	69	122	109	109	188	250	250	Dihydropyrimidine dehydrogenase [NADP+] (EC 1.3.1.2)
Mfumv2_1980	1413	97	99	99	148	139	139	182	255	255	Dihydropyrimidinase (EC 3.5.2.2)
Mfumv2_1981	1245	93	84	84	122	101	101	159	196	196	N-carbamoyl-L-amino acid hydrolase (EC 3.5.1.87)
Mfumv2_1984	693	60	30	30	91	41	42	86	58	59	Periplasmic protein TonB
Mfumv2_1985	1884	41	56	56	37	46	46	47	88	88	Dipeptidyl anminopeptidase
Mfumv2_1986	960	130	90	90	133	85	85	62	59	59	putative capsule biosynthesis protein
Mfumv2_1987	747	63	34	34	44	22	22	271	201	201	Putative sulfate permease
Mfumv2_1988	876	419	265	265	626	365	365	953	828	828	Phosphoribulokinase (EC 2.7.1.19)
Mfumv2_1989	1050	665	504	504	540	378	378	815	849	849	Fructose-1,6-bisphosphatase, type I (EC 3.1.3.11)
Mfumv2_1990	1608	82	94	95	63	67	67	74	117	118	outer membrane efflux protein
Mfumv2_1991	3252	192	448	450	123	267	267	87	279	279	Acriflavin resistance protein
Mfumv2_1992	216	820	128	128	1425	205	205	565	121	121	hypothetical protein
Mfumv2_1993	1524	161	177	177	182	185	185	302	456	456	Prolyl-tRNA synthetase (EC 6.1.1.15)
Mfumv2_1994	321	142	33	33	224	48	48	361	114	115	Aspartyl-tRNA(Asn) amidotransferase subunit C (EC 6.3.5.6)
Mfumv2_1995	1452	228	239	239	272	263	263	532	766	766	Aspartyl-tRNA(Asn) amidotransferase subunit A (EC 6.3.5.6)
Mfumv2_1996	1458	292	308	308	487	473	473	638	923	923	Aspartyl-tRNA(Asn) amidotransferase subunit B (EC 6.3.5.6)
Mfumv2_1997	1788	645	832	833	575	684	685	356	631	632	N-acetylmuramic acid 6-phosphate etherase (EC 4.2.-.-)
Mfumv2_1998	2076	534	798	800	327	452	452	232	477	477	DNA ligase (EC 6.5.1.2)
Mfumv2_1999	672	109	52	53	308	138	138	212	140	141	hypothetical protein
Mfumv2_2000	1509	77	84	84	67	67	67	53	78	79	Glycerol kinase (EC 2.7.1.30)
Mfumv2_2003	765	177	98	98	183	93	93	283	215	215	Pantothenate kinase type III, CoaX-like (EC 2.7.1.33)
Mfumv2_2004	1548	665	743	744	881	906	908	789	1209	1211	HtrA protease/chaperone protein
Mfumv2_2005	468	1879	635	635	1883	587	587	687	319	319	Hypothetical protein DUF901, (C-terminal domain of ribosome protection-type Tc-resistance proteins)
Mfumv2_2006	969	307	215	215	397	256	256	583	560	560	Tryptophanyl-tRNA synthetase (EC 6.1.1.2)
Mfumv2_2007	816	188	111	111	230	125	125	305	247	247	Chorismate mutase I (EC 5.4.99.5)
Mfumv2_2008	1566	96	109	109	167	174	174	158	246	246	hypothetical protein
Mfumv2_2009	741	95	51	51	63	31	31	95	70	70	3-dehydroquinate dehydratase I (EC 4.2.1.10)
Mfumv2_2010	1137	1059	870	870	1136	859	860	937	1056	1057	Rod shape-determining protein MreB
Mfumv2_2011	933	217	146	146	383	238	238	376	348	348	Rod shape-determining protein MreC
Mfumv2_2012	135	51	5	5	145	13	13	97	13	13	hypothetical protein
Mfumv2_2013	486	68	24	24	154	50	50	102	49	49	Rod shape-determining protein MreD
Mfumv2_2014	2028	354	519	519	399	539	539	287	578	578	Cell division protein FtsI [Peptidoglycan synthetase] (EC 2.4.1.129)
Mfumv2_2015	1182	104	89	89	113	89	89	90	105	106	Rod shape-determining protein RodA
Mfumv2_2016	1572	156	176	177	247	259	259	179	279	279	Cytoplasmic axial filament protein CafA and Ribonuclease G (EC 3.1.4.-)
Mfumv2_2017	1266	367	334	336	439	369	370	423	522	531	Seryl-tRNA synthetase (EC 6.1.1.11)
Mfumv2_2018	2178	431	672	678	607	880	881	497	1068	1073	Tail-specific protease precursor (EC 3.4.21.102)
Mfumv2_2019	276	1480	294	295	1414	259	260	775	212	212	Translation initiation factor 1
Mfumv2_2020	891	154	99	99	138	82	82	173	153	153	hypothetical protein
Mfumv2_2021	147	179	19	19	266	26	26	137	20	20	hypothetical protein
Mfumv2_2022	1302	150	139	141	190	165	165	186	240	240	Phosphoribosylamine--glycine ligase (EC 6.3.4.13)
Mfumv2_2023	906	805	527	527	630	379	380	538	483	483	heat shock protein HtpX
Mfumv2_2024	1446	149	156	156	229	221	221	243	349	349	Succinate-semialdehyde dehydrogenase [NADP+] (EC 1.2.1.16)
Mfumv2_2025	819	272	161	161	262	143	143	215	174	175	hypothetical protein
Mfumv2_2026	132	472	45	45	637	56	56	428	56	56	hypothetical protein
Mfumv2_2027	123	16739	1485	1487	1477	121	121	1131	138	138	hypothetical protein
Mfumv2_2028	426	169	52	52	204	58	58	154	65	65	conserved hypothetical protein
Mfumv2_2029	678	227	111	111	350	158	158	229	154	154	Cytochrome oxidase biogenesis protein Sco1/SenC/PrrC, putative copper metallochaperone

Mfumv2_2030	795	273	157	157	198	105	105	226	178	178	ABC transporter, permease protein
Mfumv2_2031	1035	266	199	199	337	232	232	407	418	418	ABC transporter related
Mfumv2_2032	1374	211	209	209	276	251	253	254	346	346	3-deoxy-D-manno-octulosonic-acid transferase (EC 2.-.-.-)
Mfumv2_2033	948	212	144	145	241	152	152	124	117	117	tyrosine recombinase XerD
Mfumv2_2034	696	80	40	40	50	23	23	85	59	59	Transposase OrfB
Mfumv2_2035	2781	137	276	276	78	143	144	165	454	454	Phosphoenolpyruvate carboxylase (EC 4.1.1.31)
Mfumv2_2036	213	293	45	45	381	54	54	383	81	81	hypothetical protein
Mfumv2_2037	1515	507	552	555	879	885	887	1158	1740	1740	2-isopropylmalate synthase (EC 2.3.3.13)
Mfumv2_2038	1029	488	362	363	690	473	473	1211	1236	1236	Ketol-acid reductoisomerase (EC 1.1.1.86)
Mfumv2_2039	474	315	108	108	545	172	172	340	160	160	Acetolactate synthase small subunit (EC 2.2.1.6)
Mfumv2_2040	276	50	10	10	76	14	14	80	22	22	Acylphosphate phosphohydrolase (EC 3.6.1.7), putative
Mfumv2_2041	177	16	2	2	0	0	0	6	1	1	hypothetical protein
Mfumv2_2042	1125	187	152	152	188	141	141	149	166	166	Beta-hexosaminidase (EC 3.2.1.52)
Mfumv2_2043	2169	135	212	212	183	263	264	327	702	703	Long-chain-fatty-acid--CoA ligase (EC 6.2.1.3)
Mfumv2_2044	555	110	44	44	105	39	39	118	65	65	DNA recombination and repair protein RecO
Mfumv2_2045	333	62	15	15	117	26	26	88	29	29	Metal-dependent hydrolase YbeY, involved in rRNA and/or ribosome maturation and assembly
Mfumv2_2046	1620	146	170	171	296	317	319	181	290	290	Membrane protein containing HD superfamily hydrolase domain, YQFF ortholog
Mfumv2_2047	972	315	220	221	454	293	294	481	462	464	Phosphate starvation-inducible protein PhoH, predicted ATPase
Mfumv2_2048	552	40	16	16	5	2	2	53	28	29	NADPH-dependent FMN reductase
Mfumv2_2049	1173	102	86	86	84	66	66	83	96	96	Alanine dehydrogenase (EC 1.4.1.1)
Mfumv2_2050	675	64	31	31	71	32	32	143	95	96	Lipoate-protein ligase A
Mfumv2_2051	447	384	122	124	353	105	105	277	123	123	FKBP-type peptidyl-prolyl cis-trans isomerase
Mfumv2_2052	885	455	291	291	405	239	239	523	458	459	Octaprenyl diphosphate synthase (EC 2.5.1.90) / Dimethylallyltransferase (EC 2.5.1.1) / (2E,6E)-farnesyl diphosphate synthase (EC 2.5.1.10) / Geranylgeranyl diphosphate synthase (EC 2.5.1.29)
Mfumv2_2053	336	902	219	219	728	163	163	1177	392	392	hypothetical protein
Mfumv2_2057	264	142	27	27	17	3	3	31	8	8	hypothetical protein
Mfumv2_2058	123	56	5	5	85	7	7	82	10	10	hypothetical protein
Mfumv2_2059	135	21	2	2	0	0	0	7	1	1	hypothetical protein
Mfumv2_2060	165	50	6	6	18	2	2	18	3	3	hypothetical protein
Mfumv2_2061	1134	53	43	43	74	56	56	74	83	83	Phosphate ABC transporter, periplasmic phosphate-binding protein PstS (TC 3.A.1.7.1)
Mfumv2_2062	1026	94	70	70	107	72	73	166	169	169	Phosphate transport system permease protein PstC (TC 3.A.1.7.1)
Mfumv2_2063	840	94	57	57	46	26	26	62	52	52	Phosphate transport system permease protein PstA (TC 3.A.1.7.1)
Mfumv2_2064	822	96	57	57	139	76	76	217	177	177	Phosphate transport ATP-binding protein PstB (TC 3.A.1.7.1)
Mfumv2_2065	1155	85	71	71	116	89	89	85	97	97	Sodium-dependent transporter
Mfumv2_2066	762	122	67	67	341	173	173	269	202	203	Succinate dehydrogenase iron-sulfur protein (EC 1.3.99.1)
Mfumv2_2067	1956	200	281	283	367	476	478	390	755	756	Succinate dehydrogenase flavoprotein subunit (EC 1.3.99.1)
Mfumv2_2068	594	154	66	66	114	45	45	144	84	85	Succinate dehydrogenase cytochrome b subunit
Mfumv2_2069	729	198	101	104	224	107	109	129	93	93	haloacid dehalogenase, IA family protein
Mfumv2_2070	1326	133	127	127	188	166	166	151	198	198	Dihydrofolate synthase (EC 6.3.2.12) / Folylpolyglutamate synthase (EC 6.3.2.17)
Mfumv2_2071	1356	97	94	95	103	93	93	167	224	224	hypothetical protein
Mfumv2_2072	159	26	3	3	28	3	3	82	13	13	hypothetical protein
Mfumv2_2073	417	76	23	23	58	16	16	128	53	53	Protein of unknown function DUF374
Mfumv2_2074	135	31	3	3	22	2	2	37	5	5	hypothetical protein
Mfumv2_2075	375	96	26	26	64	16	16	83	31	31	CRISPR-associated protein, Csx3 family
Mfumv2_2076	270	72	14	14	206	37	37	75	20	20	hypothetical protein
Mfumv2_2077	987	60	43	43	75	49	49	162	159	159	Aldo/keto reductase
Mfumv2_2078	1821	144	189	189	149	177	181	210	378	379	Translation elongation factor LepA
Mfumv2_2079	1143	107	88	88	106	81	81	190	215	215	Signal peptidase I (EC 3.4.21.89)
Mfumv2_2080	171	121	15	15	246	28	28	94	16	16	hypothetical protein
Mfumv2_2081	465	60	20	20	61	19	19	137	63	63	UspA

Mfumv2_2082	1689	225	274	274	294	331	331	235	392	393	Formate--tetrahydrofolate ligase (EC 6.3.4.3)
Mfumv2_2083	1914	232	321	321	316	402	403	263	500	500	hypothetical protein
Mfumv2_2084	1050	189	143	143	232	162	162	309	322	322	Phenylalanyl-tRNA synthetase alpha chain (EC 6.1.1.20)
Mfumv2_2085	2706	280	547	547	273	492	492	343	919	920	Isoleucyl-tRNA synthetase (EC 6.1.1.5)
Mfumv2_2087	897	20546	13296	13311	24813	14821	14825	12182	10824	10836	Opacity protein or related surface antigen
Mfumv2_2088	960	261	180	181	325	208	208	247	235	235	Quinone oxidoreductase (EC 1.6.5.5)
Mfumv2_2089	669	433	209	209	451	201	201	356	236	236	Thiamin-phosphate pyrophosphorylase (EC 2.5.1.3)
Mfumv2_2090	1362	658	646	647	859	778	779	598	807	807	Biotin carboxylase of acetyl-CoA carboxylase (EC 6.3.4.14)
Mfumv2_2092	291	29	6	6	10	1	2	83	24	24	hypothetical protein
Mfumv2_2093	174	231	29	29	345	40	40	574	98	99	hypothetical protein
Mfumv2_2094	612	701	310	310	1305	532	532	1213	736	736	Translation elongation factor Ts
Mfumv2_2095	747	1114	600	601	1606	798	799	2630	1948	1948	SSU ribosomal protein S2p (SAe)
Mfumv2_2096	756	1222	664	667	1400	705	705	2570	1926	1927	hypothetical protein
Mfumv2_2097	189	886	121	121	1223	154	154	1403	263	263	hypothetical protein
Mfumv2_2098	465	143	48	48	174	54	54	132	61	61	DEAD/DEAH box helicase domain protein
Mfumv2_2099	372	402	108	108	432	107	107	409	150	151	Peroxide stress regulator; Ferric uptake regulation protein; Fe2+/Zn2+ uptake regulation proteins
Mfumv2_2101	177	31	4	4	17	2	2	17	3	3	hypothetical protein
Mfumv2_2103	1452	358	375	375	440	426	426	359	517	517	transcriptional regulator, NifA subfamily, Fis Family
Mfumv2_2104	459	271	90	90	278	84	85	299	136	136	Thioredoxin Disulfide Isomerase
Mfumv2_2105	144	77	8	8	146	14	14	147	21	21	hypothetical protein
Mfumv2_2106	387	465	130	130	671	173	173	612	235	235	hypothetical protein
Mfumv2_2107	561	276	112	112	385	144	144	431	240	240	hypothetical protein
Mfumv2_2108	930	71	48	48	77	48	48	108	100	100	glycosyl transferase, family 2
Mfumv2_2109	954	71	49	49	87	55	55	103	97	97	glycosyl transferase
Mfumv2_2110	777	87	49	49	137	71	71	384	296	296	glycosyl transferase family 2
Mfumv2_2111	786	129	73	73	145	76	76	648	505	505	polysaccharide deacetylase
Mfumv2_2112	942	69	47	47	41	26	26	49	46	46	Glycosyl transferase, family 2
Mfumv2_2113	1122	58	47	47	72	54	54	57	63	63	predicted glycosyltransferase
Mfumv2_2114	1047	131	99	99	184	128	128	126	131	131	hypothetical protein
Mfumv2_2115	774	202	113	113	151	78	78	167	128	128	3-methyl-2-oxobutanoate hydroxymethyltransferase (EC 2.1.2.11)
Mfumv2_2116	504	55	20	20	66	22	22	84	42	42	2-amino-4-hydroxy-6-hydroxymethyldihydropteridine pyrophosphokinase (EC 2.7.6.3)
Mfumv2_2117	906	44	29	29	50	30	30	61	55	55	SAM-dependent methyltransferase
Mfumv2_2118	519	267	100	100	214	74	74	220	113	113	hypothetical protein
Mfumv2_2119	780	637	359	359	627	326	326	509	394	394	polysaccharide deacetylase family protein
Mfumv2_2120	1125	121	98	98	131	98	98	653	729	729	2-methylcitrate synthase (EC 2.3.3.5)
Mfumv2_2121	207	301	45	45	334	46	46	370	76	76	hypothetical protein
Mfumv2_2122	153	18	2	2	49	5	5	73	11	11	hypothetical protein
Mfumv2_2123	1947	47	66	66	49	63	63	154	296	298	hypothetical protein
Mfumv2_2124	141	69	7	7	32	3	3	29	4	4	hypothetical protein
Mfumv2_2125	2538	121	222	222	130	219	219	146	367	367	TPR-domain containing protein
Mfumv2_2126	2844	111	227	227	141	267	267	109	307	307	Excinuclease ABC subunit A
Mfumv2_2127	780	153	86	86	198	103	103	159	123	123	RNA binding methyltransferase FtsJ like
Mfumv2_2128	159	9	1	1	9	1	1	38	6	6	hypothetical protein
Mfumv2_2129	141	29	3	3	245	23	23	315	44	44	hypothetical protein
Mfumv2_2130	750	127	69	69	264	132	132	253	188	188	hypothetical protein
Mfumv2_2131	1242	147	132	132	187	155	155	287	354	354	putative DNA modification methylase
Mfumv2_2132	1164	175	147	147	179	139	139	335	387	387	hypothetical protein
Mfumv2_2133	2145	229	355	355	249	356	356	273	580	580	FIG01147549: hypothetical protein
Mfumv2_2134	1413	110	112	112	175	165	165	198	278	278	hypothetical cytosolic protein
Mfumv2_2135	2241	1351	2109	2187	344	500	513	147	326	327	Bipolar DNA helicase

Mfumv2_2136	504	49	18	18	30	10	10	38	19	19	hypothetical protein
Mfumv2_2137	231	42	7	7	6	1	1	26	6	6	hypothetical protein
Mfumv2_2138	531	323	124	124	410	145	145	108	57	57	conserved hypothetical protein
Mfumv2_2139	1575	24	26	27	35	37	37	88	138	138	Inner membrane component of tripartite multidrug resistance system
Mfumv2_2140	1275	407	375	375	472	401	401	586	740	741	Enolase (EC 4.2.1.11)
Mfumv2_2141	375	229	62	62	304	76	76	237	87	88	Cell division protein DivIC (FtsB), stabilizes FtsL against RasP cleavage
Mfumv2_2142	1095	72	57	57	138	101	101	99	108	108	Putative coproporphyrinogen III oxidase of BS HemN-type, oxygen-independent (EC 1.3.99.22)
Mfumv2_2143	927	73	49	49	66	41	41	38	35	35	Glycosyl transferase, family 2
Mfumv2_2144	747	50	27	27	66	33	33	77	57	57	hypothetical protein
Mfumv2_2145	660	122	58	58	207	91	91	154	101	101	hypothetical protein
Mfumv2_2146	441	72	23	23	78	23	23	53	23	23	Archease
Mfumv2_2147	159	61	7	7	47	5	5	51	8	8	hypothetical protein
Mfumv2_2148	159	17	2	2	47	5	5	19	3	3	hypothetical protein
Mfumv2_2149	1431	154	159	159	274	261	261	80	112	113	RNA-2',3'-PO4:RNA-5'-OH ligase
Mfumv2_2150	114	24	2	2	0	0	0	18	2	2	hypothetical protein
Mfumv2_2151	1104	102	81	81	79	58	58	73	80	80	hypothetical protein
Mfumv2_2152	1194	36	31	31	64	49	51	97	115	115	hypothetical protein
Mfumv2_2153	1326	65	61	62	83	72	73	167	220	220	Cysteine desulfurase (EC 2.8.1.7), SufS subfamily
Mfumv2_2154	1230	346	307	307	477	391	391	147	177	179	Putative iron-sulfur cluster assembly scaffold protein for SUF system, SufE2
Mfumv2_2155	423	98	30	30	192	54	54	91	38	38	hypothetical protein
Mfumv2_2156	402	117	32	34	198	50	53	60	24	24	hypothetical protein
Mfumv2_2157	357	66	17	17	84	20	20	144	51	51	Transcription-repair coupling factor
Mfumv2_2158	3168	102	233	233	125	262	263	228	714	716	
Mfumv2_2159	990	94	67	67	138	91	91	133	131	131	Survival protein SurA precursor (Peptidyl-prolyl cis-trans isomerase SurA) (EC 5.2.1.8)
Mfumv2_2160	885	97	62	62	210	124	124	202	177	177	4-hydroxythreonine-4-phosphate dehydrogenase (EC 1.1.1.262)
Mfumv2_2161	1284	93	86	86	150	128	128	90	113	114	N-acetylglutamate synthase related protein
Mfumv2_2162	378	176	48	48	179	45	45	104	39	39	conserved hypothetical protein
Mfumv2_2163	837	99	60	60	75	42	42	113	94	94	hypothetical protein
Mfumv2_2164	117	0	0	0	13	1	1	52	6	6	hypothetical protein
Mfumv2_2165	975	703	494	495	949	616	616	1053	1018	1018	Fructose-bisphosphate aldolase class II (EC 4.1.2.13)
Mfumv2_2166	300	6438	1394	1395	3253	650	650	2810	836	836	RNP-1 like RNA-binding protein
Mfumv2_2167	789	616	351	351	510	268	268	896	701	701	Phosphoesterase family protein
Mfumv2_2168	828	105	63	63	103	57	57	111	91	91	hypothetical protein
Mfumv2_2169	369	120	32	32	85	21	21	164	60	60	hypothetical protein
Mfumv2_2170	1245	415	373	373	265	220	220	648	800	800	Aspartate aminotransferase (EC 2.6.1.1)
Mfumv2_2171	2016	37	54	54	38	51	51	128	256	256	DinG family ATP-dependent helicase YoaA
Mfumv2_2172	357	213	55	55	185	44	44	472	167	167	hypothetical protein
Mfumv2_2173	126	77	7	7	83	7	7	160	19	20	hypothetical protein
Mfumv2_2174	138	261	26	26	218	19	20	102	13	14	hypothetical protein
Mfumv2_2175	774	82	46	46	99	50	51	64	49	49	Similar to ribosomal large subunit pseudouridine synthase D, group RluD12
Mfumv2_2176	1014	93	68	68	124	84	84	157	158	158	Alcohol dehydrogenase (EC 1.1.1.1)
Mfumv2_2180	1374	754	747	748	794	727	727	565	770	770	beta-lactamase domain protein
Mfumv2_2181	1893	2239	3060	3061	3238	4080	4083	32	61	61	Cytochrome c oxidase polypeptide I (EC 1.9.3.1)
Mfumv2_2182	150	18	2	2	30	3	3	7	1	1	hypothetical protein
Mfumv2_2183	186	7	1	1	8	1	1	22	4	4	hypothetical protein
Mfumv2_2184	384	227	63	63	321	79	82	147	56	56	hypothetical protein
Mfumv2_2185	1512	234	255	255	318	320	320	311	466	466	Glucose-6-phosphate 1-dehydrogenase (EC 1.1.1.49)
Mfumv2_2186	1482	113	121	121	124	121	122	169	248	249	Exopolyphosphatase (EC 3.6.1.11)
Mfumv2_2188	156	133	15	15	289	30	30	226	35	35	hypothetical protein
Mfumv2_2189	792	94	54	54	76	40	40	224	176	176	dimethyladenosine transferase(EC:2.1.1.-)

Mfumv2_2190	2364	561	956	957	563	887	887	1003	2352	2352	5-methyltetrahydropteroyltriglutamate--homocysteine methyltransferase (EC 2.1.1.14)
Mfumv2_2191	1128	160	130	130	240	179	180	252	282	282	calcium/proton antiporter
Mfumv2_2192	2520	418	760	760	596	999	1001	438	1093	1094	Glycogen phosphorylase (EC 2.4.1.1)
Mfumv2_2193	1371	94	93	93	139	127	127	148	201	201	hypothetical protein
Mfumv2_2194	636	192	88	88	359	152	152	330	208	208	SAM-dependent methyltransferase YafE (UbiE paralog)
Mfumv2_2195	138	10	1	1	98	9	9	102	14	14	hypothetical protein
Mfumv2_2196	255	76	14	14	65	11	11	59	15	15	hypothetical protein
Mfumv2_2197	762	56	31	31	51	26	26	89	67	67	rRNA small subunit methyltransferase I
Mfumv2_2198	516	244	91	91	230	77	79	129	66	66	hypothetical protein
Mfumv2_2199	771	178	98	99	97	50	50	144	110	110	MotA/TolQ/ExbB proton channel family protein
Mfumv2_2200	426	159	47	49	81	23	23	104	44	44	Biopolymer transport protein ExbD/TolR
Mfumv2_2201	798	56	32	32	83	44	44	138	109	109	hypothetical protein
Mfumv2_2202	1176	258	219	219	481	377	377	1086	1265	1266	IMP dehydrogenase subunit
Mfumv2_2203	1563	284	321	321	343	357	357	661	1024	1024	GMP synthase [glutamine-hydrolyzing] (EC 6.3.5.2)
Mfumv2_2204	1275	301	276	277	370	314	314	462	583	584	Histidinol dehydrogenase (EC 1.1.1.23)
Mfumv2_2205	1098	1783	1413	1414	2147	1570	1570	1470	1599	1601	ATP:guanido phosphotransferase
Mfumv2_2206	2511	2012	3647	3648	3222	5386	5389	2369	5897	5900	ATP-dependent Clp protease, ATP-binding subunit ClpC
Mfumv2_2207	138	271	27	27	294	27	27	219	30	30	hypothetical protein
Mfumv2_2208	300	106	23	23	125	25	25	165	49	49	hypothetical protein
Mfumv2_2209	960	192	133	133	225	144	144	269	256	256	2-dehydropantoate 2-reductase (EC 1.1.1.169)
Mfumv2_2210	117	118	10	10	192	15	15	207	24	24	hypothetical protein
Mfumv2_2211	351	229	58	58	727	170	170	388	135	135	hypothetical protein
Mfumv2_2212	1392	327	328	329	358	331	332	266	367	367	Probable Co/Zn/Cd efflux system membrane fusion protein
Mfumv2_2213	3192	78	179	179	108	230	230	129	408	409	Cobalt-zinc-cadmium resistance protein CzcA; Cation efflux system protein CusA
Mfumv2_2214	897	131	85	85	85	51	51	208	185	185	3-hydroxyisobutyrate dehydrogenase (EC 1.1.1.31)
Mfumv2_2215	327	330	77	78	482	105	105	145	47	47	hypothetical protein
Mfumv2_2216	1083	70	55	55	67	48	48	360	383	387	predicted protein
Mfumv2_2217	777	989	555	555	1080	558	559	4262	3284	3284	hypothetical protein
Mfumv2_2218	957	41	28	28	60	38	38	110	104	104	lipopolysaccharide heptosyltransferase I
Mfumv2_2219	738	75	40	40	59	29	29	94	69	69	hypothetical protein
Mfumv2_2220	969	510	357	357	713	460	460	865	831	831	Cysteine synthase (EC 2.5.1.47)
Mfumv2_2221	861	1254	780	780	783	449	449	1075	918	918	RNA polymerase sigma factor RpoD
Mfumv2_2222	540	274	107	107	270	97	97	416	223	223	Adenine phosphoribosyltransferase (EC 2.4.2.7)
Mfumv2_2223	456	24	8	8	20	6	6	51	22	23	hypothetical protein
Mfumv2_2224	330	46	11	11	14	3	3	15	5	5	hypothetical protein
Mfumv2_2225	168	49	6	6	36	4	4	42	7	7	hypothetical protein
Mfumv2_2226	129	75	7	7	128	11	11	133	17	17	hypothetical protein
Mfumv2_2227	798	82	47	47	211	112	112	136	107	108	Acyl-[acyl-carrier-protein]--UDP-N-acetylglucosamine O-acyltransferase (EC 2.3.1.129)
Mfumv2_2228	1326	323	309	309	466	410	412	264	346	347	GTP-binding protein HflX
Mfumv2_2229	936	58	39	39	83	52	52	48	45	45	tRNA dimethylallyltransferase (EC 2.5.1.75)
Mfumv2_2230	741	50	27	27	59	29	29	33	24	24	hypothetical protein
Mfumv2_2231	1338	532	514	514	489	433	436	219	288	291	hypothetical protein
Mfumv2_2232	837	200	120	121	196	109	109	247	205	205	hypothetical protein
Mfumv2_2233	183	113	15	15	172	21	21	402	70	73	hypothetical protein
Mfumv2_2234	1818	152	200	200	176	213	213	872	1571	1572	Sulfite reductase [NADPH] flavoprotein alpha-component (EC 1.8.1.2)
Mfumv2_2235	150	18	2	2	10	1	1	94	14	14	hypothetical protein
Mfumv2_2236	1128	335	273	273	197	148	148	468	524	524	NADH-ubiquinone oxidoreductase chain H (EC 1.6.5.3)
Mfumv2_2237	1707	660	814	814	810	921	921	1161	1964	1965	NADH-ubiquinone oxidoreductase chain G (EC 1.6.5.3)
Mfumv2_2238	1371	461	456	456	945	863	863	1108	1505	1506	NADH-ubiquinone oxidoreductase chain F (EC 1.6.5.3)
Mfumv2_2239	534	407	157	157	824	292	293	1192	631	631	NADH-ubiquinone oxidoreductase chain E (EC 1.6.5.3)

Mfumv2_2240	1257	343	309	311	598	501	501	1210	1506	1508	NADH-ubiquinone oxidoreductase chain D (EC 1.6.5.3)
Mfumv2_2241	639	416	192	192	954	406	406	1168	740	740	NADH-ubiquinone oxidoreductase chain C (EC 1.6.5.3)
Mfumv2_2242	513	575	213	213	781	267	267	1014	516	516	NADH-ubiquinone oxidoreductase chain B (EC 1.6.5.3)
Mfumv2_2243	495	28	9	10	30	10	10	65	32	32	protein-tyrosine-phosphatase
Mfumv2_2244	1125	134	109	109	113	85	85	205	229	229	Biosynthetic Aromatic amino acid aminotransferase beta (EC 2.6.1.57)
Mfumv2_2245	858	90	56	56	145	83	83	230	196	196	Prephenate dehydrogenase (EC 1.3.1.12)
Mfumv2_2246	1251	50	45	45	56	47	47	118	146	147	Lipoprotein releasing system transmembrane protein LolE
Mfumv2_2247	696	175	88	88	265	122	123	233	160	161	ABC transporter related
Mfumv2_2248	885	264	169	169	273	159	161	336	290	295	Branched-chain amino acid aminotransferase (EC 2.6.1.42)
Mfumv2_2249	141	98	10	10	85	8	8	164	23	23	hypothetical protein
Mfumv2_2250	486	1080	378	379	1724	558	558	550	265	265	Nucleotide excision repair protein, with UvrB/UvrC motif
Mfumv2_2251	354	825	210	211	827	195	195	584	205	205	hypothetical protein
Mfumv2_2252	174	756	91	95	475	53	55	435	72	75	hypothetical protein
Mfumv2_2253	1416	82	84	84	58	55	55	164	230	230	6-phosphogluconate dehydrogenase, decarboxylating (EC 1.1.1.44)
Mfumv2_2254	444	577	185	185	649	171	192	375	164	165	Ribose 5-phosphate isomerase B (EC 5.3.1.6)
Mfumv2_2255	621	67	30	30	70	29	29	101	62	62	hypothetical protein
Mfumv2_2256	1191	152	131	131	126	100	100	97	113	114	hypothetical protein
Mfumv2_2257	2850	104	213	214	123	233	234	183	516	517	hypothetical protein
Mfumv2_2258	342	1494	369	369	1874	427	427	1221	414	414	hypothetical protein
Mfumv2_2259	138	0	0	0	22	2	2	44	6	6	hypothetical protein
Mfumv2_2260	864	69	43	43	52	30	30	82	70	70	Cobalt-zinc-cadmium resistance protein
Mfumv2_2261	129	43	4	4	35	3	3	70	9	9	hypothetical protein
Mfumv2_2262	135	82	7	8	89	8	8	67	9	9	hypothetical protein
Mfumv2_2263	723	1624	847	848	417	201	201	67	48	48	Nitrous oxide reductase maturation protein NosF (ATPase)
Mfumv2_2265	783	32	18	18	44	23	23	48	37	37	Nitrous oxide reductase maturation transmembrane protein NosY
Mfumv2_2266	186	22	3	3	16	2	2	0	0	0	hypothetical protein
Mfumv2_2267	810	990	579	579	764	412	412	315	253	253	hypothetical protein
Mfumv2_2268	999	523	377	377	469	311	312	189	187	187	Nitrous oxide reductase maturation protein NosD
Mfumv2_2269	165	25	3	3	55	6	6	122	20	20	hypothetical protein
Mfumv2_2270	195	107	15	15	177	23	23	378	73	73	hypothetical protein
Mfumv2_2271	810	287	168	168	749	401	404	527	420	423	thioredoxin
Mfumv2_2272	663	113	54	54	303	134	134	380	250	250	O-methyltransferase, family 3
Mfumv2_2273	873	116	73	73	186	108	108	296	256	256	Pyrroline-5-carboxylate reductase (EC 1.5.1.2)
Mfumv2_2274	507	139	51	51	201	68	68	489	245	246	Shikimate kinase I (EC 2.7.1.71)
Mfumv2_2275	1089	146	115	115	299	217	217	326	352	352	Chorismate synthase (EC 4.2.3.5)
Mfumv2_2276	639	91	41	42	113	48	48	107	68	68	Acyl-phosphate:glycerol-3-phosphate O-acyltransferase PlsY
Mfumv2_2277	888	115	74	74	171	101	101	241	212	212	Glycerol-3-phosphate dehydrogenase [NAD(P)+] (EC 1.1.1.94)
Mfumv2_2278	1851	936	1251	1251	906	1115	1117	634	1162	1164	Glucosamine--fructose-6-phosphate aminotransferase [isomerizing] (EC 2.6.1.16)
Mfumv2_2279	1308	119	112	112	115	100	100	153	197	198	Glucose-1-phosphate adenylyltransferase (EC 2.7.7.27)
Mfumv2_2280	945	103	68	70	140	88	88	232	216	217	protein of unknown function DUF52
Mfumv2_2281	1215	370	325	325	420	340	340	572	689	689	Glutamyl-tRNA synthetase (EC 6.1.1.17) @ Glutamyl-tRNA(Gln) synthetase (EC 6.1.1.24)
Mfumv2_2282	612	170	75	75	189	77	77	219	133	133	CDP-diacylglycerol--glycerol-3-phosphate 3-phosphatidyltransferase (EC 2.7.8.5)
Mfumv2_2283	1377	66	66	66	134	123	123	179	244	244	GTP-binding protein EngA
Mfumv2_2284	135	21	2	2	0	0	0	60	8	8	hypothetical protein
Mfumv2_2285	2154	56	87	87	65	90	93	88	187	187	Periplasmic beta-glucosidase (EC 3.2.1.21)
Mfumv2_2286	867	83	52	52	62	36	36	126	107	108	Transcriptional regulator
Mfumv2_2288	1947	186	261	261	268	347	348	278	536	536	Acetyl-coenzyme A synthetase (EC 6.2.1.1)
Mfumv2_2289	363	168	44	44	58	14	14	222	80	80	hypothetical protein
Mfumv2_2290	156	0	0	0	0	0	0	19	2	3	hypothetical protein
Mfumv2_2291	117	107	9	9	38	3	3	103	12	12	hypothetical protein

Mfumv2_2292	651	276	129	130	392	169	170	406	262	262	hypothetical protein
Mfumv2_2293	423	59	18	18	106	30	30	172	72	72	hypothetical protein
Mfumv2_2294	1005	241	175	175	251	168	168	366	365	365	Methionyl-tRNA formyltransferase (EC 2.1.2.9)
Mfumv2_2296	981	227	161	161	129	84	84	88	86	86	Sua5 YciO YrdC YwlC family protein
Mfumv2_2298	2607	681	1280	1283	699	1213	1213	442	1143	1143	DNA gyrase subunit A (EC 5.99.1.3)
Mfumv2_2299	2523	678	1234	1235	906	1523	1523	605	1511	1513	DNA gyrase subunit B (EC 5.99.1.3)
Mfumv2_2300	705	120	61	61	164	77	77	112	77	78	Phosphate regulon transcriptional regulatory protein PhoB (SphR)
Mfumv2_2301	1305	36	34	34	44	38	38	63	81	81	Osmosensitive K+ channel histidine kinase KdpD (EC 2.7.3.-)
Mfumv2_2302	747	82	44	44	105	52	52	108	80	80	Uncharacterized conserved protein
Mfumv2_2303	807	170	99	99	127	68	68	124	99	99	hypothetical protein
Mfumv2_2304	2184	456	713	720	571	829	831	418	904	905	Thymidylate kinase (EC 2.7.4.9)
Mfumv2_2306	840	46	28	28	23	13	13	68	57	57	hypothetical protein
Mfumv2_2307	1635	4612	5441	5446	5724	6233	6234	14096	22850	22854	Heat shock protein 60 family chaperone GroEL
Mfumv2_2308	300	6960	1508	1508	8798	1755	1758	7217	2146	2147	Heat shock protein 60 family co-chaperone GroES
Mfumv2_2309	1953	3014	4249	4252	2860	3719	3721	3391	6565	6567	Chaperone protein DnaK
Mfumv2_2310	1431	2021	2065	2089	580	553	553	605	859	859	UDP-N-acetylmuramate:L-alanyl-gamma-D-glutamyl-meso-diaminopimelate ligase (EC 6.3.2.-)
Mfumv2_2311	717	270	139	140	343	164	164	329	234	234	hypothetical protein
Mfumv2_2312	681	65	32	32	53	24	24	53	36	36	tRNA-specific adenosine-34 deaminase (EC 3.5.4.-)
Mfumv2_2313	879	564	358	358	236	138	138	335	292	292	Folate-dependent protein for Fe/S cluster synthesis/repair in oxidative stress
Mfumv2_2314	150	369	40	40	601	60	60	612	91	91	hypothetical protein
Mfumv2_2315	453	547	178	179	719	217	217	837	376	376	hypothetical protein
Mfumv2_2316	324	111	26	26	37	7	8	162	52	52	Tetratricopeptide TPR_2 repeat protein
Mfumv2_2317	1539	194	215	216	296	303	303	370	564	564	Uroporphyrinogen-III methyltransferase (EC 2.1.1.107) / Uroporphyrinogen-III synthase (EC 4.2.1.75)
Mfumv2_2318	765	311	171	172	532	271	271	464	351	352	Porphobilinogen deaminase (EC 2.5.1.61)
Mfumv2_2319	1065	108	83	83	185	131	131	277	293	293	Glutamyl-tRNA reductase (EC 1.2.1.70)
Mfumv2_2320	810	209	121	122	217	113	117	225	180	181	HemX protein, negative effector of steady-state concentration of glutamyl-tRNA reductase
Mfumv2_2321	447	1193	385	385	1165	346	347	395	175	175	hypothetical protein
Mfumv2_2322	1119	214	173	173	177	132	132	246	272	273	permease YjgP/YjgQ family protein
Mfumv2_2323	171	445	54	55	509	58	58	507	86	86	hypothetical protein
Mfumv2_2325	1104	100	80	80	152	112	112	346	379	379	Xaa-Pro aminopeptidase (EC 3.4.11.9)
Mfumv2_2326	1659	96	115	115	77	85	85	123	202	202	Outer membrane component of tripartite multidrug resistance system
Mfumv2_2327	5505	77	307	307	110	402	403	93	505	509	Excinuclease ABC subunit A, dimeric form
Mfumv2_2328	1620	176	206	206	201	217	217	202	325	325	Pyruvate decarboxylase (EC 4.1.1.1); Alpha-keto-acid decarboxylase (EC 4.1.1.-)
Mfumv2_2329	681	91	45	45	163	74	74	219	148	148	DNA-binding heavy metal response regulator
Mfumv2_2330	972	85	60	60	80	52	52	104	100	100	hypothetical protein
Mfumv2_2331	702	418	212	212	483	226	226	389	271	271	hypothetical protein
Mfumv2_2332	2763	73	122	146	56	92	103	94	235	257	hypothetical protein
Mfumv2_2334	2157	108	167	168	131	188	188	180	384	385	Phytoene synthase (EC 2.5.1.32)
Mfumv2_2335	978	144	101	102	200	130	130	313	304	304	Deoxyhypusine synthase (EC 2.5.1.46)
Mfumv2_2336	2052	276	407	409	319	436	436	488	989	992	Pyrophosphate-energized proton pump (EC 3.6.1.1)
Mfumv2_2337	195	71	10	10	77	9	10	145	28	28	hypothetical protein
Mfumv2_2338	1425	331	341	341	239	227	227	489	690	691	NADH-ubiquinone oxidoreductase chain N (EC 1.6.5.3)
Mfumv2_2339	1467	403	427	427	232	226	227	386	562	562	NADH-ubiquinone oxidoreductase chain M (EC 1.6.5.3)
Mfumv2_2341	1815	601	786	788	512	619	619	458	824	825	NADH-ubiquinone oxidoreductase chain L (EC 1.6.5.3)
Mfumv2_2342	306	158	35	35	240	49	49	432	131	131	NADH-ubiquinone oxidoreductase chain K (EC 1.6.5.3)
Mfumv2_2343	498	297	107	107	410	136	136	626	309	309	NADH-ubiquinone oxidoreductase chain J (EC 1.6.5.3)
Mfumv2_2344	519	363	136	136	596	206	206	713	365	367	NADH-ubiquinone oxidoreductase chain I (EC 1.6.5.3)
Mfumv2_2345	120	173	15	15	525	42	42	109	13	13	hypothetical protein
Mfumv2_2346	432	58	18	18	17	5	5	21	9	9	HNH endonuclease
Mfumv2_2347	2520	28	51	51	17	29	29	30	76	76	Outer membrane receptor protein, mostly Fe transport

Mfumv2_2348	1029	1503	1114	1117	1936	1326	1327	1168	1192	1192	aldo/keto reductase
Mfumv2_2349	315	325	74	74	581	122	122	410	128	128	conserved hypothetical protein
Mfumv2_2350	564	268	109	109	657	246	247	474	265	265	putative protease
Mfumv2_2351	606	311	136	136	416	168	168	458	275	275	hypothetical protein
Mfumv2_2352	606	208	91	91	248	100	100	193	116	116	CDP-diacylglycerol--glycerol-3-phosphate 3-phosphatidyltransferase (EC 2.7.8.5)
Mfumv2_2353	339	351	86	86	456	102	103	262	88	88	hypothetical protein
Mfumv2_2354	624	62	28	28	79	33	33	100	62	62	cytochrome c family protein
Mfumv2_2355	567	73	30	30	29	11	11	64	36	36	Intracellular proteinase inhibitor
Mfumv2_2356	978	125	87	88	318	207	207	154	149	149	UDP-glucose 4-epimerase (EC 5.1.3.2)
Mfumv2_2357	1269	89	82	82	122	103	103	133	167	167	Glycosyltransferase
Mfumv2_2358	2424	37	64	64	54	87	87	42	101	101	Outer membrane receptor protein, mostly Fe transport
Mfumv2_2359	1026	108	80	80	183	125	125	235	238	239	Ribosomal small subunit Rsm22
Mfumv2_2360	180	38	5	5	0	0	0	84	15	15	hypothetical protein
Mfumv2_2361	2619	55	103	104	42	73	73	72	185	186	Outer membrane receptor protein, mostly Fe transport
Mfumv2_2362	1209	62	53	54	87	68	70	37	44	44	BNR repeat domain protein
Mfumv2_2363	2223	50	80	80	39	58	58	40	89	89	TonB-dependent receptor
Mfumv2_2364	1317	86	82	82	96	84	84	160	208	209	UDP-N-acetylmuramoylalanine--D-glutamate ligase (EC 6.3.2.9)
Mfumv2_2365	1425	96	99	99	143	135	136	154	217	217	hypothetical protein
Mfumv2_2366	744	154	83	83	224	111	111	248	182	183	Glucose-1-phosphate thymidylyltransferase (EC 2.7.7.24)
Mfumv2_2367	1764	122	154	155	251	295	295	420	734	735	Phosphoenolpyruvate-protein phosphotransferase of PTS system (EC 2.7.3.9)
Mfumv2_2368	318	435	100	100	788	167	167	688	217	217	Phosphotransferase system, phosphocarrier protein HPr
Mfumv2_2369	981	168	119	119	375	245	245	636	619	619	HPr kinase/phosphorylase (EC 2.7.1.-) (EC 2.7.4.-)
Mfumv2_2370	1110	102	81	82	130	96	96	254	280	280	Para-aminobenzoate synthase, aminase component (EC 2.6.1.85)
Mfumv2_2371	1161	750	629	629	1549	1198	1198	1293	1487	1489	Chaperone protein DnaJ
Mfumv2_2372	615	610	271	271	820	336	336	635	387	387	Heat shock protein GrpE
Mfumv2_2373	1242	448	401	402	521	431	431	510	625	628	Serine phosphatase RsbU, regulator of sigma subunit
Mfumv2_2374	528	684	261	261	899	314	316	1112	582	582	anti-sigma-factor antagonist
Mfumv2_2375	189	51	7	7	32	4	4	53	10	10	hypothetical protein
Mfumv2_2376	795	78	45	45	115	60	61	250	196	197	Metal-dependent hydrolases of the beta-lactamase superfamily I; PhnP protein
Mfumv2_2377	807	50	29	29	89	48	48	199	159	159	Putative deoxyribonuclease YcfH
Mfumv2_2378	1767	547	696	698	602	705	708	698	1223	1223	Dihydroxy-acid dehydratase (EC 4.2.1.9)
Mfumv2_2379	1356	193	189	189	165	149	149	161	216	216	tRNA:m(5)U-54 MTase gid
Mfumv2_2380	2328	1927	3224	3240	1213	1878	1881	1587	3654	3663	Phenylalanyl-tRNA synthetase beta chain (EC 6.1.1.20)
Mfumv2_2381	771	2855	1589	1590	2344	1204	1204	807	617	617	hypothetical protein
Mfumv2_2382	645	406	189	189	333	143	143	269	172	172	tRNA (guanine46-N7-)-methyltransferase (EC 2.1.1.33)
Mfumv2_2383	381	280	76	77	221	56	56	146	55	55	Dimeric dUTPase (EC 3.6.1.23)
Mfumv2_2384	447	28	9	9	24	7	7	63	28	28	hypothetical protein
Mfumv2_2386	753	191	104	104	291	146	146	257	191	192	glycosyl transferase, family 2
Mfumv2_2387	789	68	39	39	57	30	30	180	141	141	putative polysaccharide deacetylase
Mfumv2_2388	1170	95	79	80	199	149	155	239	274	277	hypothetical protein
Mfumv2_2389	825	1178	700	702	511	280	281	497	407	407	Imidazole glycerol phosphate synthase cyclase subunit (EC 4.1.3.-)
Mfumv2_2390	495	92	33	33	88	29	29	147	71	72	tRNA (cytidine(34)-2'-O)-methyltransferase (EC 2.1.1.207) ## TrmL
Mfumv2_2391	462	126	42	42	91	28	28	301	138	138	hypothetical protein
Mfumv2_2392	774	84	47	47	111	57	57	278	213	213	tRNA pseudouridine synthase A (EC 4.2.1.70)
Mfumv2_2393	684	391	193	193	590	269	269	510	346	346	hypothetical protein
Mfumv2_2394	441	1435	457	457	2434	713	715	1694	741	741	Putative Holliday junction resolvase YggF
Mfumv2_2395	822	123	73	73	161	88	88	166	135	135	Tryptophan synthase alpha chain (EC 4.2.1.20)
Mfumv2_2396	219	6	1	1	34	5	5	23	5	5	hypothetical protein
Mfumv2_2397	4560	600	1974	1975	995	3021	3022	1022	4620	4622	Glutamate synthase [NADPH] large chain (EC 1.4.1.13)
Mfumv2_2398	390	653	184	184	805	208	209	729	282	282	hypothetical protein

Mfumv2_2399	924	33	22	22	63	38	39	61	54	56	methyltransferase FkbM family
Mfumv2_2400	903	100	65	65	141	84	85	238	213	213	Dihydropteroate synthase (EC 2.5.1.15)
Mfumv2_2401	612	267	118	118	314	128	128	391	237	237	Imidazoleglycerol-phosphate dehydratase (EC 4.2.1.19)
Mfumv2_2402	123	225	20	20	159	13	13	172	21	21	hypothetical protein
Mfumv2_2403	624	75	34	34	132	55	55	163	101	101	hypothetical protein
Mfumv2_2404	504	165	60	60	128	43	43	166	82	83	hypothetical protein
Mfumv2_2405	1158	103	86	86	113	87	87	175	201	201	hypothetical protein
Mfumv2_2406	153	0	0	0	29	3	3	46	7	7	hypothetical protein
Mfumv2_2407	738	3446	1758	1837	269	128	132	119	85	87	hypothetical protein
Mfumv2_2408	1497	136	147	147	163	163	163	147	218	218	Aldehyde dehydrogenase (EC 1.2.1.3)
Mfumv2_2409	846	31	19	19	41	23	23	44	37	37	hypothetical protein
Mfumv2_2410	984	23	16	16	40	26	26	41	40	40	DegT/DnrJ/EryC1/StrS aminotransferase
Mfumv2_2411	150	46	5	5	80	8	8	47	7	7	hypothetical protein
Mfumv2_2412	126	55	5	5	95	8	8	224	28	28	hypothetical protein
Mfumv2_2413	864	88	55	55	137	79	79	205	176	176	2-polyprenylphenol hydroxylase and related flavodoxin oxidoreductases
Mfumv2_2414	1134	126	102	103	352	265	266	255	287	287	Ferredoxin
Mfumv2_2415	1020	119	88	88	274	186	186	180	182	182	Putative dihydropyrimidine dehydrogenase [NADP+], similar to dihydroorotate dehydrogenase
Mfumv2_2416	3549	112	286	286	203	479	481	86	304	304	Pyruvate-flavodoxin oxidoreductase (EC 1.2.7.-)
Mfumv2_2417	1149	163	135	135	280	214	214	149	170	170	Ferredoxin
Mfumv2_2418	462	75	25	25	136	41	42	85	39	39	cAMP-binding proteins, catabolite gene activator and regulatory subunit of cAMP-dependent protein kinases
Mfumv2_2419	846	110	67	67	133	74	75	103	86	86	Dihydroorotate dehydrogenase electron transfer subunit (EC 1.3.3.1)
Mfumv2_2420	882	68	43	43	95	56	56	104	91	91	Coenzyme F420-reducing hydrogenase, gamma subunit
Mfumv2_2421	1296	63	59	59	98	85	85	90	116	116	hydrogenase/sulfur reductase, alpha subunit
Mfumv2_2422	1818	75	98	98	105	127	127	303	545	546	Pyruvate kinase family protein
Mfumv2_2423	129	140	13	13	186	15	16	164	21	21	hypothetical protein
Mfumv2_2424	138	40	4	4	54	5	5	88	12	12	hypothetical protein
Mfumv2_2425	2721	65	128	128	65	115	117	93	246	250	hypothetical protein
Mfumv2_2429	891	154	99	99	276	164	164	243	215	215	Glucose-1-phosphate thymidylyltransferase (EC 2.7.7.24)
Mfumv2_2430	1089	156	123	123	252	183	183	361	390	390	dTDP-glucose 4,6-dehydratase (EC 4.2.1.46)
Mfumv2_2433	165	235	18	28	209	14	23	238	23	39	hypothetical protein
Mfumv2_2434	645	133	62	62	158	68	68	291	186	186	Riboflavin synthase eubacterial/eukaryotic (EC 2.5.1.9)
Mfumv2_2435	729	929	485	489	962	466	467	7723	5569	5583	putative dihydropteridine reductase
Mfumv2_2436	984	45	32	32	61	40	40	128	124	125	hypothetical protein
Mfumv2_2437	2085	31	46	46	21	29	29	46	94	95	DNA internalization-related competence protein ComEC/Rec2
Mfumv2_2438	762	142	78	78	160	81	81	172	130	130	Rhodanese domain protein
Mfumv2_2439	1986	116	165	167	230	304	304	470	925	925	tRNA uridine 5-carboxymethylaminomethyl modification enzyme GidA
Mfumv2_2440	1458	102	107	107	137	133	133	267	386	386	replicative DNA helicase
Mfumv2_2441	795	251	143	144	289	153	153	377	297	297	Thiazole biosynthesis protein ThiG
Mfumv2_2442	849	333	204	204	483	273	273	251	211	211	Menaquinone via futalosine step 4
Mfumv2_2443	945	274	187	187	539	338	339	751	703	704	Acetylspermidine deacetylase (EC 3.5.1.48); Deacetylases, including yeast histone deacetylase and acetoin utilization protein
Mfumv2_2444	141	226	23	23	75	7	7	164	23	23	hypothetical protein
Mfumv2_2446	501	373	135	135	285	95	95	711	351	353	Cytochrome c family protein
Mfumv2_2447	135	92	9	9	100	9	9	261	34	35	hypothetical protein
Mfumv2_2448	906	38	25	25	91	55	55	118	106	106	Homoserine kinase (EC 2.7.1.39)
Mfumv2_2449	810	164	96	96	163	86	88	112	90	90	FIG056164: rhomboid family serine protease
Mfumv2_2450	885	58	37	37	90	53	53	136	119	119	Quinolinate phosphoribosyltransferase [decarboxylating] (EC 2.4.2.19)
Mfumv2_2451	801	166	96	96	291	154	155	481	374	382	PATAN domain containing protein
Mfumv2_2452	534	443	171	171	545	194	194	763	404	404	hypothetical protein
Mfumv2_2453	375	1407	379	381	1693	423	423	1124	418	418	hypothetical protein

Mfumv2_2454	858	934	577	579	922	526	527	1717	1461	1461	ATP synthase A chain (EC 3.6.3.14)
Mfumv2_2455	213	598	92	92	1071	150	152	3972	839	839	ATP synthase C chain (EC 3.6.3.14)
Mfumv2_2456	531	1494	572	573	2013	711	712	3815	2009	2009	ATP synthase B chain (EC 3.6.3.14)
Mfumv2_2457	396	392	112	112	603	159	159	1826	717	717	ATP synthase delta chain (EC 3.6.3.14)
Mfumv2_2458	1554	981	1099	1101	876	905	907	2286	3518	3522	ATP synthase alpha chain (EC 3.6.3.14)
Mfumv2_2459	807	518	302	302	560	301	301	1533	1226	1227	ATP synthase gamma chain (EC 3.6.3.14)
Mfumv2_2460	1404	1099	1112	1114	852	796	797	1819	2533	2533	ATP synthase beta chain (EC 3.6.3.14)
Mfumv2_2461	438	809	256	256	1248	364	364	1421	617	617	ATP synthase epsilon chain (EC 3.6.3.14)
Mfumv2_2462	306	176	39	39	265	54	54	484	147	147	protein of unknown function DUF971
Mfumv2_2463	936	104	70	70	197	123	123	637	590	591	tRNA(Ile)-lysidine synthetase
Mfumv2_2464	753	70	38	38	78	39	39	135	101	101	Ribosomal large subunit pseudouridine synthase B (EC 4.2.1.70)
Mfumv2_2465	1395	87	88	88	82	73	76	153	211	211	Argininosuccinate lyase (EC 4.3.2.1)
Mfumv2_2466	1686	108	131	131	112	126	126	285	476	476	hypothetical protein
Mfumv2_2467	162	43	5	5	9	1	1	56	9	9	hypothetical protein
Mfumv2_2468	1248	82	73	74	171	142	142	569	703	704	3,4-dihydroxy-2-butanone 4-phosphate synthase (EC 4.1.99.12) / GTP cyclohydrolase II (EC 3.5.4.25)
Mfumv2_2469	738	62	33	33	126	62	62	238	173	174	hypothetical protein
Mfumv2_2470	609	130	57	57	158	61	64	341	204	206	hypothetical protein
Mfumv2_2471	972	130	91	91	269	174	174	299	288	288	hypothetical protein
Mfumv2_2472	2148	150	233	233	367	522	525	282	598	600	Hydroxylamine oxidoreductase precursor (EC 1.7.3.4)
Mfumv2_2473	252	82	15	15	83	12	14	40	10	10	hypothetical protein
Mfumv2_2474	504	102	37	37	155	52	52	110	55	55	hypothetical protein
Mfumv2_2475	114	0	0	0	40	3	3	142	16	16	hypothetical protein
Mfumv2_2476	114	85	7	7	171	13	13	168	19	19	hypothetical protein
Mfumv2_2477	126	231	21	21	131	11	11	32	4	4	hypothetical protein
Mfumv2_2478	186	15	2	2	0	0	0	43	8	8	hypothetical protein
Mfumv2_2479	678	143	69	70	113	51	51	159	107	107	hypothetical protein
Mfumv2_2480	516	80	30	30	105	35	36	115	59	59	Putative Nudix hydrolase YfcD (EC 3.6.-.-)
Mfumv2_2481	627	148	67	67	108	45	45	108	67	67	COG1272: Predicted membrane protein hemolysin III homolog
Mfumv2_2482	1170	130	109	110	151	117	118	179	208	208	AttH component of AttEFGH ABC transport system
Mfumv2_2483	2544	72	131	132	74	124	125	98	248	248	protein of unknown function DUF214
Mfumv2_2484	678	353	172	173	341	154	154	464	310	312	ABC transporter related
Mfumv2_2485	1350	184	178	179	256	230	230	213	285	285	hypothetical protein
Mfumv2_2486	945	47	32	32	95	59	60	155	145	145	hypothetical protein
Mfumv2_2487	735	107	57	57	84	40	41	110	80	80	MotA/TolQ/ExbB proton channel family protein
Mfumv2_2488	474	137	47	47	158	50	50	89	42	42	Biopolymer transport protein ExbD/TolR
Mfumv2_2489	798	76	44	44	73	37	39	96	76	76	Periplasmic protein TonB
Mfumv2_2490	183	23	3	3	131	16	16	88	16	16	hypothetical protein
Mfumv2_2491	1146	57	47	47	113	85	86	121	137	137	tolB protein precursor, periplasmic protein involved in the tonb-independent uptake of group A colicins
Mfumv2_2492	540	128	49	50	183	65	66	286	152	153	18K peptidoglycan-associated outer membrane lipoprotein; Peptidoglycan-associated lipoprotein precursor; Outer membrane protein P6; OmpA/MotB precursor
Mfumv2_2493	1431	146	147	151	164	156	156	181	256	257	ATPase, AAA family
Mfumv2_2494	1032	30	22	22	49	34	34	65	67	67	BatB
Mfumv2_2495	756	123	67	67	214	108	108	177	133	133	hypothetical protein
Mfumv2_2496	2613	87	165	165	111	194	194	125	325	325	hypothetical protein
Mfumv2_2497	1167	184	155	155	347	270	270	353	409	409	hypothetical protein
Mfumv2_2498	309	1290	286	288	1526	314	314	434	133	133	Ferredoxin subunits of nitrite reductase and ring-hydroxylating dioxygenases
Mfumv2_2499	1038	496	372	372	683	472	472	413	425	425	ErfK/YbiS/YcfS/YnhG family protein
Mfumv2_2500	759	268	147	147	439	220	222	896	672	674	Iron-sulfur cluster assembly ATPase protein SufC
Mfumv2_2501	1425	549	563	565	813	771	772	565	797	798	Iron-sulfur cluster assembly protein SufB
Mfumv2_2502	1293	502	467	469	561	482	483	595	763	763	Iron-sulfur cluster assembly protein SufD

Mfumv2_2503	555	1509	605	605	1745	645	645	1072	590	590	PaaD-like protein (DUF59) involved in Fe-S cluster assembly
Mfumv2_2504	1287	205	190	191	260	222	223	205	261	261	hypothetical protein
Mfumv2_2505	579	442	185	185	547	211	211	246	141	141	hypothetical protein
Mfumv2_2506	1098	52	41	41	81	59	59	91	99	99	NAD-dependent epimerase/dehydratase
Mfumv2_2507	1137	74	61	61	108	82	82	169	191	191	Cysteine desulfurase (EC 2.8.1.7)
Mfumv2_2508	873	102	64	64	175	102	102	198	171	171	Serine acetyltransferase (EC 2.3.1.30)
Mfumv2_2509	900	192	125	125	194	116	116	238	212	212	Shikimate 5-dehydrogenase I alpha (EC 1.1.1.25)

Supplementary Table S5 | RNA-Seq analysis of house keeping genes of *Methylacidiphilum fumariolicum* SolV grown under different conditions

			H_2/NH_4^+ bioreactor		CH_4/NO_3^- bioreactor		Cells at µMax		
GenBank identifier	Gene length	Gene name	RPKM	Unique reads	RPKM	Unique reads	RPKM	Unique reads	Annotation
Mfumv2_0001	1356	dnaA	223	201	243	238	216	290	Chromosomal replication initiator protein DnaA
Mfumv2_0002	1116	dnaN	287	213	262	209	333	366	DNA polymerase III beta subunit (EC 2.7.7.7)
Mfumv2_0008	1101		82	60	78	62	121	132	ATP-dependent DNA ligase
Mfumv2_0010	1269	purA	291	246	256	235	289	363	Adenylosuccinate synthetase (EC 6.3.4.4)
Mfumv2_0015	1134	kamA	405	319	396	338	891	1042	Lysine 2,3-aminomutase (EC 5.4.3.2)
Mfumv2_0021	1803	mutL	142	171	198	256	178	319	DNA mismatch repair protein mutL
Mfumv2_0022	1101		314	230	252	200	225	246	putative Predicted GTPase, translation factor/GTP-binding and nucleic acid-binding protein YchF
Mfumv2_0025	1242	wecE	340	281	246	221	391	482	Predicted pyridoxal phosphate-dependent enzymeinvolved in regulation of cell wall biogenesis
Mfumv2_0044	1704	recN	229	260	138	170	273	461	DNA repair protein ATPase, RecN
Mfumv2_0049	429	hisI	504	144	310	96	519	221	Phosphoribosyl-AMP cyclohydrolase (EC 3.5.4.19)
Mfumv2_0079	1170	alr	139	107	135	114	254	293	Alanine racemase (EC 5.1.1.1)
Mfumv2_0085	1530	metG	215	219	162	179	260	394	Methionyl-tRNA synthetase (EC 6.1.1.10)
Mfumv2_0087	1218	hisS	264	214	249	219	681	821	Histidyl-tRNA synthetase (EC 6.1.1.21)
Mfumv2_0088	1824	aspS	336	408	527	692	486	878	Aspartyl-tRNA synthetase (EC 6.1.1.12) @ Aspartyl-tRNA(Asn) synthetase (EC 6.1.1.23)
Mfumv2_0089	1431	pykF	336	320	345	357	433	613	Pyruvate kinase (EC 2.7.1.40)
Mfumv2_0114	366	cdc	82	20	87	23	58	21	ATP-dependent DNA ligase (EC 6.5.1.1) clustered with Ku protein, LigD
Mfumv2_0124	1332	purB	423	375	390	375	360	476	Adenylosuccinate lyase (EC 4.3.2.2)
Mfumv2_0129	348		380	88	366	91	519	179	Phenylpyruvate tautomerase family protein/Possible ATLS1-like light-inducible protein
Mfumv2_0130	432	rplM	956	275	695	217	3364	1441	50S ribosomal protein L13/LSU ribosomal protein L13p (L13Ae)
Mfumv2_0131	405	rpsI	1676	452	663	194	3772	1515	30S ribosomal protein S9/SSU ribosomal protein S9p (S16e)
Mfumv2_0132	1038	argC	334	230	344	258	1261	1297	N-acetyl-gamma-glutamyl-phosphate reductase (EC 1.2.1.38)
Mfumv2_0133	1215	argJ	711	574	471	413	965	1163	Glutamate N-acetyltransferase (EC 2.3.1.35) / N-acetylglutamate synthase (EC 2.3.1.1)
Mfumv2_0134	891	argB	497	295	463	298	790	698	Acetylglutamate kinase (EC 2.7.2.8)
Mfumv2_0140	1665	glgA	255	283	195	235	172	284	Glycogen synthase, ADP-glucose transglucosylase (EC 2.4.1.21)
Mfumv2_0144	2514	mutS	94	158	56	101	123	307	DNA mismatch repair protein mutS
Mfumv2_0148	1014	gppA	210	142	164	120	363	364	Exopolyphosphatase (EC 3.6.1.11)
Mfumv2_0172	1326	thdF	119	105	69	65	95	125	GTPase and tRNA-U34 5-formylation enzyme TrmE
Mfumv2_0194	1326	pcnB	228	202	172	165	222	293	tRNA nucleotidyltransferase (EC 2.7.7.21) (EC 2.7.7.25)
Mfumv2_0196	2835	gcvP	206	389	270	549	381	1068	Glycine dehydrogenase [decarboxylating] (glycine cleavage system P protein) (EC 1.4.4.2)
Mfumv2_0197	390	gcvH	505	115	547	135	501	170	Glycine cleavage system H protein
Mfumv2_0230	984		195	128	236	168	485	473	FIG146085: 3'-to-5' oligoribonuclease A, Bacillus type
Mfumv2_0234	918		378	231	297	197	172	157	Fructose-bisphosphate aldolase, archaeal class I (EC 4.1.2.13)
Mfumv2_0250	267	rpsO	267	136	208	137	130	111	30S ribosomal protein S15
Mfumv2_0253	1092	pfkA	683	221	464	163	1145	552	6-phosphofructokinase/bacterioferritin comigratory protein homolog
Mfumv2_0256	1440	pepB	800	765	512	532	565	807	Cytosol aminopeptidase PepA (EC 3.4.11.1)
Mfumv2_0264	1965	speA	290	379	240	341	524	1020	Biosynthetic arginine decarboxylase (EC 4.1.1.19)
Mfumv2_0269	777	panC	75	39	59	33	119	92	Pantoate--beta-alanine ligase (EC 6.3.2.1)
Mfumv2_0270	1032	amiC	481	323	335	244	392	392	N-acetylmuramoyl-L-alanine amidase (EC 3.5.1.28)
Mfumv2_0276	1221	lysC	1070	869	981	865	873	1057	Aspartokinase (EC 2.7.2.4)
Mfumv2_0317	828	trpC	742	408	2160	1281	794	650	Indole-3-glycerol phosphate synthase (EC 4.1.1.48)
Mfumv2_0318	654	trpF	391	150	214	89	425	243	Phosphoribosylanthranilate isomerase (EC 5.3.1.24)
Mfumv2_0319	1221	trpB	514	115	227	55	459	153	Tryptophan synthase beta chain (EC 4.2.1.20)
Mfumv2_0351	480		26	7	31	9	55	22	putative Cytochrome c, class I
Mfumv2_0358	339		1130	262	1468	369	496	171	Ribosome-associated protein Y (PSrp-1)/Ribosome hibernation protein YhbH
Mfumv2_0363	1848	thrS	555	697	1227	1671	506	949	Threonyl-tRNA synthetase (EC 6.1.1.3)

Mfumv2_0364	618	infC	1173	483	1001	446	932	570	Translation initiation factor IF-3
Mfumv2_0365	363	rplT	2275	250	1536	183	1962	321	50S ribosomal protein L20
Mfumv2_0377	657		372	130	158	60	177	92	Cytochrome c3 /Molybdopterin oxidoreductase subunit, predicted; chaperone protein HtpG
Mfumv2_0381	618	cccA	248	102	181	80	302	184	Cytochrome c family protein/ABC-type Fe3+ transport system protein
Mfumv2_0384	1446	ccoN	118	114	90	94	88	126	Cytochrome c oxidase subunit CcoN (EC 1.9.3.1)
Mfumv2_0385	597	ccoO	327	129	366	158	157	93	Cytochrome c oxidase subunit CcoO (EC 1.9.3.1)
Mfumv2_0386	639	cccA	265	113	139	64	227	144	cytochrome c, class I
Mfumv2_0387	1848	cyoB	795	977	736	982	766	1404	Cytochrome c oxidase polypeptide I (EC 1.9.3.1)
Mfumv2_0388	792	cyoC	1295	682	1439	823	763	599	Cytochrome c oxidase polypeptide III (EC 1.9.3.1)
Mfumv2_0392	792	cyoA	1526	805	883	501	532	418	Alternative cytochrome c oxidase polypeptide CoxM (EC 1.9.3.1)
Mfumv2_0414	822	dapF	468	256	296	176	248	202	Diaminopimelate epimerase (EC 5.1.1.7)
Mfumv2_0415	906	dapA	492	297	466	304	552	495	Dihydrodipicolinate synthase (EC 4.2.1.52)
Mfumv2_0461	2064	ftsH	2075	2827	1466	2165	1842	3742	ATP-dependent zinc metalloprotease FtsH 2/Cell division protein FtsH (EC 3.4.24.-)
Mfumv2_0499	1032	mauG	662	454	789	586	615	629	Cytochrome c551 peroxidase (EC 1.11.1.5)
Mfumv2_0502	1887	pol	421	518	233	309	313	582	DNA polymerase IV fused to PHP phosphatase/DNA polymerase X family
Mfumv2_0528	1161	leuA	47	35	32	27	147	169	Homocitrate synthase (EC 2.3.3.14)
Mfumv2_0556	1149	wecE	26	20	57	47	111	127	Pyridoxal phosphate-dependent enzyme/DegT/DnrJ/EryC1/StrS aminotransferase
Mfumv2_0574	891	exo	82	48	112	71	211	183	5'-3' exonuclease/DNA polymerase I (EC 2.7.7.7)
Mfumv2_0580	603	ruvA	73	31	83	38	92	58	Holliday junction DNA helicase RuvA
Mfumv2_0581	1017	ruvB	128	87	125	90	131	132	Holliday junction DNA helicase RuvB
Mfumv2_0588	645	deoC	147	63	105	49	158	101	Deoxyribose-phosphate aldolase (EC 4.1.2.4)
Mfumv2_0601	678	rpe	204	92	176	85	199	134	Ribulose-phosphate 3-epimerase (EC 5.1.3.1)
Mfumv2_0626	561	frr	794	326	567	253	335	204	Ribosome-recycling factor
Mfumv2_0628	1146	dadA	161	122	151	123	148	168	Glycine/D-amino acid oxidase (Deaminating)/FAD dependent oxidoreductase
Mfumv2_0637	1365	potE	231	210	232	229	151	205	Amino acid transporter/Amino acid permease
Mfumv2_0640	525		66	23	42	16	207	108	RNA polymerase sigma-70 factor, ECF subfamily
Mfumv2_0661	1488	trpE	120	119	89	96	191	282	Anthranilate synthase, aminase component (EC 4.1.3.27)
Mfumv2_0662	606	trpG	297	119	224	98	358	215	Anthranilate synthase, amidotransferase component (EC 4.1.3.27)
Mfumv2_0668	2481	leuS	251	414	258	462	510	1255	Leucyl-tRNA synthetase (EC 6.1.1.4)
Mfumv2_0669	1344	tldD	99	89	63	61	128	171	Predicted Zn-dependent protease, modulator of DNAgyrase, PmbA protein
Mfumv2_0670	1524	tldD	277	292	199	228	150	235	Predicted Zn-dependent protease, modulator of DNAgyrase, TldD protein
Mfumv2_0671	1248	argE	899	747	700	631	673	833	Acetylornithine deacetylase (EC 3.5.1.16)
Mfumv2_0691	243	rpmE	1800	241	778	113	933	186	50S ribosomal protein L31
Mfumv2_0696	678	nth	396	179	280	137	509	341	Endonuclease III (EC 4.2.99.18)
Mfumv2_0697	1338	rimO	428	371	266	251	406	524	Ribosomal protein S12p Asp88 (E. coli) methylthiotransferase
Mfumv2_0699	2463	ftsK	240	394	194	344	258	628	DNA segregation ATPase FtsK/SpoIIIE/Cell division protein FtsK
Mfumv2_0703	822	his	177	97	93	55	317	258	Histidinol-phosphatase (EC 3.1.3.15)
Mfumv2_0706	1449	rho	700	694	440	472	973	1436	Transcription termination factor Rho
Mfumv2_0712	354	rbfA	602	107	353	68	585	155	Ribosome-binding factor A
Mfumv2_0713	2088	infB	1081	1503	862	1298	937	1938	Translation initiation factor IF-2
Mfumv2_0714	1257	nusA	1112	931	1107	1004	859	1071	Transcription elongation factor/Transcription termination protein NusA
Mfumv2_0719	615	rpoE	937	384	894	397	859	524	DNA-directed RNA polymerase specialized sigmasubunit/RNA polymerase sigma factor RpoE
Mfumv2_0723	186	rpsU	1897	235	2263	304	1908	352	30S ribosomal protein S21/SSU ribosomal protein S21p
Mfumv2_0733	492		79	0	203	0	72	0	Retron-type RNA-directed DNA polymerase (EC 2.7.7.49)
Mfumv2_0750	672		159	71	181	88	150	100	Glutamine amidotransferase, class I
Mfumv2_0773	858		49	28	53	33	53	45	putative Transglutaminase-like enzyme, cysteineprotease/transglutaminase domain protein
Mfumv2_0774	684	spr	59	26	49	24	69	47	Cell wall-associated hydrolase
Mfumv2_0785	2076	hyuA	126	174	87	130	227	468	N-methylhydantoinase A (EC 3.5.2.14)
Mfumv2_0786	1566	hyuB	123	125	186	208	210	324	N-methylhydantoinase B (EC 3.5.2.14)
Mfumv2_0795	1089	sgaA	376	272	303	237	796	856	Phosphoserine aminotransferase (EC 2.6.1.52)

Mfumv2_0811	1110	dinP	58	46	65	56	59	69	DNA polymerase IV (EC 2.7.7.7)
Mfumv2_0813	1005	queA	184	123	207	150	149	147	S-adenosylmethionine:tRNA ribosyltransferase-isomerase (EC 5.-.-.-)
Mfumv2_0821	1779	dnaX	327	370	255	311	473	798	DNA polymerase III subunits gamma and tau (EC 2.7.7.7)
Mfumv2_0847	849	ftsY	196	111	142	87	277	232	Signal recognition particle receptor protein FtsY (=alpha subunit) (TC 3.A.5.1.1)
Mfumv2_0857	861	holB	212	126	136	88	192	170	DNA polymerase III delta prime subunit (EC 2.7.7.7)
Mfumv2_0859	1407	cysS	156	124	90	77	171	202	Cysteinyl-tRNA synthetase (EC 6.1.1.16)
Mfumv2_0860	1422	lpd	78	74	63	65	234	330	Pyruvate/2-oxoglutarate dehydrogenase complex,dihydrolipoamide dehydrogenase (E3) component or relatedenzyme/Mercuric ion reductase (EC 1.16.1.1)
Mfumv2_0879	1044		160	111	133	100	120	124	3-isopropylmalate dehydrogenase
Mfumv2_0884	1140	dsbG	84	64	52	41	65	73	Protein-disulfide isomerase
Mfumv2_0893	693	nfi	130	60	102	51	486	334	Endonuclease V (EC 3.1.21.7)
Mfumv2_0912	696	dsbG	52	24	121	61	128	88	Periplasmic thiol:disulfide interchange protein DsbA
Mfumv2_0915	1386	rpoN	216	188	108	103	183	241	DNA-directed RNA polymerase specialized sigmasubunit/RNA polymerase sigma-54 factor RpoN
Mfumv2_0918	2748	alaS	319	579	255	503	343	933	Alanyl-tRNA synthetase (EC 6.1.1.7)
Mfumv2_0924	2460	glgP	255	418	210	372	362	884	Glycogen phosphorylase (EC 2.4.1.1)
Mfumv2_0934	561	efp	1740	650	1254	507	949	528	Translation elongation factor P
Mfumv2_0936	969	prs	392	253	320	224	826	793	Ribose-phosphate pyrophosphokinase (EC 2.7.6.1)
Mfumv2_0938	1236		411	335	274	245	756	924	Aspartate aminotransferase (EC 2.6.1.1)
Mfumv2_0940	1191	metK	782	619	524	451	820	968	S-adenosylmethionine synthetase (EC 2.5.1.6)
Mfumv2_0942	459		82	25	139	46	358	162	Cytochrome c family protein
Mfumv2_0949	426	rpsL	1339	380	748	230	1910	806	SSU ribosomal protein S12p (S23e)
Mfumv2_0951	471	rpsG	1948	611	2349	799	1608	751	SSU ribosomal protein S7p (S5e)
Mfumv2_0952	2172	fusA	1464	2079	1111	1710	1989	4205	Translation elongation factor G
Mfumv2_0953	306	rpsJ	1266	258	706	156	2449	743	SSU ribosomal protein S10p (S20e)
Mfumv2_0954	651	rplC	1444	680	909	464	866	608	LSU ribosomal protein L3p (L3e)
Mfumv2_0955	657	rplD	1341	583	1170	552	1078	692	LSU ribosomal protein L4p (L1e)
Mfumv2_0956	285	rplW	885	168	515	106	1023	288	LSU ribosomal protein L23p (L23Ae)
Mfumv2_0957	852	rplB	1729	981	907	557	973	821	LSU ribosomal protein L2p (L8e)
Mfumv2_0958	276	rpsS	1295	238	1184	235	1162	318	SSU ribosomal protein S19p (S15e)
Mfumv2_0959	339	rplV	1656	374	1278	313	2064	694	LSU ribosomal protein L22p (L17e)
Mfumv2_0960	648	rpsC	1893	817	1192	557	1556	1000	SSU ribosomal protein S3p (S3e)
Mfumv2_0961	426	rplP	2203	625	2483	763	3077	1300	LSU ribosomal protein L16p (L10e)
Mfumv2_0962	213	rpmC	2418	342	1671	257	2405	508	50S ribosomal protein L29
Mfumv2_0964	366	rplN	2586	527	1353	299	2340	709	LSU ribosomal protein L14p (L23e)
Mfumv2_0965	315	rplX	3703	777	1147	261	2094	653	LSU ribosomal protein L24p (L26e)
Mfumv2_0966	582	rplE	1559	592	782	322	1341	758	LSU ribosomal protein L5p (L11e)
Mfumv2_0967	387	rpsH	1036	267	762	213	808	310	SSU ribosomal protein S8p (S15Ae)
Mfumv2_0968	540	rplF	1357	488	1131	441	1302	697	LSU ribosomal protein L6p (L9e)
Mfumv2_0969	354	rplR	899	210	833	210	897	314	LSU ribosomal protein L18p (L5e)
Mfumv2_0970	639	rpsE	1722	733	1313	606	1577	998	SSU ribosomal protein S5p (S2e)
Mfumv2_0971	444	rplO	2776	816	1924	616	3527	1551	LSU ribosomal protein L15p (L27Ae)
Mfumv2_1011	168	rpmG	652	73	511	62	216	36	LSU ribosomal protein L33p @ LSU ribosomal protein L33p, zinc-independent
Mfumv2_1014	1671	glgP	144	165	129	160	125	213	Glycogen phosphorylase (EC 2.4.1.1)
Mfumv2_1016	1392	fumC	342	317	1022	443	451	622	Fumarate hydratase class II (EC 4.2.1.2)
Mfumv2_1027	426	rplK	1810	528	1129	357	2074	901	LSU ribosomal protein L11p (L12e)
Mfumv2_1028	720	rplA	1512	723	1300	675	2570	1835	LSU ribosomal protein L1p (L10Ae)
Mfumv2_1029	537	rplJ	682	244	737	286	1087	578	LSU ribosomal protein L10p (P0)
Mfumv2_1030	390	rplL	3815	991	4708	1325	7005	2709	LSU ribosomal protein L7/L12 (P1/P2)
Mfumv2_1031	3870	rpoB	1348	3463	1198	3319	1977	7561	DNA-directed RNA polymerase beta subunit (EC 2.7.7.6)
Mfumv2_1032	4173	rpoC	1442	4004	878	2646	1295	5359	DNA-directed RNA polymerase beta' subunit (EC 2.7.7.6)

Mfumv2_1035	810	proB	576	311	337	196	451	362	Glutamate 5-kinase (EC 2.7.2.11) / RNA-binding C-terminal domain PUA
Mfumv2_1036	1263	proA	424	339	280	243	430	511	Gamma-glutamyl phosphate reductase (EC 1.2.1.41)
Mfumv2_1045	903	hisG	927	541	1066	676	1667	1452	ATP phosphoribosyltransferase (EC 2.4.2.17)
Mfumv2_1047	1131	holA	54	41	61	50	37	41	DNA polymerase III, delta subunit
Mfumv2_1049	573	adk	204	78	172	71	169	96	Adenylate kinase (EC 2.7.4.3)
Mfumv2_1060	2799		144	265	98	198	84	234	Inactivated superfamily I helicase and RecB familyexonuclease
Mfumv2_1061	3186	recB	86	183	70	159	92	290	ATP-dependent exoDNAse (Exonuclease V) betasubunit
Mfumv2_1065	1296		625	653	788	893	564	878	HNH endonuclease
Mfumv2_1074	1464	atpD	151	147	195	206	176	255	ATP synthase beta chain (EC 3.6.3.14)
Mfumv2_1075	408	atpC	195	53	98	29	136	55	F0F1-type ATP synthase, epsilon subunit
Mfumv2_1076	279		194	36	1022	205	101	28	F0F1-type ATP synthase subunit, ATPase_gene1family
Mfumv2_1077	657	atpB	85	37	72	34	65	42	ATP synthase A chain (EC 3.6.3.14)
Mfumv2_1078	267	atpE	118	21	52	10	189	50	ATP synthase C chain (EC 3.6.3.14)
Mfumv2_1079	747	atpF	203	101	217	116	240	178	ATP synthase B chain (EC 3.6.3.14)
Mfumv2_1080	1515	atpA	154	155	79	86	178	267	ATP synthase alpha chain (EC 3.6.3.14)
Mfumv2_1081	885	atpG	149	80	81	47	264	211	ATP synthase gamma chain (EC 3.6.3.14)
Mfumv2_1082	1548	pckA	99	101	90	100	169	257	Phosphoenolpyruvate carboxykinase [ATP] (EC 4.1.1.49)
Mfumv2_1084	2202	gdb	82	121	58	91	136	297	Glycogen debranching enzyme
Mfumv2_1098	2370	xfp	197	311	230	393	199	466	Xylulose-5-phosphate phosphoketolase (EC 4.1.2.9); Fructose-6-phosphate phosphoketolase (EC 4.1.2.22)
Mfumv2_1101	2574	topA	317	544	189	351	387	987	DNA topoisomerase III (EC 5.99.1.2)
Mfumv2_1103	1083	aroB	128	88	154	115	179	182	3-dehydroquinate synthase (EC 4.2.3.4)
Mfumv2_1104	3486	dnaE	228	528	145	366	211	730	DNA polymerase III alpha subunit (EC 2.7.7.7)
Mfumv2_1105	1902	uvrD	105	133	86	118	146	276	ATP-dependent DNA helicase UvrD/PcrA
Mfumv2_1118	1575	nadB	192	201	177	201	386	602	L-aspartate oxidase (EC 1.4.3.16)
Mfumv2_1121	420	cccA	468	130	63	19	46	19	cytochrome c, class IC
Mfumv2_1122	699		198	89	24	12	43	30	Cytochrome c, class I
Mfumv2_1135	1446	icd	225	217	464	477	310	445	Isocitrate dehydrogenase [NADP] (EC 1.1.1.42)
Mfumv2_1159	864	pheA	78	45	45	28	100	86	Chorismate mutase I (EC 5.4.99.5) / Prephenate dehydratase (EC 4.2.1.51) # AroHI/PheAIp/ACT domain
Mfumv2_1162	1068		125	89	96	74	184	195	Agmatine deiminase (EC 3.5.3.12)
Mfumv2_1167	792	his	178	92	129	74	303	238	Histidinol-phosphatase (EC 3.1.3.15)
Mfumv2_1172	651		231	100	793	373	119	77	Cytochrome c family protein
Mfumv2_1191	897	mmsB	2221	1326	1959	1266	2187	1945	2-hydroxy-3-oxopropionate reductase (EC 1.1.1.60)
Mfumv2_1192	1386	lpd	575	531	354	354	527	724	Dihydrolipoamide dehydrogenase (EC 1.8.1.4)
Mfumv2_1193	1236	aceF	505	410	684	603	999	1209	Dihydrolipoamide acetyltransferase component of pyruvate dehydrogenase complex (EC 2.3.1.12)
Mfumv2_1196	978	acoB	827	538	541	379	1358	1315	Pyruvate dehydrogenase E1 component beta subunit (EC 1.2.4.1)
Mfumv2_1197	1074	acoA	1204	861	900	696	695	740	Pyruvate dehydrogenase E1 component alpha subunit (EC 1.2.4.1)
Mfumv2_1198	1365	gabT	93	85	68	67	83	112	4-aminobutyrate aminotransferase or relatedaminotransferase/aminotransferase class-III
Mfumv2_1209	252	rpsR	1787	294	912	165	948	237	SSU ribosomal protein S18p @ SSU ribosomal protein S18p, zinc-independent
Mfumv2_1211	1644	pgi	88	96	89	106	104	169	Glucose-6-phosphate isomerase (EC 5.3.1.9)
Mfumv2_1218	1851	glgB	137	157	120	149	181	308	Malto-oligosyltrehalose trehalohydrolase (EC 3.2.1.141)
Mfumv2_1223	1965	glgB	129	169	105	149	171	332	1,4-alpha-glucan (glycogen) branching enzyme, GH-13-type (EC 2.4.1.18)
Mfumv2_1227	2745	acn	225	409	114	226	268	729	Aconitate hydratase (EC 4.2.1.3)
Mfumv2_1231	768	tpiA	403	206	206	114	483	368	Triosephosphate isomerase (EC 5.3.1.1)
Mfumv2_1232	1215	pgk	445	360	224	197	731	881	Phosphoglycerate kinase (EC 2.7.2.3)
Mfumv2_1233	1044	cbbG	640	445	318	240	927	960	NAD-dependent glyceraldehyde-3-phosphate dehydrogenase (EC 1.2.1.12)
Mfumv2_1238	1107	trmU	454	334	263	209	362	397	tRNA-specific 2-thiouridylase MnmA
Mfumv2_1245	1185	sucC	220	174	293	250	353	415	Succinyl-CoA ligase [ADP-forming] beta chain (EC 6.2.1.5)
Mfumv2_1246	891	sucD	431	256	357	230	585	516	Succinyl-CoA ligase [ADP-forming] alpha chain (EC 6.2.1.5)
Mfumv2_1247	2073	aroA	372	512	250	375	515	1058	5-Enolpyruvylshikimate-3-phosphate synthase (EC 2.5.1.19) / Cytidylate kinase (EC 2.7.4.14)
Mfumv2_1256	1677	rpsA	1922	2147	1787	2163	1762	2930	30S ribosomal protein S1/SSU ribosomal protein S1p

Mfumv2_1259	483	cyoA	155	50	192	67	63	30	Heme/copper-type cytochrome oxidase, subunit 2/Cytochrome c oxidase (B(O/a)3-type) chain II (EC 1.9.3.1)
Mfumv2_1260	1686	cyoB	515	576	1087	1320	507	841	Heme/copper-type cytochrome oxidase, subunit 1/Cytochrome c oxidase (B(O/a)3-type) chain I (EC 1.9.3.1)
Mfumv2_1283	1287	lysA	468	420	201	196	301	402	Diaminopimelate decarboxylase (EC 4.1.1.20)
Mfumv2_1300	924	lysR	91	56	76	51	26	24	RuBisCO operon transcriptional regulator CbbR/transcriptional regulator, LysR family
Mfumv2_1331	1086	mipB	69	49	89	70	100	108	Transaldolase (EC 2.2.1.2)
Mfumv2_1333	282	rpoZ	1815	340	1910	389	1205	337	DNA-directed RNA polymerase omega subunit (EC 2.7.7.6)
Mfumv2_1353	1914	ftsH	313	377	201	261	234	420	ATP-dependent zinc metalloprotease FtsH 1/Cell division protein FtsH (EC 3.4.24.-)
Mfumv2_1355	801	rluA	1024	663	471	331	1477	1423	Ribosomal large subunit pseudouridine synthase D (EC 4.2.1.70)
Mfumv2_1361	474	cccA	2116	668	1972	675	1904	894	Cytochrome c oxidase polypeptide II (EC 1.9.3.1)
Mfumv2_1378	984	rpoD	56	36	62	44	77	75	DNA-directed RNA polymerase, sigma subunit(Sigma70/sigma32)
Mfumv2_1403	978	rpoD	532	318	795	513	280	249	DNA-directed RNA polymerase, sigma subunit(Sigma70/sigma32) RpoD
Mfumv2_1450	618	mcrA	262	86	65	23	178	87	Restriction endonuclease, McrA/HNH family
Mfumv2_1467	1374	glyQS	409	374	331	328	435	593	Glycyl-tRNA synthetase (EC 6.1.1.14)
Mfumv2_1469	975	nfo	98	57	76	48	99	86	Endonuclease IV (EC 3.1.21.2)
Mfumv2_1481	1239	thrC	279	230	170	152	345	423	Threonine synthase (EC 4.2.3.1)
Mfumv2_1483	243	napF	890	144	501	88	652	157	Ferredoxin
Mfumv2_1485	1842	greA	853	1046	644	856	826	1507	Transcription elongation factor GreA
Mfumv2_1487	1596	serA	744	791	874	1008	986	1560	D-3-phosphoglycerate dehydrogenase (EC 1.1.1.95)
Mfumv2_1491	603	phoE	722	290	604	263	888	531	Phosphoglycerate mutase (EC 5.4.2.1)
Mfumv2_1492	2040	cbbT	2819	3806	1813	2655	2777	5585	Transketolase (EC 2.2.1.1)
Mfumv2_1493	1023	udk	1391	933	853	620	3104	3102	Phosphoribulokinase/Uridine kinase (EC 2.7.1.48)
Mfumv2_1494	951	cbxXC	2824	1789	2351	1612	12032	11346	probable RuBisCo-expression protein CbbX
Mfumv2_1495	417	cbbS	6826	1894	3456	1041	8520	3521	Ribulose bisphosphate carboxylase small chain (EC 4.1.1.39)
Mfumv2_1496	1464	cbbL	7576	7387	4028	4255	6419	9318	Ribulose bisphosphate carboxylase large chain (EC 4.1.1.39)
Mfumv2_1497	1344	mipB	178	159	152	147	182	243	Transaldolase
Mfumv2_1498	333		577	128	553	133	2722	899	50S ribosomal protein L21 (fragment)/LSU ribosomal protein L21p
Mfumv2_1499	258	rpmA	855	147	521	97	2767	708	50S ribosomal protein L27/LSU ribosomal protein L27p
Mfumv2_1511	345	rplS	731	167	999	249	1219	415	50S ribosomal protein L19/LSU ribosomal protein L19p
Mfumv2_1513	258	rpsP	1478	254	1851	343	1935	495	30S ribosomal protein S16/SSU ribosomal protein S16p
Mfumv2_1530	1461	lysS	138	133	116	122	257	371	Lysyl-tRNA synthetase (class II) (EC 6.1.1.6)
Mfumv2_1547	1266	dinP	244	206	124	113	209	263	Nucleotidyltransferase/DNA polymerase UmuC/Error-prone, lesion bypass DNA polymerase V (UmuC)
Mfumv2_1578	1119		176	131	157	127	184	204	Serine--pyruvate aminotransferase (EC 2.6.1.51) / L-alanine:glyoxylate aminotransferase (EC 2.6.1.44)
Mfumv2_1585	2142	glgX	307	438	151	233	287	609	Glycogen debranching enzyme (EC 3.2.1.-)
Mfumv2_1614	1065	aroF	1211	858	820	629	739	780	2-keto-3-deoxy-D-arabino-heptulosonate-7-phosphate synthase I alpha (EC 2.5.1.54)
Mfumv2_1620	483	cyoA	2229	716	1347	470	29	14	Heme/copper-type cytochrome oxidase, subunit 2
Mfumv2_1621	1686	cyoB	1475	1649	1058	1284	13077	21758	Cytochrome c oxidase (B(O/a)3-type) chain I (EC 1.9.3.1)
Mfumv2_1635	1104	glpX	666	489	508	404	449	492	Fructose-1,6-bisphosphatase, GlpX type (EC 3.1.3.11)
Mfumv2_1643	1164		509	395	344	289	800	923	Aspartate aminotransferase (EC 2.6.1.1)
Mfumv2_1661	438	dtd	27	8	25	8	28	12	D-tyrosyl-tRNA(Tyr) deacylase
Mfumv2_1678	585	rpoE	1084	431	1046	451	635	376	RNA polymerase sigma factor RpoE
Mfumv2_1686	1113	ftsZ	277	204	183	147	233	257	Cell division protein FtsZ (EC 3.4.24.-)
Mfumv2_1687	1239	ftsA	236	195	255	228	241	296	Cell division ATPase FtsA
Mfumv2_1688	870	ftsQ	261	151	204	128	180	154	Cell division septal protein FtsQ
Mfumv2_1693	1182	ftsW	131	103	110	94	118	138	Cell division protein FtsW
Mfumv2_1702	1488	murE	171	169	151	161	191	281	UDP-N-acetylmuramoylalanyl-D-glutamate--2,6-diaminopimelate ligase (EC 6.3.2.13)
Mfumv2_1704	1815	ftsI	224	272	221	292	193	350	Cell division protein FtsI [Peptidoglycan synthetase] (EC 2.4.1.129)
Mfumv2_1707	441		1038	304	1196	381	759	332	Cell division protein MraZ
Mfumv2_1714	1050	ctaA	505	351	467	354	139	145	Heme A synthase, cytochrome oxidase biogenesis protein Cox15-CtaA
Mfumv2_1719	1833	typA	580	706	390	516	562	1021	GTP-binding protein TypA/BipA
Mfumv2_1727	1299		76	66	71	67	90	116	Nuclease of restriction endonuclease-like fold,RmuC family/DNA recombination protein RmuC

Mfumv2_1728	2154	ppk	210	301	105	162	199	423	Polyphosphate kinase (EC 2.7.4.1)
Mfumv2_1729	396	rplQ	1202	317	1273	364	1064	418	50S ribosomal protein L17/LSU ribosomal protein L17p
Mfumv2_1730	1014	rpoA	1278	840	1285	914	2235	2187	DNA-directed RNA polymerase alpha subunit (EC 2.7.7.6)
Mfumv2_1731	609	rpsD	1699	689	1189	522	2161	1304	30S ribosomal protein S4/SSU ribosomal protein S4p (S9e)
Mfumv2_1732	543	rpsK	3478	1258	2348	920	3256	1751	Ribosomal protein S11/SSU ribosomal protein S11p (S14e)
Mfumv2_1733	396	rpsM	2510	661	1399	400	2386	937	30S ribosomal protein S13/SSU ribosomal protein S13p (S18e)
Mfumv2_1735	270	rpsT	295	53	123	24	1143	306	30S ribosomal protein S20/SSU ribosomal protein S20p
Mfumv2_1736	1743	argS	96	111	284	356	141	244	Arginyl-tRNA synthetase (EC 6.1.1.19)
Mfumv2_1745	1395	bioA	140	129	56	56	143	198	Adenosylmethionine-8-amino-7-oxononanoate aminotransferase (EC 2.6.1.62)
Mfumv2_1751	996	asd	529	351	457	329	649	641	Aspartate-semialdehyde dehydrogenase (EC 1.2.1.11)
Mfumv2_1785	678	nagB	130	58	122	59	327	217	6-phosphogluconolactonase (EC 3.1.1.31), eukaryotic type
Mfumv2_1786	1569	zwf	173	180	199	226	273	424	Glucose-6-phosphate 1-dehydrogenase (EC 1.1.1.49)
Mfumv2_1805	2079	nadE	175	243	138	206	462	938	NAD synthetase (EC 6.3.1.5) / Glutamine amidotransferase chain of NAD synthetase
Mfumv2_1815	3072		58	115	77	166	69	206	Proline dehydrogenase (EC 1.5.99.8) / Delta-1-pyrroline-5-carboxylate dehydrogenase (EC 1.5.1.12)
Mfumv2_1826	5091		418	1416	665	2444	658	3319	putative Transcription elongation factor GreA/GreBdomain protein/DNA helicase
Mfumv2_1834	870		165	127	158	131	148	168	2-hydroxy-3-oxopropionate reductase (EC 1.1.1.60)
Mfumv2_1844	663	rplY	969	428	950	453	1576	1036	50S ribosomal protein L25/LSU ribosomal protein L25p
Mfumv2_1846	336	rpsF	858	192	692	168	930	310	30S ribosomal protein S6/SSU ribosomal protein S6p
Mfumv2_1848	579	rplI	801	305	902	377	1416	813	50S ribosomal protein L9/LSU ribosomal protein L9p
Mfumv2_1852	1317	thrA	294	258	308	293	455	593	Homoserine dehydrogenase (EC 1.1.1.3)
Mfumv2_1853	1101	thrC	356	259	249	197	501	546	Threonine synthase (EC 4.2.3.1)
Mfumv2_1883	510		53	18	16	6	53	27	Uri superfamily endonuclease (modular protein)
Mfumv2_1894	768	amiC	272	139	310	170	293	222	N-acetylmuramoyl-L-alanine amidase (EC 3.5.1.28)
Mfumv2_1895	612	tsf	1305	532	701	310	1213	736	Translation elongation factor Ts
Mfumv2_1896	747	rpsB	1606	798	1114	600	2630	1948	30S ribosomal protein S2/SSU ribosomal protein S2p (SAe)
Mfumv2_1904	1299	potE	164	136	152	135	66	80	amino acid permease family protein
Mfumv2_1905	1326	bioA	108	95	96	91	103	136	Adenosylmethionine-8-amino-7-oxononanoate aminotransferase (EC 2.6.1.62)
Mfumv2_1912	2646	valS	242	425	138	263	321	842	Valyl-tRNA synthetase (EC 6.1.1.9)
Mfumv2_1914	1878	rpoD	1363	1701	828	1120	1665	3099	RNA polymerase sigma factor RpoD
Mfumv2_1915	1776	dnaG	92	109	90	115	149	263	DNA primase (EC 2.7.7.-)
Mfumv2_1925	1083	trpD	172	124	137	107	264	283	Anthranilate phosphoribosyltransferase (EC 2.4.2.18)
Mfumv2_1927	1320	pyrC	181	159	136	130	304	398	Dihydroorotase (EC 3.5.2.3)
Mfumv2_1928	981	pyrB	57	37	85	60	197	191	Aspartate carbamoyltransferase (EC 2.1.3.2)
Mfumv2_1929	549	leuD	388	142	421	167	593	323	3-isopropylmalate dehydratase small subunit (EC 4.2.1.33)
Mfumv2_1930	1113	leuB	444	329	415	332	796	879	3-isopropylmalate dehydrogenase (EC 1.1.1.85)
Mfumv2_1933	1158	tyrS	417	321	398	333	475	546	Tyrosyl-tRNA synthetase (EC 6.1.1.1)
Mfumv2_1962	1326		34	30	28	27	34	45	putative Glycine/d-amino acid oxidases(Deaminating)/FAD dependent oxidoreductase
Mfumv2_1971	441	cynS	129	38	94	30	222	97	Cyanate hydratase (EC 4.2.1.104)
Mfumv2_1981	1245	argE	122	101	93	84	159	196	N-carbamoyl-L-amino acid hydrolase (EC 3.5.1.87)
Mfumv2_1988	876	cfxP	626	365	419	265	953	828	Phosphoribulokinase (EC 2.7.1.19)
Mfumv2_1989	1071	fbp	540	378	665	504	815	849	Fructose-1,6-bisphosphatase, type I (EC 3.1.3.11)
Mfumv2_1993	1524	proS	182	185	161	177	302	456	Prolyl-tRNA synthetase (EC 6.1.1.15)
Mfumv2_1994	288	gatC	224	48	142	33	361	114	Aspartyl-tRNA(Asn) amidotransferase subunit C (EC 6.3.5.6)
Mfumv2_1995	1452	gatA	272	263	228	239	532	766	Aspartyl-tRNA(Asn) amidotransferase subunit A (EC 6.3.5.6)
Mfumv2_1996	1458	gatB	487	473	292	308	638	923	Aspartyl-tRNA(Asn) amidotransferase subunit B (EC 6.3.5.6)
Mfumv2_1998	2076	ligA	327	452	534	798	232	477	DNA ligase (EC 6.5.1.2)
Mfumv2_2007	972	trpS	397	256	307	215	583	560	Tryptophanyl-tRNA synthetase (EC 6.1.1.2)
Mfumv2_2008	816		230	125	188	111	305	247	Chorismate mutase I (EC 5.4.99.5)
Mfumv2_2010	741	aroD	63	31	95	51	95	70	3-dehydroquinate dehydratase I (EC 4.2.1.10)
Mfumv2_2011	1137	mreB	1136	859	1059	870	937	1056	Rod shape-determining protein MreB

Mfumv2_2012	912	mreC	383	238	217	146	376	348	Rod shape-determining protein MreC
Mfumv2_2014	2028	ftsI	399	539	354	519	287	578	Cell division protein FtsI [Peptidoglycan synthetase] (EC 2.4.1.129)
Mfumv2_2015	1182	rodA	113	89	104	89	90	105	Rod shape-determining protein RodA
Mfumv2_2017	1266	serS	439	369	367	334	423	522	Seryl-tRNA synthetase (EC 6.1.1.11)
Mfumv2_2019	276		1414	259	1480	294	775	212	Translation initiation factor IF-1 (modularprotein)
Mfumv2_2037	1515	leuA	879	885	507	552	1158	1740	2-isopropylmalate synthase (EC 2.3.3.13)
Mfumv2_2038	1029	ilvC	690	473	488	362	1211	1236	Ketol-acid reductoisomerase (EC 1.1.1.86)
Mfumv2_2039	474	ilvH	545	172	315	108	340	160	Acetolactate synthase small subunit (EC 2.2.1.6)
Mfumv2_2058	1134	pstS	74	56	53	43	74	83	Phosphate ABC transporter, periplasmic phosphate-binding protein PstS (TC 3.A.1.7.1)
Mfumv2_2059	1026	pstC	107	72	94	70	166	169	Phosphate transport system permease protein PstC (TC 3.A.1.7.1)
Mfumv2_2060	840	pstA	46	26	94	57	62	52	Phosphate transport system permease protein PstA (TC 3.A.1.7.1)
Mfumv2_2061	822	pstB	139	76	96	57	217	177	Phosphate transport ATP-binding protein PstB (TC 3.A.1.7.1)
Mfumv2_2063	762	sdhB	341	173	122	67	269	202	Succinate dehydrogenase iron-sulfur protein (EC 1.3.99.1)
Mfumv2_2064	1956	sdhA	367	476	200	281	390	755	Succinate dehydrogenase flavoprotein subunit (EC 1.3.99.1)
Mfumv2_2065	690	sdhC	114	45	154	66	144	84	Succinate dehydrogenase cytochrome b subunit
Mfumv2_2083	1914	resB	316	402	232	321	263	500	ResB protein required for cytochrome cbiosynthesis
Mfumv2_2084	1050	pheS	232	162	189	143	309	322	Phenylalanyl-tRNA synthetase alpha chain (EC 6.1.1.20)
Mfumv2_2085	2706	ileS	273	492	280	547	343	919	Isoleucyl-tRNA synthetase (EC 6.1.1.5)
Mfumv2_2120	1125	gltA	131	98	121	98	653	729	2-methylcitrate synthase (EC 2.3.3.5)
Mfumv2_2140	1275	eno	472	401	407	375	586	740	Enolase (EC 4.2.1.11)
Mfumv2_2141	375		304	76	229	62	237	87	Septum formation initiator/Cell division protein DivIC (FtsB), stabilizes FtsL against RasP cleavage
Mfumv2_2161	1284	argB	150	128	93	86	90	113	N-acetylglutamate synthase related protein
Mfumv2_2164	975	fbaA	949	616	703	494	1053	1018	Fructose-bisphosphate aldolase class II (EC 4.1.2.13)
Mfumv2_2170	2085	dinG	38	51	37	54	128	256	DinG family ATP-dependent helicase YoaA
Mfumv2_2185	1512	zwf	318	320	234	255	311	466	Glucose-6-phosphate 1-dehydrogenase (EC 1.1.1.49)
Mfumv2_2186	1482	gppA	124	121	113	121	169	248	Exopolyphosphatase (EC 3.6.1.11)
Mfumv2_2190	2364	metE	563	887	561	956	1003	2352	5-methyltetrahydropteroyltriglutamate--homocysteine methyltransferase (EC 2.1.1.14)
Mfumv2_2194	2520	glgP	596	999	418	760	438	1093	Glycogen phosphorylase (EC 2.4.1.1)
Mfumv2_2202	1176	guaB	481	377	258	219	1086	1265	IMP dehydrogenase subunit
Mfumv2_2203	1563	guaA	343	357	284	321	661	1024	GMP synthase [glutamine-hydrolyzing] (EC 6.3.5.2)
Mfumv2_2204	1281	hisD	370	314	301	276	462	583	Histidinol dehydrogenase (EC 1.1.1.23)
Mfumv2_2205	1098		2147	1570	1783	1413	1470	1599	ATP:guanido phosphotransferase
Mfumv2_2214	897	mmsB	85	51	131	85	208	185	3-hydroxyisobutyrate dehydrogenase (EC 1.1.1.31)
Mfumv2_2220	969	cysK	713	460	510	357	865	831	Cysteine synthase (EC 2.5.1.47)
Mfumv2_2221	861	rpoD	783	449	1254	780	1075	918	RNA polymerase sigma factor RpoD
Mfumv2_2229	936	miaA	83	52	58	39	48	45	tRNA dimethylallyltransferase (EC 2.5.1.75)
Mfumv2_2235	1128	nuoH	197	148	335	273	468	524	NADH-ubiquinone oxidoreductase chain H (EC 1.6.5.3)
Mfumv2_2236	1707	nuoG	810	921	660	814	1161	1964	NADH-ubiquinone oxidoreductase chain G (EC 1.6.5.3)
Mfumv2_2237	1371	nuoF	945	863	461	456	1108	1505	NADH-ubiquinone oxidoreductase chain F (EC 1.6.5.3)
Mfumv2_2238	534	nuoE	824	292	407	157	1192	631	NADH-ubiquinone oxidoreductase chain E (EC 1.6.5.3)
Mfumv2_2239	1257	nuoD	598	501	343	309	1210	1506	NADH-ubiquinone oxidoreductase chain D (EC 1.6.5.3)
Mfumv2_2240	639	nuoC	954	406	416	192	1168	740	NADH-ubiquinone oxidoreductase chain C (EC 1.6.5.3)
Mfumv2_2241	513	nuoB	781	267	575	213	1014	516	NADH-ubiquinone oxidoreductase chain B (EC 1.6.5.3)
Mfumv2_2243	1125	hisC	113	85	134	109	205	229	Biosynthetic Aromatic amino acid aminotransferase beta (EC 2.6.1.57)
Mfumv2_2244	858	tyrA	145	83	90	56	230	196	Prephenate dehydrogenase (EC 1.3.1.12)
Mfumv2_2247	885	ilvE	273	159	264	169	336	290	Branched-chain amino acid aminotransferase (EC 2.6.1.42)
Mfumv2_2253	1416	gnd	58	55	82	84	164	230	6-phosphogluconate dehydrogenase, decarboxylating (EC 1.1.1.44)
Mfumv2_2254	444	rpiB	649	171	577	185	375	164	Ribose 5-phosphate isomerase B (EC 5.3.1.6)
Mfumv2_2273	873	proC	186	108	116	73	296	256	Pyrroline-5-carboxylate reductase (EC 1.5.1.2)
Mfumv2_2274	528	aroK	201	68	139	51	489	245	Shikimate kinase I (EC 2.7.1.71)

Mfumv2_2275	1089	aroC	299	217	146	115	326	352	Chorismate synthase (EC 4.2.3.5)
Mfumv2_2279	1308	glgC	115	100	119	112	153	197	Glucose-1-phosphate adenylyltransferase (EC 2.7.7.27)
Mfumv2_2281	1347	gltX	420	340	370	325	572	689	Glutamyl-tRNA synthetase (EC 6.1.1.17) @ Glutamyl-tRNA(Gln) synthetase (EC 6.1.1.24)
Mfumv2_2294	960	fmt	251	168	241	175	366	365	Methionyl-tRNA formyltransferase (EC 2.1.2.9)
Mfumv2_2296	981	sua	129	84	227	161	88	86	Sua5 YciO YrdC YwlC family protein
Mfumv2_2298	2607	gyrA	699	1213	681	1280	442	1143	DNA gyrase subunit A (EC 5.99.1.3)
Mfumv2_2299	2523	gyrB	906	1523	678	1234	605	1511	DNA gyrase subunit B (EC 5.99.1.3)
Mfumv2_2319	1065	hemA	185	131	108	83	277	293	Glutamyl-tRNA reductase (EC 1.2.1.70)
Mfumv2_2320	810	ccmC	217	113	209	121	225	180	ABC-type transport system involved in cytochrome cbiogenesis, permease component/HemX protein, negative effector of steady-state concentration of glutamyl-tRNA reductase
Mfumv2_2328	1620		201	217	176	206	202	325	Pyruvate decarboxylase (EC 4.1.1.1); Alpha-keto-acid decarboxylase (EC 4.1.1.-)
Mfumv2_2337	1425	nuoN	239	227	331	341	489	690	NADH-ubiquinone oxidoreductase chain N (EC 1.6.5.3)
Mfumv2_2339	1467	nuoM	232	226	403	427	386	562	NADH-ubiquinone oxidoreductase chain M (EC 1.6.5.3)
Mfumv2_2341	1806	nuoL	512	619	601	786	458	824	NADH-ubiquinone oxidoreductase chain L (EC 1.6.5.3)
Mfumv2_2342	306	nuoK	240	49	158	35	432	131	NADH-ubiquinone oxidoreductase chain K (EC 1.6.5.3)
Mfumv2_2343	498	nuoJ	410	136	297	107	626	309	NADH-ubiquinone oxidoreductase chain J (EC 1.6.5.3)
Mfumv2_2344	519	nuoI	596	206	363	136	713	365	NADH-ubiquinone oxidoreductase chain I (EC 1.6.5.3)
Mfumv2_2350	582	thiJ	657	246	268	109	474	265	putative intracellular protease/amidase
Mfumv2_2354	615		79	33	62	28	100	62	Cytochrome c553
Mfumv2_2370	1110	trpE	130	96	102	81	254	280	Para-aminobenzoate synthase, aminase component (EC 2.6.1.85)
Mfumv2_2378	1767	ilvD	602	705	547	696	698	1223	Dihydroxy-acid dehydratase (EC 4.2.1.9)
Mfumv2_2379	1338	trmFO	165	149	193	189	161	216	Methylenetetrahydrofolate--tRNA-(uracil-5-)-methyltransferase TrmFO/tRNA:m(5)U-54 MTase gid
Mfumv2_2380	2328	pheT	1213	1878	1927	3224	1587	3654	Phenylalanyl-tRNA synthetase beta chain (EC 6.1.1.20)
Mfumv2_2382	645	trmB	333	143	406	189	269	172	tRNA (guanine46-N7-)-methyltransferase (EC 2.1.1.33)
Mfumv2_2389	774	hisF	511	280	1178	700	497	407	Imidazole glycerol phosphate synthase cyclase subunit (EC 4.1.3.-)
Mfumv2_2390	522	cspR	88	29	92	33	147	71	tRNA (cytidine(34)-2'-O)-methyltransferase (EC 2.1.1.207) ## TrmL
Mfumv2_2392	774	truA	111	57	84	47	278	213	tRNA pseudouridine synthase A (EC 4.2.1.70)
Mfumv2_2395	822	trpA	161	88	123	73	166	135	Tryptophan synthase alpha chain (EC 4.2.1.20)
Mfumv2_2401	612	hisB	314	128	267	118	391	237	Imidazoleglycerol-phosphate dehydratase (EC 4.2.1.19)
Mfumv2_2410	1086		40	26	23	16	41	40	DegT/DnrJ/EryC1/StrS aminotransferase
Mfumv2_2414	1146	napF	352	265	126	102	255	287	Ferredoxin
Mfumv2_2417	1149	napF	280	214	163	135	149	170	Ferredoxin
Mfumv2_2422	1818		105	127	75	98	303	545	Pyruvate kinase family protein
Mfumv2_2439	1899	mnmG	230	304	116	165	470	925	tRNA uridine 5-carboxymethylaminomethyl modification enzyme GidA
Mfumv2_2440	1449	dnaB	137	133	102	107	267	386	Replicative DNA helicase
Mfumv2_2446	555		285	95	373	135	711	351	Cytochrome c family protein (modular protein)
Mfumv2_2448	906	thrB	91	55	38	25	118	106	Homoserine kinase (EC 2.7.1.39)
Mfumv2_2452	564		545	194	443	171	763	404	Predicted GTPase
Mfumv2_2454	858	atpB	922	526	934	577	1717	1461	ATP synthase A chain (EC 3.6.3.14)
Mfumv2_2455	213	atpE	1071	150	598	92	3972	839	ATP synthase C chain (EC 3.6.3.14)
Mfumv2_2456	531	atpF	2013	711	1494	572	3815	2009	ATP synthase B chain (EC 3.6.3.14)
Mfumv2_2457	396	atpH	603	159	392	112	1826	717	ATP synthase delta chain (EC 3.6.3.14)
Mfumv2_2458	1554	atpA	876	905	981	1099	2286	3518	ATP synthase alpha chain (EC 3.6.3.14)
Mfumv2_2459	882	atpG	560	301	518	302	1533	1226	ATP synthase gamma chain (EC 3.6.3.14)
Mfumv2_2460	1404	atpD	852	796	1099	1112	1819	2533	ATP synthase beta chain (EC 3.6.3.14)
Mfumv2_2461	438	atpC	1248	364	809	256	1421	617	ATP synthase epsilon chain (EC 3.6.3.14)
Mfumv2_2463	936	mesJ	197	123	104	70	637	590	tRNA(Ile)-lysidine synthase
Mfumv2_2466	1686	ccmC	112	126	108	131	285	476	ABC-type transport system involved in cytochrome cbiogenesis, permease component
Mfumv2_2508	873	cysE	175	102	102	64	198	171	Serine acetyltransferase (EC 2.3.1.30)
Mfumv2_2509	888	aroE	194	116	192	125	238	212	Shikimate 5-dehydrogenase I alpha (EC 1.1.1.25)
